# Host genetic variants in sepsis risk: a field synopsis and meta-analysis

**DOI:** 10.1186/s13054-019-2313-0

**Published:** 2019-01-25

**Authors:** Hongxiang Lu, Dalin Wen, Xu Wang, Lebin Gan, Juan Du, Jianhui Sun, Ling Zeng, Jianxin Jiang, Anqiang Zhang

**Affiliations:** 1 State Key Laboratory of Trauma, Burns and Combined Injury, Research Institute of Surgery, Daping Hospital, Army Medical University, Changjiang Branch Road 10, Daping Street, Yuzhong District, Chongqing, 400042 China; 2 Department of Emergency Surgery, The Second Affiliated Hospital, Zhejiang University, Hangzhou, 310009 Zhejiang China; 3 Department of Emergency Surgery, The Affiliated Hospital, Guizhou Medical University, Guiyang, 550004 Guizhou China

**Keywords:** Variant, Sepsis, Meta-analysis, Systematic review

## Abstract

**Background:**

Published data revealed that host genetic variants have a substantial influence on sepsis susceptibility. However, the results have been inconsistent. We aimed to systematically review the published studies and quantitatively evaluate the effects of these variants on the risk of sepsis.

**Methods:**

We searched the PubMed, EMBASE, Medline, Web of Knowledge, and HuGE databases to identify studies that investigated the associations between genetic variants and sepsis risk. Then, we conducted meta-analyses of the associations for genetic variants with at least three study populations and applied the Venice criteria to assess the association result credibility.

**Results:**

A literature search identified 349 eligible articles that investigated 405 variants of 172 distinct genes. We performed 204 primary and 185 subgroup meta-analyses for 76 variants of 44 genes. The results showed that 29 variants of 23 genes were significantly associated with the risk of sepsis, including 8 variants of pattern recognition receptors (PRRs), 14 variants of cytokines, one variant of an immune-related gene and 6 variants of other genes. Furthermore, the cumulative epidemiological evidence of a significant association between each variant and the risk of sepsis was classified as strong or moderate for 18 variants. For the 329 variants with fewer than three study populations, 63 variants of 48 genes have been reported to be significantly associated with the risk of sepsis in a systematic review.

**Conclusion:**

We identified several genetic variants that could influence the susceptibility to sepsis by systematic review and meta-analysis. This study provides a comprehensive overview of the genetic architecture of variants involved in sepsis susceptibility and novel insight that may affect personalized targeted treatment in the future clinical management of sepsis.

**Electronic supplementary material:**

The online version of this article (10.1186/s13054-019-2313-0) contains supplementary material, which is available to authorized users.

## Background

Sepsis is defined as a life-threatening organ dysfunction that is caused by a dysregulated host response to infection, and it is a major, underrecognized health-care problem worldwide [1]. Despite modern resuscitating strategies and new anti-infective options, sepsis remains the leading cause of death in critically ill patients [2]. The international expert consensus is that the heterogeneity of patients with sepsis might be in part responsible for the failure of clinical trials of treatments for patients with sepsis [3]. Therefore, reliable markers that can identify high-risk patients are urgently needed to improve detection and preventive care. Sorensen and colleagues [4] reported that adult adoptees had a 5.81-fold increased risk of dying from infection if one of their biologic parents died of infection before the age of 50. This risk exceeded the relative risk (RR) of dying of cancer or cardiovascular disease, suggesting for the first time that host genetics might play an important role in the development of infectious diseases. Since then, increasing evidence has suggested that genetic variants, particularly single nucleotide polymorphisms (SNPs), are critical determinants of inter-individual differences in both inflammatory responses and clinical outcomes in sepsis patients [5].

Recently, many investigators have reported associations between host genetic variants containing SNPs, insertion/deletions (Ins/Del), and variable numbers of tandem repeats (VNTRs) in genes that regulate the host’s immune response and sepsis susceptibility [6, 7]. However, due to power considerations in single variant studies with relatively small sample sizes, the outcomes of these studies remain contradictory, and no systematic review investigating all tested variants has been published thus far.

The present study applied a systematic review and meta-analysis approach with a predefined protocol to clarify the potential associations between host genetic variants and the risk of sepsis.

## Methods

This search was conducted according to the PRISMA criteria [8]. A written protocol with predefined eligibility criteria and bibliographic search terms was developed and used for all subsequent procedures.

### Search strategy

**Fig. 1:**
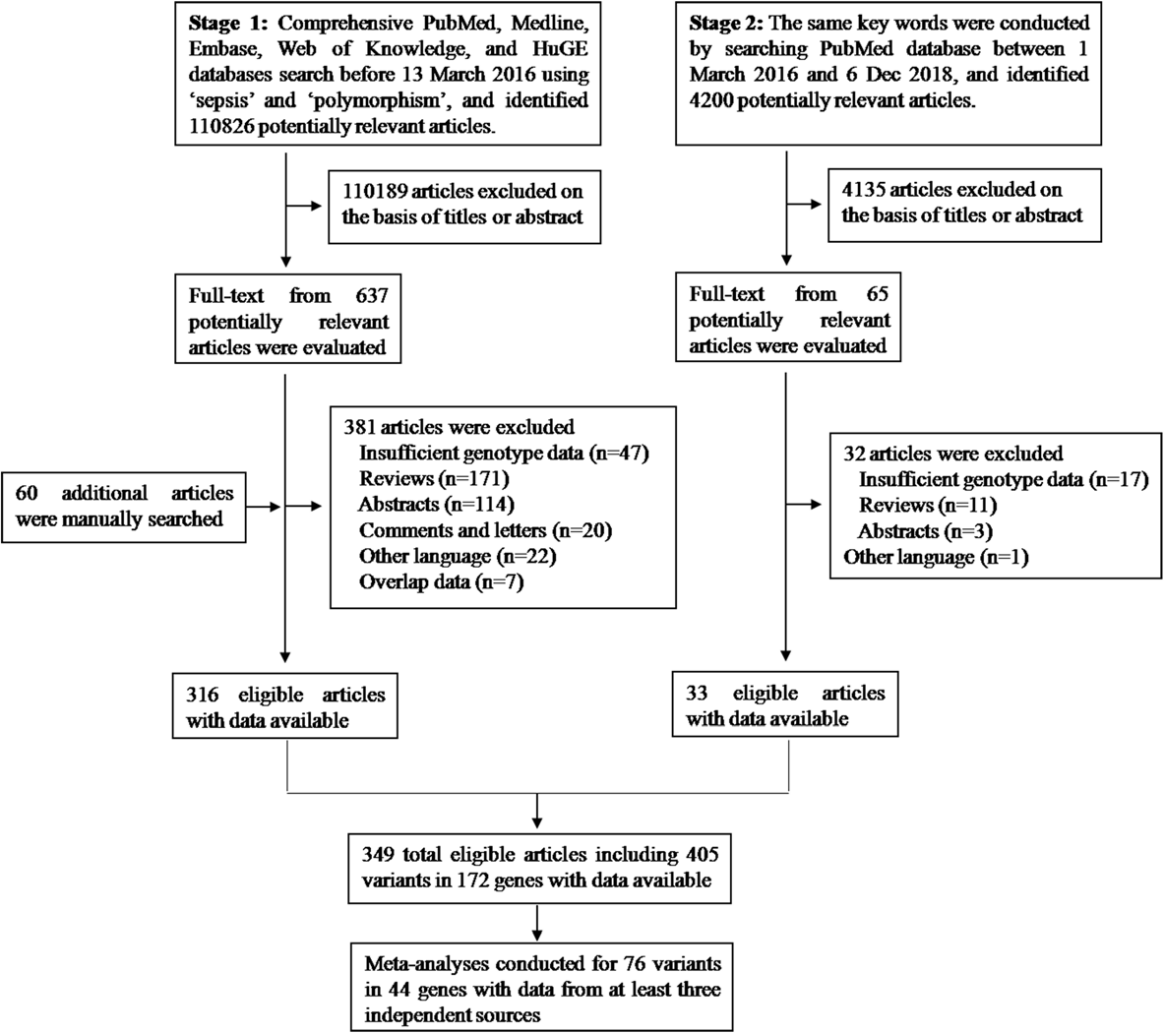
Flow diagram of study identification, inclusion, and exclusion

We used a two-stage search strategy to identify relevant publications (Fig. 1). In stage 1, we constructed a search protocol, including keywords associated or synonymous with the search terms “sepsis” and “polymorphism” in the PubMed, EMBASE, Medline, Web of Knowledge, and HuGE databases (see the Additional file 1 for the complete search strategy). The search was performed on March 7, 2016. The references of all identified publications were also searched for additional eligible studies. In stage 2, which was conducted on March 1, 2016, through December 6, 2018, we used the same key words to query the PubMed database.

### Eligibility criteria

We included full-length studies that evaluated the associations between sepsis and genetic variants. The following inclusion criteria were applied: (1) the study was an independent case-control or cohort design and evaluated the association between genetic polymorphisms and sepsis susceptibility; (2) the study had sufficient data for calculating an odds ratio (OR) with 95%confidence intervals (CIs); and (3) the study was published in English. The exclusion criteria were as follows: (1) the study was a case report, abstract, comment, letter, or review; (2) the study lacked sufficient data and the relative risk could not be calculated after contacting the authors; and (3) the genotype data overlapped with that of other studies.

To minimize bias and improve reliability, two researchers (Lu HX and Zhang AQ) independently screened the titles and abstracts with the same inclusion and exclusion criteria. The final inclusion of studies was determined by consensus between the researchers.

### Data extraction

Data were independently extracted and evaluated by two researchers (Zeng L and Wen DL) who used standardized data extraction forms. One third of the extracted data were also evaluated by another researcher (Sun JH), with any disagreements being resolved by consensus. For each study, information such as the name of the first author, publication year, country/region origin, ethnicity of the study population, study design (population-based versus hospital-based), genotyping method, source of controls, genotyped data of cases and controls, and frequencies of the genotypes were collected. When studies included subjects of different ethnicities, the data pertaining to different populations were treated as separate studies. When required, supplemental data were obtained by contacting the authors. Whenever possible, we also evaluated Hardy-Weinberg equilibrium (HWE) based on the genotype distribution among control subjects.

### Statistical analysis

In the meta-analysis, we only included genetic variants for which at least three studies provided data due to the limited validity of pooled results with a small number of studies. The strength of the association between a given polymorphism and the risk of sepsis was represented by the OR and 95% CI. The pooled ORs were calculated based on the dominant (BB+AB versus AA), recessive (BB versus AB+AA), and allelic genetic models (B versus A) (A represented the major allele, B represented the minor allele). The significance of the pooled ORs was examined by the Z test (*P* < 0.05 was considered significant, which was not adjusted due to insufficient data and the lack of a uniform population). We did not use adjusted ORs to estimate the pooled ORs since inconsistent covariates were used for adjustment in the original studies included in this meta-analysis. Strong associations with sepsis risk were defined as ORs ≥ 1.5 or ≤ 0.67. Moderate associations were defined as ORs = 1.5–1.15 or 0.67–0.87, according to the recommendations in Ma’s study [9]. Additional, a strong association of a genetic variant with the risk of sepsis was defined by a *p* value < 0.001. Heterogeneity between studies was assessed using a *χ*^2^-based *Q*-test [10]. *P* > 0.05 indicated a lack of heterogeneity across studies, allowing us to use the fixed effects model (the Mantel-Haenszel method). Otherwise, the random effects model was used (the DerSimonianand Laird method) [11]. If the data permitted it, subgroup analyses were performed according to age (adult and pediatric) and ethnicity (white and Asian populations). Ethnicity was categorized as “white” and “Asian”, according to the ethnic groups reported in studies. When a study did not state the ethnic groups but stated the nationality of the participants, we considered populations of Chinese, Japanese, Korean, Indian, and Thai origin to be “Asian”. Populations of Turkish, Italian, Russian, and French Canadian origin were considered to be “White.” If it was impossible to separate participants according to ethnicity, the participants were considered to be a “mixed population.” Publication bias was evaluated by Egger’s test, with *P* < 0.10 being considered clear bias [12]. The Venice criteria were applied to the cumulative epidemiological evidence for each genetic variant to evaluate the strength of the evidence supporting its significant association with sepsis risk [13, 14]. According to the criteria (see Additional file 1 for the Venice criteria), the credibility level of the cumulative evidence was defined as strong (A grades only), weak (one or more C grades), or moderate (all other combinations). All the *P* values presented are nominal *P* values and are not corrected for multiple comparisons. All statistical tests were performed with STATA, version 11.0 (Stata Corporation, College Station, TX, USA). To ensure the accuracy and reliability of the results, two researchers (Lu HX and Zhang AQ) entered the data into the software independently and reached a consensus.

## Results

### Characteristics of eligible studies

Figure 1 shows the flow diagram detailing the process of trial identification and selection. Finally, 349 articles were eligible for inclusion in this analysis. The characteristics of the eligible studies are shown in Additional file 2: Table S1. Overall, data on 405 genetic variants of 172 distinct genes were available (Additional file 3: Table S2). The sample sizes in the included studies ranged from 42 to 18,358 (cases: 16–5468, median: 275). Approximately 95% of the eligible studies (331/349) were published after 2002 (with a steep positive trend over time; Additional file 4: Figure S1). With regard to ethnicity, 68% of the studies were in white populations. The majority of the studies (60%) were hospital-based case-control studies, and the remaining were population-based case-control studies. Data were also available from one GWAS [15].

The types of variants were mainly SNPs (*n* = 379, 94%), followed by insertion/deletions (*n* = 17) and VNTRs (*n* = 9). These genetic variants were located upstream of the gene (including the promoter region) (*n* = 83), in the 5′UTR (*n* = 15), in exons (*n* = 113), in introns (*n* = 149), in the 3′UTR (*n* = 34), and downstream of the gene (*n* = 11). Among the exonic SNPs, the functional effects were generally missense (*n* = 96) and synonymous coding changes (*n* = 12), with the remaining being frameshift, stop-gain, or splicing variants (*n* = 5). The distribution of these variants across chromosomes is depicted in Additional file 5: Figure S2. Of note, most of the variants were located on chromosome 6 (*n* = 48), none were on chromosome 21 and Y, and only 2 variants were in mitochondrial DNA.

The majority of candidate genes were PPRs (*n* = 22, 12.8%), cytokines (*n* = 39, 22.7%), and other immune-related genes. Based on the number of different independent studies of each variant (ranging from 1 to 41), the 5 most commonly studied variants were the following: TNFA rs1800629 (*n* = 41), LTA rs909253 (*n* = 32), TLR4rs4986790 (*n* = 28), IL6 rs1800795 (*n* = 25), and IL10 rs1800896 (*n* = 24). Based on the country/region of origin, most studies were performed in China (*n* = 82), the USA (*n* = 40), Germany (*n* = 33), and Spain (*n* = 22). Based on the number of subjects (cases plus controls) enrolled (ranging from 42 to 18,358, the median number being 720), the 5 most commonly studied variants were as follows: F5 rs6025 (*n* = 18,358 individuals), TNFA rs1800629 (*n* = 15,057 individuals), LTA rs909253 (*n* = 12,185 individuals), MBL2 A/O haplotype (*n* = 9066 individuals), and IL6 rs1800795 (*n* = 8630 individuals). TaqMan probes, polymerase chain reaction-restriction fragment length polymorphism (PCR-RFLP), allele-specific amplification, and sequencing were the most common methods used to determine the genotype distribution.

### Meta-analysis findings

In total, 389 meta-analyses were performed for 76 variants of 44 genes, with at least 3 independent studies for each variant. The major results of the meta-analysis under three different genetic models are listed in Table 1 and Additional file 6: Table S3. Of the 389 meta-analyses, 204 were primary meta-analyses, and 185 were meta-analyses of subgroups defined by ethnicity (white (*n* = 64) vs. Asian (*n* = 28)) and age (adult (*n* = 61) vs. pediatric (*n* = 32)).

**Table 1:** Genetic variants nominally significantly associated with sepsis risk in meta-analyses

Genes	Variant ID	Chr	Risk allele	Studies	Cases	Controls	Group	Genetic models	OR (95% CI)	*P* value	*I*^2^ (%)	P_heterogeneity_	Level of evidence	Venice criteria
TLR1	rs5743551	4	G	4	1224	943	All/Adult	R	1.52(1.16–2.00)	0.002	0	0.54	Moderate	BAA
TLR1	rs5743551	4	G	4	1224	943	All/Adult	A	1.17(1.02–1.34)	0.02	0	0.42	Strong	AAA
TLR1	rs5743551	4	G	3	978	492	White	R	1.78(1.18–2.69)	0.006	0	0.59	Moderate	BAA
TLR1	rs5743551	4	G	3	978	492	White	A	1.24(1.04–1.47)	0.02	0	0.41	Weak	BAC
TLR2	rs5743708	4	A	10	948	1573	Adult	D	1.75(1.11–2.75)	0.02	30	0.19	Moderate	BBA
TLR2	rs5743708	4	A	7	720	695	Adult	A	2.04(1.20–3.48)	0.009	0	0.45	Moderate	BAA
TLR2	rs5743708	4	G	4	308	1087	Pediatric	D	0.43(0.20–0.89)	0.02	0	0.56	Moderate	BAA
TLR2	rs5743708	4	G	4	308	1087	Pediatric	A	0.43(0.21–0.90)	0.03	0	0.57	Strong	AAA
LBP	rs2232618	20	C	4	615	1267	All	D	1.93(1.49–2.48)	< 0.0001	0	0.41	Moderate	BAA
LBP	rs2232618	20	C	3	501	738	All/Adult	A	2.10(1.58–2.78)	< 0.0001	0	0.62	Moderate	BAA
RAGE	rs1800625	6	T	4	977	1339	All	D	0.59(0.48–0.73)	< 0.0001	0	0.69	Strong	AAA
RAGE	rs1800625	6	T	4	977	1339	All	R	0.47(0.24–0.96)	0.04	0	0.79	Strong	AAA
RAGE	rs1800625	6	T	4	977	1339	All	A	0.61(0.51–0.74)	< 0.0001	0	0.78	Strong	AAA
RAGE	rs1800624	6	T	3	662	838	All	A	0.78(0.63–0.97)	0.02	10	0.33	Strong	AAA
NOD2	rs2066844	16	T	3	229	943	All/White	D	2.25(1.39–3.64)	0.001	0	0.55	Moderate	BAA
NOD2	rs2066847	16	insC	5	326	1629	All/White	D	2.37(1.49–3.75)	0.0002	0	0.58	Moderate	BAA
MBL2	A/O haplotype	10	O	23	3549	5517	All/White	D	1.25(1.05–1.48)	0.01	63	< 0.001	Weak	ACC
MBL2	A/O haplotype	10	O	16	3353	4765	All/White	A	1.19(1.04–1.36)	0.01	57	0.002	Weak	ACC
MBL2	A/O haplotype	10	O	7	307	960	Pediatric	D	1.61(1.23–2.12)	0.0006	24	0.24	Moderate	BAB
MBL2	A/O haplotype	10	O	3	176	445	Pediatric	A	1.40(1.03–1.91)	0.03	0	0.72	Moderate	BAA
TNFA	rs1799724	6	T	3	502	1373	All/Adult	D	1.29(1.01–1.64)	0.04	0	0.761	Weak	BAC
TNFA	rs1800629	6	A	41	5540	9709	All	D	1.35(1.13–1.62)	0.001	71	< 0.001	Weak	ACC
TNFA	rs1800629	6	A	33	4859	8386	All	R	1.32(1.05–1.67)	0.02	16	0.23	Moderate	BAA
TNFA	rs1800629	6	A	33	4859	8386	All	A	1.21(1.04–1.42)	0.02	66	< 0.001	Weak	ACB
TNFA	rs1800629	6	A	12	1241	1715	Asian	D	2.16(1.75–2.67)	< 0.0001	37	0.09	Moderate	BBA
TNFA	rs1800629	6	A	11	1209	1645	Asian	R	2.56(1.44–4.57)	0.001	0	0.77	Weak	CAC
TNFA	rs1800629	6	A	11	1209	1645	Asian	A	1.99(1.64–2.42)	< 0.0001	30	0.16	Moderate	ABA
TNFA	rs1800629	6	A	30	4114	5983	Adult	D	1.51(1.20–1.90)	< 0.0001	74	< 0.001	Weak	ACC
TNFA	rs1800629	6	A	25	3515	4751	Adult	R	1.49(1.12–1.97)	0.005	17	0.24	Moderate	BAA
TNFA	rs1800629	6	A	25	3515	4751	Adult	A	1.30(1.06–1.61)	0.01	70	< 0.001	Weak	ACA
TNFA	rs361525	6	A	5	808	1457	All/Adult	R	4.88(2.19–10.87)	< 0.0001	0	0.85	Weak	CAA
TNFA	rs361525	6	A	4	745	903	Asian	D	2.05(1.06–3.96)	0.03	65	0.04	Weak	BCC
TNFA	rs361525	6	A	4	745	903	Asian	A	1.79(1.39–2.30)	< 0.0001	18	0.3	Moderate	BAA
LTA	rs909253	6	T	30	4662	7473	All	D	0.83(0.71–0.97)	0.02	64	< 0.001	Weak	ACA
LTA	rs909253	6	T	27	4512	6649	All	A	0.87(0.79–0.97)	0.01	58	< 0.001	Weak	ACA
LTA	rs909253	6	T	8	946	1355	Asian	R	0.78(0.63–0.96)	0.02	0	0.57	Weak	BAC
LTA	rs909253	6	T	6	846	1240	Asian	A	0.86(0.76–0.97)	0.02	24	0.25	Strong	AAA
LTA	rs909253	6	T	27	4012	6659	Adult	D	0.79(0.67–0.94)	0.007	65	< 0.001	Weak	ACA
LTA	rs909253	6	T	26	3962	5953	Adult	R	0.85(0.75–0.96)	0.008	4	0.41	Strong	AAA
LTA	rs909253	6	T	24	3862	5835	Adult	A	0.84(0.75–0.94)	0.002	55	0.001	Weak	ACA
IL1B	rs143634	2	C	7	1095	1168	All	R	0.53(0.34–0.82)	0.004	0	0.84	Moderate	AAB
IL1B	rs143634	2	C	5	969	1004	White	R	0.51(0.32–0.80)	0.003	0	0.82	Strong	AAA
IL1B	rs143634	2	C	5	879	744	Adult	R	0.54(0.33–0.90)	0.02	0	0.7	Strong	AAA
IL6	rs1800796	7	G	3	298	341	Asian	R	0.53(0.29–0.96)	0.04	0	0.99	Weak	BAC
IL6	rs1800796	7	G	3	478	364	Adult	D	0.55(0.42–0.74)	< 0.0001	63	0.07	Weak	BCA
IL6	rs1800795	7	C	17	1903	4573	All/White	A	1.17(1.01–1.35)	0.03	61	0.001	Weak	ACA
IL6	rs1800795	7	C	11	1452	2786	Adult	R	1.43(1.19–1.73)	< 0.0001	42	0.06	Weak	BBC
IL6	rs1800795	7	C	10	1405	2723	Adult	A	1.24(1.06–1.46)	0.008	53	0.02	Weak	ACA
IL8	rs4073	4	T	11	1314	1605	All/adult	D	0.71(0.60–0.84)	< 0.0001	43	0.06	Moderate	ABA
IL8	rs4073	4	T	10	1224	1515	All/adult	R	0.71(0.52–0.97)	0.03	47	0.05	Moderate	ABA
IL8	rs4073	4	T	10	1224	1515	All/adult	A	0.76(0.63–0.92)	0.004	60	0.008	Weak	ACA
IL8	rs4073	4	T	7	830	1074	White	D	0.68(0.56–0.84)	0.0002	45	0.09	Moderate	BBA
IL8	rs4073	4	T	7	830	1074	White	A	0.74(0.58–0.95)	0.02	66	0.007	Weak	ACA
IL10	rs1800871	1	T	6	686	764	Asian	R	1.35(1.03–1.76)	0.03	37	0.16	Moderate	BBA
IFNG	rs2430561	12	T	5	715	759	All/Adult	D	1.34(1.07–1.68)	0.01	46	0.11	Moderate	ABA
CXCL1	rs1429638	4	A	3	589	1108	All/Asian/Adult	R	0.68(0.56–0.83)	< 0.0001	0	0.84	Moderate	BAA
CXCL1	rs1429638	4	A	3	589	1108	All/Asian/Adult	A	0.75(0.64–0.88)	< 0.0001	0	0.86	Moderate	AAB
CXCL12	rs266087	10	A	3	542	1006	All/Asian/Adult	R	0.75(0.57–0.99)	0.04	58	0.09	Weak	ACA
CXCL12	rs2297630	10	A	3	589	1109	All/Asian/Adult	R	0.63(0.50–0.79)	< 0.0001	0	0.9	Moderate	BAB
CXCL12	rs2297630	10	A	3	589	1109	All/Asian/Adult	A	0.67(0.54–0.82)	< 0.0001	0	0.89	Moderate	BAA
MIF	rs755622	22	G	4	408	399	All	R	0.42(0.22–0.79)	0.007	52	0.1	Weak	BCC
FCGR2A	rs1801274	1	G	8	754	1307	All/White	R	1.65(1.08–2.53)	0.02	68	0.003	Weak	BCC
FCGR2A	rs1801274	1	G	8	754	1307	All/White	A	1.25(1.09–1.44)	0.001	44	0.09	Moderate	ABA
PAI-1	rs1799768	7	4G	11	1962	1340	All/White	R	1.50(1.09–2.06)	0.01	69	0.0004	Weak	BCC
PAI-1	rs1799768	7	4G	10	1277	1275	All/White	A	1.32(1.01–1.72)	0.04	80	< 0.0001	Weak	ACC
PAI-1	rs1799768	7	4G	4	563	603	Pediatric	A	1.37(1.02–1.85)	0.04	65	0.03	Weak	ACC
ACE	rs4646994	17	ins	9	1139	3107	All	R	0.79(0.67–0.92)	0.003	43	0.08	Moderate	ABA
ACE	rs4646994	17	ins	4	534	781	Adult	R	0.73(0.58–0.92)	0.008	0	0.44	Moderate	BAA
ACE	rs4646994	17	ins	4	534	781	Adult	A	0.81(0.69–0.94)	0.008	43	0.15	Moderate	ABA
LCE4A	rs4845320	1	C	3	320	240	All	D	2.75(1.36–5.57)	0.005	0	0.47	Weak	CAA
LCE4A	rs4845320	1	C	3	320	240	All	A	2.77(1.41–5.44)	0.003	12	0.32	Weak	CAA
TAGAP	rs3127214	6	T	3	317	239	All	D	1.84(1.07–3.17)	0.03	15	0.31	Weak	CAA
TAGAP	rs3127214	6	T	3	317	239	All	A	2.01(1.20–3.36)	0.008	21	0.28	Weak	CAA
VDR	rs2228570	12	T	4	240	244	All	D	2.94(1.85–4.71)	< 0.0001	0	0.85	Moderate	BAA
VDR	rs2228570	12	T	3	180	184	White	D	3.15(1.87–5.31)	< 0.0001	0	0.78	Moderate	BAA
VDR	rs2228570	12	T	3	180	184	White	R	2.96(1.18–4.92)	< 0.0001	31	0.24	Weak	CBA
VDR	rs2228570	12	T	3	200	204	Adult	D	2.95(1.85–4.71)	< 0.0001	0	0.69	Weak	BAC
MIR608	rs4919510	5	C	3	502	766	All/Asian/Adult	D	1.82(1.39–2.38)	< 0.0001	0	0.85	Moderate	BAA
MIR608	rs4919510	5	C	3	502	766	All/Asian/Adult	R	1.34(1.03–1.75)	0.03	0	0.79	Moderate	BAA
MIR608	rs4919510	5	C	3	502	766	All/Asian/Adult	A	1.39(1.19–1.63)	< 0.0001	0	0.81	Strong	AAA

*D* dominant model, *R* recessive model, *A* allelic model; A/O haplotype: combination of Arg52Cys, Gly54Asp, and Gly57GLu

Of the 204 primary meta-analyses performed, nominally significant associations (*P* < 0.05) with the risk of sepsis were found with 26 (34%) variants of 21 genes for at least one genetic model containing TLR1 rs5743551-7202A/G; LBP rs2232618 Phe436Leu; the MBL2 A/O haplotype; RAGE rs1800625-429 T/C and rs1800624-374 T/A; NOD2 rs2066844 Arg702Trp and rs2066847 Leu1007Pro; TNFA rs1799724-857C/T, rs1800629-308G/A, and rs361525-238G/A; LTA rs909253+252 T/C; IL1B rs143634+3594C/T; IL6 rs1800795-174G/C; IL8 rs4073-251 T/A; IFNG rs2430561+874A/T; CXCL1 rs1429638A/C; CXCL12 rs266087A/G and rs2297630A/G; MIF rs755622-173G/C; FCGR2A rs1801274 His131Arg; PAI-1 rs1799768-6755G/4G; ACE rs4646994ins/del; LCE4A rs4845320A/C; TAGAP rs3127214C/T; VDR rs2228570 Met1Thr; and miR-608 rs4919510G/C. The 185 subgroup meta-analyses by ethnicity and age revealed three additive polymorphisms (TLR2 rs5743708 Arg753Gln, IL6 rs1800796-572G/C, and IL10 rs1800896-1082A/G) that were significantly associated with the risk of sepsis. Strong associations with sepsis (ORs ≥ 1.5 or ≤ 0.67) were detected in 14 variants. Moderate associations with sepsis (ORs 1.5–1.15 or 0.67–0.87) were found for the remaining 15 variants. Ten of the 29 positive variants showed a highly significant association with sepsis risk, with *p* < 0.001; 11 showed an association with sepsis risk with *p* = 0.001–0.01, and the remaining 8 had *p* < 0.05.

Of the 389 meta-analyses of all available data, 154 (39.6%) had no or little heterogeneity (*I*^2^ < 25%), 92 (23.7%) had a moderate level of heterogeneity (25% < *I*^2^ < 50%), and 143 (36.7%) had a high degree of heterogeneity (*I*^2^ > 50%). The proportion of studies with a high degree of heterogeneity was significantly lower for the 29 positive variants than the remaining 47variants (30.9% vs. 39.5%, Fisher’s exact *p* < 0.05).

To assess the cumulative epidemiologic evidence for the 29 positive variants identified through the primary and subgroup analyses, the Venice criteria were applied. According to the Venice criteria, A grades were given to 37, 45, and 58 meta-analyses for the amount of evidence, replication of association, and protection from bias, respectively. B grades were given to 36, 13, and 5 meta-analyses for the same categories, respectively. C grades were given to 7, 22, and 17 meta-analyses for these three criteria, respectively. Next, strong, moderate, and weak levels of evidence of a significant association with sepsis risk were assigned to 4 (TLR1 rs5743551-7202A/G, RAGE rs1800625-429 T/C and rs1800624-374 T/A, and miR-608 rs4919510G/C), 14, and 11 variants, respectively. The details of the nominally significant associations found in the meta-analysis are discussed below.

### Polymorphisms in PRRs

Toll-like receptors (TLRs) are a crucial family of pathogen-recognition receptors (PRRs) that provide a major mechanism for innate immune cells to recognize and respond to pathogens [16]. TLR1/2 is responsible for recognizing cell wall components of gram-positive bacteria (lipopeptides, peptidoglycan, and lipoteichoic acid). TLR4 is responsible for recognizing the lipopolysaccharide of gram-negative bacteria. Increasing evidence indicates that polymorphisms of TLR genes influence susceptibility to various infectious diseases, including sepsis [6]. According to the primary meta-analysis, four studies (including 1224 cases and 943 controls) were performed to determine the association between TLR1rs5743551-7202A/G and the risk of sepsis. There was a significantly increased risk of sepsis under the recessive (OR = 1.52, 95% CI = 1.16–2.00, *P* = 0.002) and allelic models (OR = 1.17, 95% CI = 1.02–1.34, *P* = 0.02). When subgroups were considered, data from three white populations revealed more significant associations (OR = 1.78, 95% CI = 1.18–2.69, *P* = 0.006 for the recessive model and OR = 1.24, 95% CI = 1.04–1.47, *P* = 0.02 for the allelic model). However, the level of evidence was intermediate due to the study sample size. For rs5743708 Arg753Gln in the TLR2 gene, there was no significant association found in the overall analysis of 14 studies. However, the association between Arg753Gln and the risk of sepsis was significant in the adult population (OR = 1.75, 95% CI = 1.11–2.75, *P* = 0.02 for the dominant model and OR = 2.04, 95% CI = 1.20–3.48, *P* = 0.009 for the allelic model). In contrast, there was a marginally significantly decreased risk of sepsis associated with Arg753Gln in pediatric studies (OR = 0.43, 95% CI = 0.20–0.89, *P* = 0.02 for the dominant model and OR = 0.43, 95% CI = 0.21–0.90, *P* = 0.03 for the allelic model), which may be due to differences between the adult and pediatric populations.

The lipopolysaccharide-binding protein (LBP) is an enhancer of the host response to lipopolysaccharides that acts by facilitating the transfer of lipopolysaccharides to CD14 membrane-bound TLRs that ultimately activate signaling transduction pathways and the production of cytokines [17]. The primary meta-analysis of LBP rs2232618 Phe436Leu included four studies (615 cases and 1267 controls). There was a significantly increased risk of sepsis associated with this variant in the dominant model (OR = 1.93, 95% CI = 1.49–2.48, *P* < 0.0001). Furthermore, through the subgroup analysis, strong associations were found in three adult populations (OR = 2.10, 95% CI = 1.58–2.78, *P* < 0.0001 for the allelic model).

The receptor for advanced glycation end products (RAGE) is a member of the immunoglobulin protein family of cell surface molecules and has been shown to bind a diverse set of ligands, and this binding leads to the activation of several pro-inflammatory signaling pathways [18]. Two variants located in the promoter region of RAGE were significantly associated with the risk of sepsis. In total, four studies on RAGE rs1800625-429 T/C were conducted by Zeng et al [19] and Shao et al [20]. The primary meta-analysis of the four studies showed significant associations between the risk of sepsis and RAGE rs1800625-429 T/C in all three genetic models (OR = 0.59, 95% CI = 0.45–0.73, *P* < 0.001 for the dominant model, OR = 0.47, 95%CI = 0.24–0.96, *P* = 0.04 for the recessive model, and OR = 0.61, 95%CI = 0.51–0.74, *P* < 0.0001 for the allelic model) with a high level of evidence. Moreover, in vitro LPS-induced RAGE expression and TNF-α production were significantly lower in the variant C allele patients than in those with the wild-type T allele [21]. With regard to another RAGE variant, rs1800624-374 T/A, a significant association was observed only in the allelic model (OR = 0.78, 95% CI = 0.63–0.97, *P* = 0.02) in three studies.

NOD2/CARD15 is a member of a superfamily of genes, the NBS-LRR proteins (for nucleotide-binding sites and leucine-rich repeats), which are involved in the intracellular recognition of microbes and their products [22]. Three studies (including 229 cases and 943 controls) determined the association between NOD2 rs2066844 Arg702Trp and the risk of sepsis. There was an increased risk of sepsis associated with NOD2 rs2066844 Arg702Trp in the dominant model (OR = 2.25, 95% CI = 1.39–3.64, *P* = 0.001). For an additional variant, rs2066847 Leu1007Pro, five studies (326 cases and 1629controls) were included in our meta-analysis. A significant association between rs2066847 Leu1007Pro and the risk of sepsis was confirmed in the dominant model (OR = 2.37, 95% CI = 1.49–3.75, *P* = 0.0002). Mannose-binding lectin (MBL) is a soluble pattern recognition molecule that binds microorganisms, thereby activating the lectin or additional complement pathways [23]. Three polymorphisms in the MBL2 gene result in three variant structural alleles (B, C, and D), which are referred to collectively as O, while A is the wild-type allele. Twenty-three studies analyzed the MBL2 A/O haplotype. The overall results showed a significant association between MBL2 A/O and the risk of sepsis (OR = 1.25, 95% CI = 1.05–1.48, *P* = 0.01 for the dominant model; OR = 1.19, 95% CI = 1.04–1.36, P = 0.01 for the allelic model). The significant association was stronger when the meta-analysis was restricted to pediatric sepsis (OR = 161, 95% CI = 1.23–2.12, *P* = 0.0006 for the dominant model; OR = 1.40, 95% CI = 1.03–1.91, *P* = 0.03 for the allelic model).

### Polymorphisms in cytokines

Cytokines released from immune cells are major players in the inflammatory response to infection. Primary pro-inflammatory cytokines, such as tumor necrosis factor alpha (TNF-α) and interleukin-1 (IL-1), induce secondary pro- and anti-inflammatory mediators such as IL-6 and IL-10 [24]. Genetic variants of these cytokines have been shown to play key roles in determining susceptibility to sepsis [6].

Forty-one studies investigated the association between TNFA rs1800629-308G/A and the risk of sepsis (5540 cases and 9709 controls). In the primary meta-analysis, significant associations between the -308G/A polymorphism and the risk of sepsis were found under all three genetic models. Furthermore, through stratified analysis according to ethnicity and age, the associations were more significant in the adult and Asian populations than in the overall populations, suggesting that this SNP might be specifically linked to the adult and Asian populations. In our study, data on the second TNFA variant (rs1799724-857C/T) from three studies (502 cases and 1373 controls) were meta-analyzed. The overall results showed that there was a significant association between this variant and the risk of sepsis under the dominant model (OR = 1.29, 95% CI = 1.01–1.64, *P* = 0.04) with a low level of evidence. Associations between a third TNFA variant (rs361525-238G/A) and the risk of sepsis were investigated in nine studies. The primary meta-analysis indicated a highly significant association between the TNFA rs361525-238G/A variant and the risk of sepsis under the recessive model (OR = 4.88, 95% CI = 2.19–10.87, *P* < 0.0001). Furthermore, in the analysis stratified by ethnicity, only Asian individuals with this variant had a higher risk of sepsis than individuals with the wild-type allele under the dominant and allelic genetic models. The LTA rs909253+252 T/C variant was analyzed in 32 studies with 4762 cases and 7588 controls and was significantly associated with the risk of sepsis under the dominant and allelic models; however, there was a low level of evidence due to between-study heterogeneity. The associations were also significant in the analyses stratified by ethnicity and age.

Seven studies investigated the IL1B rs143634+3594C/T variant. The result of the overall comparison suggested that the association of this variant with sepsis susceptibility was significant under the recessive genetic model (OR = 0.53, 95% CI = 0.34–0.82, *P* = 0.034). Meanwhile, the stratified analysis showed that there was a significant association between this variant and the risk of sepsis in the white population (OR = 0.51, 95% CI = 0.32–0.80, *P* = 0.003 for the recessive model) and the adult population (OR = 0.54, 95% CI = 0.33–0.90, *P* = 0.02 for the recessive model). With regard to rs1800796-572G/C, which is located in the IL-6 promoter region, there was no significant association with the risk of sepsis in the primary meta-analysis. However, three studies in Asian populations indicated a significant association between -572G/C and the risk of sepsis (OR = 0.53, 95% CI = 0.29–0.96, *P* = 0.04 for the recessive model). In the adult population, the association between this variant and the risk of sepsis was also confirmed under the dominant model. A significantly increased risk of sepsis was found to be associated with another variant, rs1800795-174G/C, in the allelic model (OR = 1.17, 95% CI = 1.01–1.35, *P* = 0.03) through the primary meta-analysis of 17 studies and the subgroup analysis of adults (OR = 1.43, 95% CI = 1.19–1.73, *P* < 0.0001 for the recessive model and OR = 1.24, 95% CI = 1.06–1.46, *P* = 0.008 for the allelic model). For IL-8rs4073-251 T/A, the association between the variant and the risk of sepsis were confirmed under all genetic models in the primary analysis and the subgroup analysis according to ethnicity. For the IL10 rs1800871-819C/T variant, a significant association with the susceptibility to sepsis was only confirmed in the Asian population (OR = 1.35, 95% CI = 1.03–1.76, *P* = 0.03 for the recessive model).

Five studies of the IFNG rs2430561-874A/T variant were included in the meta-analysis. The results of the overall comparison suggested that the association of this variant with the risk of sepsis was significant under the dominant genetic model (OR = 1.34, 95% CI = 1.07–1.68, *P* = 0.01). The rs1429638A/C variant of the CXCL1 gene and the rs2297630A/G and rs266087A/G polymorphisms of the CXCL12 gene were found to be associated with altered susceptibility to traumatic sepsis in Wang’s study (accepted by Journal of Trauma Acute Care Surg). Four studies of MIF-173G/C were included in the meta-analysis. A significant association between this variant and the susceptibility to sepsis was found under the recessive model (OR = 0.42, 95% CI = 0.22–0.79, *P* = 0.007).

### Polymorphisms in immunity

FcγRIIA (CD32A) is a tyrosine-based activation motif-bearing immunoreceptor that binds immunoglobulin G and C-reactive protein, which are important opsonins in the host defense response [25]. In total, eight studies on FCGR2A rs1801274 His131Arg were eligible for the meta-analysis. The results of the primary meta-analysis suggested that the association of this variant with sepsis susceptibility was significant (OR = 1.65, 95% CI = 1.08–2.53, *P* = 0.02 for the recessive model and OR = 1.25, 95% CI = 1.09–1.44, *P* = 0.001 for the allelic model).

### Polymorphisms in other genes

Eleven studies determined the association between PAI-1-675 5G/4G and the risk of sepsis in white populations. There was a significantly increased risk of sepsis associated with this variant in the recessive model (OR = 1.50, 95% CI = 1.09–2.06, *P* = 0.01) and the allelic model (OR = 1.32, 95% CI = 1.01–1.72, *P* = 0.04). Furthermore, in the subgroup analysis stratified by age, a significant association was also found in the pediatric population (OR = 1.37, 95% CI = 1.02–1.85, *P* = 0.04 for allelic effect). The rs4646994ins/del polymorphism in the ACE gene was significantly associated with the risk of sepsis in the recessive model (OR = 0.79, 95% CI = 0.67–0.92, *P* = 0.003) according to a primary meta-analysis of nine studies; this association was stronger in the adult population than in the pediatric population. Three studies evaluated the association between the LCE4A rs4845320 polymorphism and the risk of sepsis. There were significant associations between this variant and an increased risk of sepsis under the dominant (OR = 2.75, 95% CI = 1.36–5.57, *P* = 0.005) and allelic models (OR = 2.77, 95% CI = 1.41–5.44, *P* = 0.003). For the TAGAP rs3127214C/T variant, there was a significantly increased risk of sepsis under the dominant (OR = 1.84, 95% CI = 1.07–3.17, *P* = 0.03) and allelic models (OR = 2.01, 95% CI = 1.20–3.36, *P* = 0.008). For the VDR rs2228570 Met1Thr variant, a statistically significant association with an increased risk of sepsis was found under the dominant model (OR = 2.94, 95% CI = 1.85–4.71, *p* < 0.0001) according to the primary meta-analysis of four studies; this association was also identified in the white and adult populations in the subgroup analyses. Finally, three studies evaluated the association between the miR-608 rs4919510G/C variant and the risk of sepsis, and the meta-analysis results showed that the association was significant under all three genetic models.

### Non-significant associations identified through the meta-analysis

The vast majority of meta-analyses (127 primary and 94 subgroup meta-analyses) performed in this study (47 variants of 33 genes) did not yield any evidence of a significant association. These meta-analyses included a median of 4 studies (range 3–28) and 1750 participants (range 289–18,358).

### Polymorphisms not included in the meta-analysis

In this study, 329 polymorphisms could not be meta-analyzed because fewer than three studies investigated them. We systematically reviewed the associations between these polymorphisms and the risk of sepsis, and nominally statistically significant associations with the susceptibility to sepsis were found for 63 polymorphisms in 48 genes (*p* < 0.05) (Additional file 7: Table S4). The samples sizes ranged from 80 to 1895. These functional genes encoded PRRs (*n* = 10), signaling molecules (*n* = 5), transcription factors (*n* = 3), cytokines (*n* = 11), and other molecules (*n* = 19). Furthermore, most of these genetic variants were located in the promoter region (*n* = 16), introns (*n* = 23), and 3′UTR (*n* = 7); 13 variants were missense. Furthermore, some genetic variants (*n* = 10) were also confirmed to be functional variants. For example, the TLR4 rs10116253-2242C allele enhances the transcriptional activities and expression of TLR4, and trauma patients with the variant C allele have a greater capacity to produce the pro-inflammatory cytokines TNF-α and IL-6 than those with other alleles [26]. After exposure to LPS, TOLLIP expression levels from homozygotes for the rs5743867C allele were significantly higher than those from other genotypes. Moreover, the concentrations of TNF-α and IL-6 were significantly lower in the culture supernatants from subjects with the rs5743867CC genotype than in those from subjects with the CT and TT genotypes [27]. Genetic variants in the miRNA region also affected the risk of sepsis. Patients with the rs2910164 GG/GC genotypes had higher levels of mature miR-146a than those with the rs2910164 CC genotype, which was related to the excessive inflammation in patients with severe sepsis [28]. Therefore, further studies are needed to investigate the functional roles of these polymorphisms in the pathophysiology of sepsis and to validate these encouraging results in independent cohorts.

## Discussions

Sequence variants within genes have been considered candidates for the promotion of sepsis pathogenesis. In the present study, we reviewed all available studies on the associations between genetic polymorphisms and the risk of sepsis and conducted systematic reviews and meta-analyses. We evaluated data regarding 405 variants located in 172 candidate genes from 349 eligible articles published in the past two decades. We identified 29 variants of 23 genes that were significantly associated with the susceptibility to sepsis through primary meta-analyses and subgroup meta-analyses stratified by ethnicity (white vs. Asian) and age (adult vs. pediatric). Furthermore, an additional 63 variants of 48 genes were found to be significantly associated with the risk of sepsis by systematic review. These findings provide investigators with a robust platform of genetic variant information that may be useful in the attempts to elucidate the molecular mechanisms underlying the pathogenesis of sepsis.

Variants are alterations in nucleotide base pairs located within a gene or noncoding portion of the genome [29]. In sepsis, the focus has been on understanding the impact of genetic variants on the innate and adaptive immune signaling pathways. Candidate gene association studies have focused on variants in PRRs, signaling molecules, transcription factors, inflammatory cytokines, cytokine receptors, endothelial and hemostasis molecules, and acute phase reactants [6]. Investigating the sorted gene list of significant variants, there was a prominent representation of genes encoding PRRs (*n* = 14), signaling molecules (*n* = 5), transcription factors (*n* = 3), cytokines (*n* = 18), and other immune-related molecules (*n* = 24). The elicited inflammatory response depends on the type of antigenic site on the pathogens that are generating the stimulus. Intracellular events are triggered by the binding of pathogens to the PPRs of immune cells, which further leads to the production of large amounts of inflammatory mediators [30, 31]. A high percentage of sepsis patients develop multiple organ dysfunction syndrome (MODS); the death rate is significantly higher among these patients [32]. This phenomenon is due to the infection, but the overlapping inflammatory immune events are also responsible. The inflammatory responses generated differ based on the type of tissue function and the receptors that trigger the reactions responsible for the biosynthesis of the mediators [33, 34]. Identifying patients at risk of developing sepsis may improve their outcomes by enabling the use of targeted treatments such as antibiotic prophylaxis, substitution therapy, or plasma transfusions.

In this meta-analysis, using the Venice criteria results, we identified four variants with strong (TLR1 rs5743551-7202A/G, RAGE rs1800625-429 T/C and rs1800624-374 T/A, and miR-608 rs4919510G/C) and 14 variants with moderate levels of cumulative epidemiological evidence of associations with the risk of sepsis. The remaining 11 variants had weak evidence; many of these were identified through meta-analyses using dominant/recessive models or ethnicity/age-specific subgroup analyses. In summary, we provide an updated and more systematic summary of the available evidence compared with the existing studies in this field [35, 36]. Well-designed studies with large sample sizes are essential to clarify the association of these significant variants with the susceptibility to sepsis.

Our meta-analysis also provides no evidence of an association with the risk of sepsis for 313 of the 405 variants evaluated in our study, supporting the idea that the vast majority of genetic variants evaluated in candidate genetic association studies may not be truly related to sepsis. Methodological limitations in previous candidate genetic studies, such as small sample sizes or inappropriate controls, may explain some of the null associations. However, of the 313 nonsignificant variants, 15 variants showed null associations with the risk of sepsis in meta-analyses including a minimum of 1000 cases and 1000 controls, which provides approximately 80% power to detect an OR of 1.15 under the additive model for a variant with MAF 0.10 and type 1 error 0.05. Thus, future epidemiological studies with similar sample sizes are unlikely to be helpful in assessing the effects of these variants.

To the best of our knowledge, this study is the largest and most comprehensive assessment of the literature regarding genetic association studies investigating the risk of sepsis that has been conducted to date. However, this study still had some limitations. First, some non-English articles, unpublished reports, and studies without sufficient data were not included in the study, which may have biased the results. Second, the number of eligible studies included in this meta-analysis was small, and the sample size of each study was relatively small, especially in the subgroup analyses, which might have influenced the statistical power. Moreover, we did not have precise information about the ethnicities of all the included populations; our classifications were broad and prone to the effects of population stratification. Third, as none of the studies included in this analysis considered the effect of gene-gene/environment interactions on the pathogenesis of sepsis, this issue could not be addressed in our study. Fourth, the overall outcome was based on unadjusted data; a more precise analysis stratified by variables such as gender, age, and type of infection could not be performed due to the limitations of the data, which also restricted our ability to detect possible sources of heterogeneity. Finally, despite the systematically internationally recognized approaches applied in this study, there is still room for future improvement in assessing the credibility of these associations. The Venice criteria improve the consistency and objectiveness of the interpretation and reporting of genetic associations, but this evidence does not determine causality. Additional sources of evidence, such as gene knockout experiments, gene expression microarray experiments, or other mechanistic data, are necessary to understand the precise functions of variants or genes. In general, associations with strong credibility deserve in-depth evaluations including biological investigations; moderately credible associations warrant more genetic and biological studies; and weak genetic associations may be not worthy of further investigation unless strong mechanistic evidence has been demonstrated.

## Conclusions

In summary, we performed the first systematic review and meta-analysis of the evidence linking genetic variants to the susceptibility to sepsis. Several genetic variants were confirmed to be significantly associated with the risk of sepsis. We hope that this unprecedented collection of data might represent a useful platform for investigators involved in this research field.

## Additional files

Additional file 1: Search strategy. Venice criteria. The list of included articles. (DOCX 70 kb)

Additional file 2: **Table S1.** Main characteristics of the studies included. (XLS 354 kb)

Additional file 3: **Table S2.** Characteristics of the genetic variants investigated for associations with the risk of sepsis. (DOCX 70 kb)

Additional file 4: **Figure S1.** Number of publications (*Y*-axis) per year (*X*-axis) addressing the association of genetic variants with the risk of sepsis. In 2018 (diamond), the literature search was performed until December. (TIF 5633 kb)

Additional file 5: **Figure S2.** Chromosome distribution of the genetic variants tested for their association with the risk of sepsis. mtRNA: mitochondrial DNA. (TIF 5967 kb)

Additional file 6: **Table S3.** Genetic variants associated with the risk of sepsis in the meta-analyses of all available data. (XLS 125 kb)

Additional file 7: **Table S4.** Genetic associations with sepsis risk investigated in fewer than three studies. (DOCX 39 kb)

## Abbreviations

CI: Confidence interval
DNA: Deoxyribonucleic acid
GWAS: Genome-wide association study
HWE: Hardy-Weinberg equilibrium
Ins/Del: Insertion/deletions
MAF: Minor allele frequency
MODS: Multiple organ dysfunction syndrome
OR: Odds ratio
PCR-RFLP: Polymerase chain reaction-restriction fragment length polymorphism
PRR: Pattern recognition receptors
RR: Relative risk
SNP: Single nucleotide polymorphisms
UTR: Untranslated region
VNTR: Variable number of tandem repeats
