# Supplementary material for: Host genetic variants in sepsis risk: a field synopsis and meta-analysis
Source: Crit Care. 2019 Jan 25;23:26. doi: 10.1186/s13054-019-2313-0 (PMC6347778; doi:10.1186/s13054-019-2313-0)
Supplement: Supplementary file 1 — Search strategy. Venice criteria. The list of included articles. (DOCX 70 kb) [file 13054_2019_2313_MOESM1_ESM.docx]

**Supplemental materials**

**1. Search strategy**

The following strategy was used in **PubMed**:

Search ((((polymorphism) OR variant) OR mutation)) AND ((((sepsis) OR severe sepsis) OR septic shock) OR septicemia) in All Fields.

The following strategy was used in **Medline**:

Search (polymorphism or variant or mutation) and (sepsis or severe sepsis or septic shock or septicemia) in Anywhere.

The following strategy was used in **Embase**:

#1 ‘sepsis’/exp OR sepsis OR severe AND (‘sepsis’/exp OR sepsis) OR septic AND (‘shock’/exp OR shock) OR ‘septicemia’/exp OR septicemia

#2 polymorphism OR variant OR ‘mutation’/exp OR mutation

#3 #1 AND #2

The following strategy was used in **Web of Knowledge**:

Search (polymorphism or variant or mutation) and (sepsis or severe sepsis or septic shock or septicemia) in Topic

The following strategy was used in **HuGE Navigator**:

Search term (sepsis [Text + Mesh])

**2. Venice criteria**

**2.1 Amount of evidence**

A: Large-scale evidence — risk genetic group (alleles or genotypes) in cases and controls>1,000.

B: Moderate amount of evidence — risk genetic group in cases and controls between 100 and 1,000.

C: Little evidence — risk genetic group in cases and controls<100.

**2.2 Replication of association**

A: Little between-study heterogeneity — *I*^2^<25%.

B: Moderate between-study heterogeneity — *I*^2^ between 25% and 50%.

C: Large between-study heterogeneity — *I*^2^>50%.

**2.3 Protection from bias**

A: No observable bias and bias was unlikely to explain the presence of the association.

B: Bias was potentially present.

C: Bias is demonstrable or is likely to explain the presence of the association.

**2.4 General checks for bias**

1) Association lost with exclusion of first study;

2) Association lost with exclusion of studies deviated from HWE;

3) Small magnitude of association (i.e., 0.87<OR<1.15);

4) Evidence of small-study effect (p<0.10 in Egger test).

**3. The lists of included articles**

1. Zhang A, Gu W, Lu H, Zeng L, Zhang L, Du D, Hao J, Wen D, Wang X, Jiang J: **Genetic contribution of suppressor of cytokine signalling polymorphisms to the susceptibility to infection after traumatic injury**. *Clin Exp Immunol* 2018, **194**(1):93-102.
2. Wang X, Zhang AQ, Gu W, Wen DL, Lu HX, Yang JH, Wang X, Deng J, Zhang HQ, Du DY *et al*: **Clinical relevance of single nucleotide polymorphisms in the CXCL1 and CXCL12 genes in patients with major trauma**. *J Trauma Acute Care Surg* 2018 (Accepted).
3. Tayel SI, Soliman SE, Elsayed HM: **Vitamin D deficiency and vitamin D receptor variants in mothers and their neonates are risk factors for neonatal sepsis**. *Steroids* 2018, **134**:37-42.
4. Qiu P, Wang L, Ni J, Zhang Y: **Associations between HMGB1 gene polymorphisms and susceptibility and clinical outcomes in Chinese Han sepsis patients**. *Gene* 2018, **687**:23-29.
5. Martin SL, Desai S, Nanavati R, Colah RB, Ghosh K, Mukherjee MB: **Innate immune gene polymorphisms and their association with neonatal sepsis**. *Infect Genet Evol* 2018, **62**:205-210.
6. Liu ZL, Hu J, Xiao XF, Peng Y, Zhao SP, Xiao XZ, Yang MS: **The CD40 rs1883832 Polymorphism Affects Sepsis Susceptibility and sCD40L Levels**. *BioMed research international* 2018, **2018**:7497314.
7. Liu Y, Wan W, Fang F, Guo L, Zhao Y, Zhang X, Huang F: **Clinical relevance of peroxisome proliferator-activated receptor-gamma gene polymorphisms with sepsis**. *Journal of clinical laboratory analysis* 2018, **32**(4):e22340.
8. Liu C, Jin P, Luo Y, Xu J, Kong C, Chen J, Xie H, Zhou G: **Association of Single-Nucleotide Polymorphisms of C-Reactive Protein Gene with Susceptibility to Infantile Sepsis in Southern China**. *Med Sci Monit* 2018, **24**:590-595.
9. He J, Zhang Q, Zhang W, Chen F, Zhao T, Lin Y, Li J, Liu Y, Liu Y, Shao Y: **The interleukin-27 -964A>G polymorphism enhances sepsis-induced inflammatory responses and confers susceptibility to the development of sepsis**. *Crit Care* 2018, **22**(1):248.
10. Fu Y, Zhang J, Bai H, Chen Y, Liu R, Bai N: **Genetic association between CD86 polymorphisms and the risk of sepsis in a Chinese Han population**. *Hum Immunol* 2018, **79**(11):817-820.
11. Acar L, Atalan N, Karagedik EH, Ergen A: **Tumour Necrosis Factor-alpha and Nuclear Factor-kappa B Gene Variants in Sepsis**. *Balkan medical journal* 2018, **35**(1):30-35.
12. Zhang H, Lu Y, Sun G, Teng F, Luo N, Jiang J, Wen A: **The common promoter polymorphism rs11666254 downregulates FPR2/ALX expression and increases risk of sepsis in patients with severe trauma**. *Crit Care* 2017, **21**(1):171.
13. Zeljic K, Elkilany A, Supic G, Surbatovic M, Djordjevic D, Magic Z, Bozic B: **Vitamin D receptor gene polymorphisms association with the risk of sepsis and mortality**. *Int J Immunogenet* 2017, **44**(3):129-134.
14. Yu L, Yang H, Xiang Y, Guo X, Liu Z, Guo R: **Association between Cx37 rs1764390 polymorphism and susceptibility to sepsis in Chinese population**. *Infect Genet Evol* 2017, **48**:64-70.
15. Shao Y, Shao X, He J, Cai Y, Zhao J, Chen F, Tao H, Yin Z, Tan X, He Y *et al*: **The promoter polymorphisms of receptor for advanced glycation end products were associated with the susceptibility and progression of sepsis**. *Clin Genet* 2017, **91**(4):564-575.
16. Shao Y, Chen F, Chen Y, Zhang W, Lin Y, Cai Y, Yin Z, Tao S, Liao Q, Zhao J *et al*: **Association between genetic polymorphisms in the autophagy-related 5 gene promoter and the risk of sepsis**. *Scientific reports* 2017, **7**(1):9399.
17. Seaton ME, Parent BA, Sood RF, Wurfel MM, Muffley LA, O'Keefe GE, Gibran NS: **Melanocortin-1 Receptor Polymorphisms and the Risk of Complicated Sepsis After Trauma: A Candidate Gene Association Study**. *Shock* 2017, **47**(1):79-85.
18. Miao T, Pu Y, Zhou B, Chen P, Wang Y, Song Y, Zhao J, Zhang L: **Association between polymorphisms in IL21 gene and risk for sepsis**. *Biomarkers : biochemical indicators of exposure, response, and susceptibility to chemicals* 2017, **22**(1):14-18.
19. Mao ZR, Zhang SL, Feng B: **Association of IL-10 (-819T/C, -592A/C and -1082A/G) and IL-6 -174G/C gene polymorphism and the risk of pneumonia-induced sepsis**. *Biomarkers : biochemical indicators of exposure, response, and susceptibility to chemicals* 2017, **22**(2):106-112.
20. Liang Y, Huang X, Jiang Y, Qin Y, Peng D, Huang Y, Li J, Sooranna SR, Pinhu L: **Endothelial protein C receptor polymorphisms and risk of sepsis in a Chinese population**. *J Int Med Res* 2017, **45**(2):504-513.
21. Jimenez-Sousa MA, Medrano LM, Liu P, Fernandez-Rodriguez A, Almansa R, Gomez-Sanchez E, Ortega A, Heredia-Rodriguez M, Gomez-Pesquera E, Tamayo E *et al*: **IL-6 rs1800795 polymorphism is associated with septic shock-related death in patients who underwent major surgery: a preliminary retrospective study**. *Ann Intensive Care* 2017, **7**(1):22.
22. Jimenez-Sousa MA, Medrano LM, Liu P, Almansa R, Fernandez-Rodriguez A, Gomez-Sanchez E, Rico L, Heredia-Rodriguez M, Gomez-Pesquera E, Tamayo E *et al*: **IL-1B rs16944 polymorphism is related to septic shock and death**. *European journal of clinical investigation* 2017, **47**(1):53-62.
23. He J, Chen Y, Lin Y, Zhang W, Cai Y, Chen F, Liao Q, Yin Z, Wang Y, Tao S *et al*: **Association study of MCP-1 promoter polymorphisms with the susceptibility and progression of sepsis**. *PLoS One* 2017, **12**(5):e0176781.
24. Hahn EC, Zambra FMB, Kamada AJ, Delongui F, Grion CMC, Reiche EMV, Chies JAB: **Association of HLA-G 3'UTR polymorphisms and haplotypes with severe sepsis in a Brazilian population**. *Hum Immunol* 2017, **78**(11-12):718-723.
25. Dou XM, Cheng HJ, Meng L, Zhou LL, Ke YH, Liu LP, Li YM: **Correlations between ACE single nucleotide polymorphisms and prognosis of patients with septic shock**. *Bioscience reports* 2017, **37**(2).
26. Shao Y, He J, Chen F, Cai Y, Zhao J, Lin Y, Yin Z, Tao H, Shao X, Huang P *et al*: **Association Study Between Promoter Polymorphisms of ADAM17 and Progression of Sepsis**. *Cellular physiology and biochemistry : international journal of experimental cellular physiology, biochemistry, and pharmacology* 2016, **39**(4):1247-1261.
27. Montoya-Ruiz C, Jaimes FA, Rugeles MT, Lopez JA, Bedoya G, Velilla PA: **Variants in LTA, TNF, IL1B and IL10 genes associated with the clinical course of sepsis**. *Immunol Res* 2016, **64**(5-6):1168-1178.
28. Hu D, Wang H, Huang X, Jiang Y, Qin Y, Xiong B, Qin G, Sooranna SR, Pinhu L: **Investigation of association between IL-8 serum levels and IL8 polymorphisms in Chinese patients with sepsis**. *Gene* 2016, **594**(1):165-170.
29. Gokay SS, Yildizdas RD, Yilmaz M, Aksoy K, Yalin AE, Sertdemir Y, Ucar G, Horoz OO, Ozduran FD, Yilmaz HL: **MyD 88 Polymorphisms in Children Diagnosed with Sepsis**. *Balkan medical journal* 2016, **33**(6):633-638.
30. Gao JW, Zeng L, Zhang AQ, Wang X, Pan W, Du DY, Zhang LY, Gu W, Jiang JX: **Identification of Haplotype Tag Single-Nucleotide Polymorphisms within the PPAR Family Genes and Their Clinical Relevance in Patients with Major Trauma**. *International journal of environmental research and public health* 2016, **13**(4):374.
31. Das B, Patra S, Behera C, Suar M: **Genotyping of vitamin D receptor gene polymorphisms using mismatched amplification mutation assay in neonatal sepsis patients of Odisha, eastern India**. *Infect Genet Evol* 2016, **45**:40-47.
32. Azab SF, Abdalhady MA, Elsaadany HF, Elkomi MA, Elhindawy EM, Sarhan DT, Salam MM, Allah MA, Emam AA, Noah MA *et al*: **Interleukin-10 -1082 G/A gene polymorphisms in Egyptian children with CAP: A case-control study**. *Medicine (Baltimore)* 2016, **95**(26):e4013.
33. Atalan N, Karagedik H, Acar L, Isbir S, Yilmaz SG, Ergen A, Isbir T: **Analysis of Toll-like Receptor 9 Gene Polymorphisms in Sepsis**. *In Vivo* 2016, **30**(5):639-643.
34. Toubiana J, Courtine E, Tores F, Asfar P, Daubin C, Rousseau C, Ouaaz F, Marin N, Cariou A, Chiche JD *et al*: **Association of REL polymorphisms and outcome of patients with septic shock**. *Ann Intensive Care* 2016, **6**(1):28.
35. Thair SA, Topchiy E, Boyd JH, Cirstea M, Wang C, Nakada TA, Fjell CD, Wurfel M, Russell JA, Walley KR: **TNFAIP2 Inhibits Early TNFalpha-Induced NF-x03BA;B Signaling and Decreases Survival in Septic Shock Patients**. *J Innate Immun* 2016, **8**(1):57-66.
36. Swierzko AS, Szala-Pozdziej A, Kilpatrick DC, Sobocinski M, Chojnacka K, Sokolowska A, Michalski M, Mazerant K, Jensenius JC, Matsushita M *et al*: **Components of the lectin pathway of complement activation in paediatric patients of intensive care units**. *Immunobiology* 2016, **221**(5):657-669.
37. Georgitsi MD, Vitoros V, Panou C, Tsangaris I, Aimoniotou E, Gatselis NK, Chasou E, Kouliatsis G, Leventogiannis K, Velissaris D *et al*: **Individualized significance of the -251 A/T single nucleotide polymorphism of interleukin-8 in severe infections**. *Eur J Clin Microbiol Infect Dis* 2016, **35**(4):563-570.
38. Gao JW, Zeng L, Zhang AQ, Wang X, Pan W, Du DY, Zhang LY, Gu W, Jiang JX: **Identification of Haplotype Tag Single-Nucleotide Polymorphisms within the PPAR Family Genes and Their Clinical Relevance in Patients with Major Trauma**. *Int J Environ Res Public Health* 2016, **13**(4):374. doi: 310.3390/ijerph13040374.
39. Fakhri D, Djauzi S, Murni TW, Rachmat J, Harahap AR, Rahayuningsih SE, Mansyur M, Santoso A: **Genetic polymorphism in postoperative sepsis after open heart surgery in infants**. *Asian cardiovascular & thoracic annals* 2016, **24**(4):326-331.
40. Esposito S, Bosis S, Orenti A, Spena S, Montinaro V, Bianchini S, Zampiero A, Principi N: **Genetic polymorphisms and the development of invasive bacterial infections in children**. *International journal of immunopathology and pharmacology* 2016, **29**(1):99-104.
41. Beppler J, Koehler-Santos P, Pasqualim G, Matte U, Alho CS, Dias FS, Kowalski TW, Velasco IT, Monteiro RC, Pinheiro da Silva F: **Fc Gamma Receptor IIA (CD32A) R131 Polymorphism as a Marker of Genetic Susceptibility to Sepsis**. *Inflammation* 2016, **39**(2):518-525.
42. Zhang AQ, Gu W, Zeng L, Zhang LY, Du DY, Zhang M, Hao J, Yue CL, Jiang J: **Genetic variants of microRNA sequences and susceptibility to sepsis in patients with major blunt trauma**. *Ann Surg* 2015, **261**(1):189-196. doi: 110.1097/SLA.0000000000000687.
43. Zeng L, Du J, Gu W, Zhang AQ, Wang HY, Wen DL, Qiu L, Yang XT, Sun JH, Zhang M *et al*: **Rs1800625 in the receptor for advanced glycation end products gene predisposes to sepsis and multiple organ dysfunction syndrome in patients with major trauma**. *Crit Care* 2015, **19**:6.
44. West SD, Ziegler A, Brooks T, Krencicki M, Myers O, Mold C: **An FcgammaRIIa polymorphism with decreased C-reactive protein binding is associated with sepsis and decreased monocyte HLA-DR expression in trauma patients**. *J Trauma Acute Care Surg* 2015, **79**(5):773-781.
45. Wang XL, Zhang L, Li YW, Hou HM, Sun HB: **[Association between toll-like receptors 2 and 5 polymorphisms and neonatal sepsis]**. *Zhongguo dang dai er ke za zhi = Chinese journal of contemporary pediatrics* 2015, **17**(12):1316-1321.
46. Wang C, Gui Q, Zhang K: **Functional polymorphisms in CD86 gene are associated with susceptibility to pneumonia-induced sepsis**. *Apmis* 2015, **123**(5):433-438.
47. van der Starre WE, van Nieuwkoop C, Thomson U, Zijderveld-Voshart MS, Koopman JP, van der Reijden TJ, van Dissel JT, van de Vosse E: **Urinary proteins, vitamin D and genetic polymorphisms as risk factors for febrile urinary tract infection and relation with bacteremia: a case control study**. *PLoS One* 2015, **10**(3):e0121302.
48. Sun W, Li FS, Zhang YH, Wang XP, Wang CR: **Association of susceptibility to septic shock with platelet endothelial cell adhesion molecule-1 gene Leu125Val polymorphism and serum sPECAM-1 levels in sepsis patients**. *International journal of clinical and experimental medicine* 2015, **8**(11):20490-20498.
49. Song H, Tang L, Xu M, Li H, Xu S, Li G, Bao X, Sun B, Cheng T, Yang Q *et al*: **CD86 polymorphism affects pneumonia-induced sepsis by decreasing gene expression in monocytes**. *Inflammation* 2015, **38**(2):879-885.
50. Schnetzke U, Spies-Weisshart B, Yomade O, Fischer M, Rachow T, Schrenk K, Glaser A, von Lilienfeld-Toal M, Hochhaus A, Scholl S: **Polymorphisms of Toll-like receptors (TLR2 and TLR4) are associated with the risk of infectious complications in acute myeloid leukemia**. *Genes Immun* 2015, **16**(1):83-88.
51. Rautanen A, Mills TC, Gordon AC, Hutton P, Steffens M, Nuamah R, Chiche JD, Parks T, Chapman SJ, Davenport EE *et al*: **Genome-wide association study of survival from sepsis due to pneumonia: an observational cohort study**. *Lancet Respir Med* 2015, **3**(1):53-60.
52. Ramakers BP, Giamarellos-Bourboulis EJ, Tasioudis C, Coenen MJ, Kox M, Vermeulen SH, Groothuismink JM, van der Hoeven JG, Routsi C, Savva A *et al*: **Effects of the 34C>T Variant of the AMPD1 Gene on Immune Function, Multi-Organ Dysfunction, and Mortality in Sepsis Patients**. *Shock* 2015, **44**(6):542-547.
53. Peng LS, Li J, Zhou GS, Deng LH, Yao HG: **Relationships between genetic polymorphisms of triggering receptor expressed on myeloid cells-1 and septic shock in a Chinese Han population**. *World J Emerg Med* 2015, **6**(2):123-130.
54. Panayides A, Ioakeimidou A, Karamouzos V, Antonakos N, Koutelidakis I, Giannikopoulos G, Makaritsis K, Voloudakis N, Toutouzas K, Rovina N *et al*: **-572 G/C single nucleotide polymorphism of interleukin-6 and sepsis predisposition in chronic renal disease**. *Eur J Clin Microbiol Infect Dis* 2015, **34**(12):2439-2446.
55. Pan W, Zhang AQ, Gu W, Gao JW, Du DY, Zhang LY, Zeng L, Du J, Wang HY, Jiang JX: **Identification of haplotype tag single nucleotide polymorphisms within the nuclear factor-kappaB family genes and their clinical relevance in patients with major trauma**. *Crit Care* 2015, **19**:95.
56. Nakada TA, Russell JA, Boyd JH, Thair SA, Walley KR: **Identification of a nonsynonymous polymorphism in the SVEP1 gene associated with altered clinical outcomes in septic shock**. *Crit Care Med* 2015, **43**(1):101-108.
57. Nakada TA, Boyd JH, Russell JA, Aguirre-Hernandez R, Wilkinson MD, Thair SA, Nakada E, McConechy MK, Fjell CD, Walley KR: **VPS13D Gene Variant Is Associated with Altered IL-6 Production and Mortality in Septic Shock**. *J Innate Immun* 2015, **7**(5):545-553.
58. Mills TC, Chapman S, Hutton P, Gordon AC, Bion J, Chiche JD, Holloway PA, Stuber F, Garrard CS, Hinds CJ *et al*: **Variants in the Mannose-binding Lectin Gene MBL2 do not Associate With Sepsis Susceptibility or Survival in a Large European Cohort**. *Clin Infect Dis* 2015, **61**(5):695-703.
59. Meawed TE, Mansour MA, Mansour SA, Mohamed ML, Ibrahim EM, Ali AM: **Functional and prognostic relevance of -173 G/C gene polymorphism of macrophage migration inhibitory factor in sepsis patients in Egyptian intensive care units**. *Eastern Mediterranean health journal = La revue de sante de la Mediterranee orientale = al-Majallah al-sihhiyah li-sharq al-mutawassit* 2015, **21**(10):762-769.
60. Mansur A, Liese B, Steinau M, Ghadimi M, Bergmann I, Tzvetkov M, Popov AF, Beissbarth T, Bauer M, Hinz J: **The CD14 rs2569190 TT Genotype Is Associated with an Improved 30-Day Survival in Patients with Sepsis: A Prospective Observational Cohort Study**. *PLoS One* 2015, **10**(5):e0127761.
61. Majolo F, Oliveira Paludo FJ, Ponzoni A, Graebin P, Dias FS, Alho CS: **Effect of 593C>T GPx1 SNP alone and in synergy with 47C>T SOD2 SNP on the outcome of critically ill patients**. *Cytokine* 2015, **71**(2):312-317.
62. Lorente L, Martin MM, Borreguero-Leon JM, Barrios Y, Sole-Violan J, Ferreres J, Labarta L, Diaz C, Jimenez A: **The 4G/4G Genotype of PAI-1 Polymorphism Is Associated with Higher Plasma PAI-1 Concentrations and Mortality in Patients with Severe Sepsis**. *PLoS One* 2015, **10**(6):e0129565.
63. Liu L, Ning B: **The role of MBL2 gene polymorphism in sepsis incidence**. *International journal of clinical and experimental pathology* 2015, **8**(11):15123-15127.
64. Kompoti M, Michopoulos A, Michalia M, Clouva-Molyvdas PM, Germenis AE, Speletas M: **Genetic polymorphisms of innate and adaptive immunity as predictors of outcome in critically ill patients**. *Immunobiology* 2015, **220**(3):414-421.
65. Gupta DL, Nagar PK, Kamal VK, Bhoi S, Rao DN: **Clinical relevance of single nucleotide polymorphisms within the 13 cytokine genes in North Indian trauma hemorrhagic shock patients**. *Scandinavian journal of trauma, resuscitation and emergency medicine* 2015, **23**:96.
66. Grube M, Brenmoehl J, Rogler G, Hahn J, Herr W, Holler E: **Donor Nucleotide-Binding Oligomerization-Containing Protein 2 (NOD2) Single Nucleotide Polymorphism 13 Is Associated with Septic Shock after Allogeneic Stem Cell Transplantation**. *Biol Blood Marrow Transplant* 2015, **21**(8):1399-1404.
67. Feng B, Mao ZR, Pang K, Zhang SL, Li L: **Association of tumor necrosis factor alpha -308G/A and interleukin-6 -174G/C gene polymorphism with pneumonia-induced sepsis**. *J Crit Care* 2015, **30**(5):920-923.
68. Demmert M, Schaper A, Pagel J, Gebauer C, Emeis M, Heitmann F, Kribs A, Siegel J, Muller D, Keller-Wackerbauer A *et al*: **FUT 2 polymorphism and outcome in very-low-birth-weight infants**. *Pediatr Res* 2015, **77**(4):586-590.
69. Cui L, Gao Y, Xie Y, Wang Y, Cai Y, Shao X, Ma X, Li Y, Ma G, Liu G *et al*: **An ADAM10 promoter polymorphism is a functional variant in severe sepsis patients and confers susceptibility to the development of sepsis**. *Crit Care* 2015, **19**:73.
70. Chi YF, Chai JK, Yu YM, Luo HM, Zhang QX, Feng R: **Association between PAI-1 polymorphisms and plasma PAI-1 level with sepsis in severely burned patients**. *Genetics and molecular research : GMR* 2015, **14**(3):10081-10086.
71. Belopolskaya OB, Smelaya TV, Moroz VV, Golubev AM, Salnikova LE: **Clinical associations of host genetic variations in the genes of cytokines in critically ill patients**. *Clin Exp Immunol* 2015, **180**(3):531-541.
72. Allam G, Alsulaimani AA, Alzaharani AK, Nasr A: **Neonatal infections in Saudi Arabia: Association with cytokine gene polymorphisms**. *Central-European journal of immunology / Polish Society for Immunology and eleven other Central-European immunological societies* 2015, **40**(1):68-77.
73. Zidan HE, Elbehedy RM, Azab SF: **IL6-174 G/C gene polymorphism and its relation to serum IL6 in Egyptian children with community-acquired pneumonia**. *Cytokine* 2014, **67**(2):60-64.
74. Yousef AA, Suliman GA, Mabrouk MM: **The Value of Admission Serum IL-8 Monitoring and the Correlation with IL-8 (-251A/T) Polymorphism in Critically Ill Patients**. *ISRN inflammation* 2014, **2014**:494985.
75. Wang H, Wei Y, Zeng Y, Qin Y, Xiong B, Qin G, Li J, Hu D, Qiu X, Sooranna SR *et al*: **The association of polymorphisms of TLR4 and CD14 genes with susceptibility to sepsis in a Chinese population**. *BMC Med Genet* 2014, **15**:123.
76. Wang D, Zhong X, Huang D, Chen R, Bai G, Li Q, Yu B, Fan Y, Sun X: **Functional polymorphisms of interferon-gamma affect pneumonia-induced sepsis**. *PLoS One* 2014, **9**(1):e87049.
77. Thompson CM, Holden TD, Rona G, Laxmanan B, Black RA, O'Keefe GE, Wurfel MM: **Toll-like receptor 1 polymorphisms and associated outcomes in sepsis after traumatic injury: a candidate gene association study**. *Ann Surg* 2014, **259**(1):179-185.
78. Telleria-Orriols JJ, Garcia-Salido A, Varillas D, Serrano-Gonzalez A, Casado-Flores J: **TLR2-TLR4/CD14 polymorphisms and predisposition to severe invasive infections by Neisseria meningitidis and Streptococcus pneumoniae**. *Medicina Intensiva* 2014, **38**(6):356-362.
79. Shi D, Song Z, Yin J, Xue M, Yao C, Sun Z, Shao M, Deng Z, Zhang Y, Tao Z *et al*: **Genetic variation in the tissue factor gene is associated with clinical outcome in severe sepsis patients**. *Crit Care* 2014, **18**(6):631.
80. Shao Y, Li J, Cai Y, Xie Y, Ma G, Li Y, Chen Y, Liu G, Zhao B, Cui L *et al*: **The functional polymorphisms of miR-146a are associated with susceptibility to severe sepsis in the Chinese population**. *Mediators Inflamm* 2014, **2014**:916202.
81. Savva A, Plantinga TS, Kotanidou A, Farcas M, Baziaka F, Raftogiannis M, Orfanos SE, Dimopoulos G, Netea MG, Giamarellos-Bourboulis EJ: **Association of autophagy-related 16-like 1 (ATG16L1) gene polymorphism with sepsis severity in patients with sepsis and ventilator-associated pneumonia**. *Eur J Clin Microbiol Infect Dis* 2014, **33**(9):1609-1614.
82. Ramakrishna K, Pugazhendhi S, Kabeerdoss J, Peter JV: **Association between heat shock protein 70 gene polymorphisms and clinical outcomes in intensive care unit patients with sepsis**. *Indian journal of critical care medicine : peer-reviewed, official publication of Indian Society of Critical Care Medicine* 2014, **18**(4):205-211.
83. Nelson CL, Pelak K, Podgoreanu MV, Ahn SH, Scott WK, Allen AS, Cowell LG, Rude TH, Zhang Y, Tong A *et al*: **A genome-wide association study of variants associated with acquisition of Staphylococcus aureus bacteremia in a healthcare setting**. *BMC Infectious Diseases* 2014, **14**:83.
84. Nachtigall I, Tamarkin A, Tafelski S, Weimann A, Rothbart A, Heim S, Wernecke KD, Spies C: **Polymorphisms of the toll-like receptor 2 and 4 genes are associated with faster progression and a more severe course of sepsis in critically ill patients**. *J Int Med Res* 2014, **42**(1):93-110.
85. Meyer NJ, Ferguson JF, Feng R, Wang F, Patel PN, Li M, Xue C, Qu L, Liu Y, Boyd JH *et al*: **A functional synonymous coding variant in the IL1RN gene is associated with survival in septic shock**. *Am J Respir Crit Care Med* 2014, **190**(6):656-664.
86. Martin G, Asensi V, Montes AH, Collazos J, Alvarez V, Perez-Is L, Carton JA, Taboada F, Valle-Garay E: **Endothelial (NOS3 E298D) and inducible (NOS2 exon 22) nitric oxide synthase polymorphisms, as well as plasma NOx, influence sepsis development**. *Nitric oxide : biology and chemistry / official journal of the Nitric Oxide Society* 2014, **42**:79-86.
87. Martin G, Asensi V, Montes AH, Collazos J, Alvarez V, Carton JA, Taboada F, Valle-Garay E: **Role of plasma matrix-metalloproteases (MMPs) and their polymorphisms (SNPs) in sepsis development and outcome in ICU patients**. *Scientific reports* 2014, **4**:5002.
88. Mansur A, von Gruben L, Popov AF, Steinau M, Bergmann I, Ross D, Ghadimi M, Beissbarth T, Bauer M, Hinz J: **The regulatory toll-like receptor 4 genetic polymorphism rs11536889 is associated with renal, coagulation and hepatic organ failure in sepsis patients**. *J Transl Med* 2014, **12**:177.
89. Mansur A, Hinz J, Hillebrecht B, Bergmann I, Popov AF, Ghadimi M, Bauer M, Beissbarth T, Mihm S: **Ninety-Day Survival Rate of Patients With Sepsis Relates to Programmed Cell Death 1 Genetic Polymorphism rs11568821**. *J Investig Med* 2014, **62**(3):638-643.
90. Ma G, Wang H, Mo G, Cui L, Li Y, Shao Y, Liu X, Xie Y, Li J, Fu J *et al*: **The Pro12Ala Polymorphism of PPAR-gamma Gene Is Associated with Sepsis Disease Severity and Outcome in Chinese Han Population**. *PPAR research* 2014, **2014**:701971.
91. Kumar V, Cheng SC, Johnson MD, Smeekens SP, Wojtowicz A, Giamarellos-Bourboulis E, Karjalainen J, Franke L, Withoff S, Plantinga TS *et al*: **Immunochip SNP array identifies novel genetic variants conferring susceptibility to candidaemia**. *Nature communications* 2014, **5**:4675.
92. Kimura T, Watanabe E, Sakamoto T, Takasu O, Ikeda T, Ikeda K, Kotani J, Kitamura N, Sadahiro T, Tateishi Y *et al*: **Autophagy-related IRGM polymorphism is associated with mortality of patients with severe sepsis**. *PLoS One* 2014, **9**(3):e91522.
93. Jaskula E, Lange A, Kyrcz-Krzemien S, Markiewicz M, Dzierzak-Mietla M, Jedrzejczak WW, Czajka P, Mordak-Domagala M, Lange J, Gronkowska A *et al*: **NOD2/CARD15 Single Nucleotide Polymorphism 13 (3020insC) is Associated with Risk of Sepsis and Single Nucleotide Polymorphism 8 (2104C>T) with Herpes Viruses Reactivation in Patients after Allogeneic Hematopoietic Stem Cell Transplantation**. *Biol Blood Marrow Transplant* 2014, **20**(3):409-414.
94. Jabandziev P, Smerek M, Michalek J, Sr., Fedora M, Kosinova L, Hubacek JA, Michalek J, Jr.: **Multiple gene-to-gene interactions in children with sepsis: a combination of five gene variants predicts outcome of life-threatening sepsis**. *Crit Care* 2014, **18**(1):R1.
95. Hao J, He XD: **Haplotype analysis of ApoAI gene and sepsis-associated acute lung injury**. *Lipids in health and disease* 2014, **13**:79.
96. Esposito S, Zampiero A, Pugni L, Tabano S, Pelucchi C, Ghirardi B, Terranova L, Miozzo M, Mosca F, Principi N: **Genetic polymorphisms and sepsis in premature neonates**. *PLoS One* 2014, **9**(7):e101248.
97. Costa NA, Gut AL, Pimentel JA, Cozzolino SM, Azevedo PS, Fernandes AA, Polegato BF, Tanni SE, Gaiolla RD, Zornoff LA *et al*: **Erythrocyte selenium concentration predicts intensive care unit and hospital mortality in patients with septic shock: a prospective observational study**. *Crit Care* 2014, **18**(3):R92.
98. Chen Q, Xue H, Chen M, Gao F, Xu J, Liu Q, Yang X, Zheng L, Chen H: **High serum trypsin levels and the -409 T/T genotype of PRSS1 gene are susceptible to neonatal sepsis**. *Inflammation* 2014, **37**(5):1751-1756.
99. Baghel K, Srivastava RN, Chandra A, Raj S, Goel SK, Pant AB, Agrawal J: **Tumor necrosis factor-beta Nco1 polymorphism and susceptibility to sepsis following major elective surgery**. *Surg Infect (Larchmt)* 2014, **15**(3):213-220.
100. Baghel K, Srivastava RN, Chandra A, Goel SK, Agrawal J, Kazmi HR, Raj S: **TNF-alpha, IL-6, and IL-8 cytokines and their association with TNF-alpha-308 G/A polymorphism and postoperative sepsis**. *Journal of gastrointestinal surgery : official journal of the Society for Surgery of the Alimentary Tract* 2014, **18**(8):1486-1494.
101. Asfaw Idosa B, Sahdo B, Balcha E, Kelly A, Soderquist B, Sarndahl E: **C10X polymorphism in the CARD8 gene is associated with bacteraemia**. *Immunity, inflammation and disease* 2014, **2**(1):13-20.
102. Zhao Y, Tao L, Jiang D, Chen X, Li P, Ning Y, Xiong R, Liu P, Peng Y, Zhou Y-G: **The -144C/A Polymorphism in the Promoter of HSP90beta Is Associated with Multiple Organ Dysfunction Scores**. *PloS one* 2013, **8**(3):e58646-e58646.
103. Zapata-Tarres M, Arredondo-Garcia JL, Rivera-Luna R, Klunder-Klunder M, Mancilla-Ramirez J, Sanchez-Urbina R, Vazquez-Cruz MY, Juarez-Villegas LE, Palomo-Colli MA: **Interleukin-1 receptor antagonist gene polymorphism increases susceptibility to septic shock in children with acute lymphoblastic leukemia**. *Pediatr Infect Dis J* 2013, **32**(2):136-139.
104. Wu XX, Wan QQ, Ye QF, Zhou JD: **Correlation of tumor necrosis factor-(beta) and interleukin-1 gene cluster polymorphism with susceptibility to bacteremia in patients undergoing kidney transplantation**. *Chinese Medical Journal* 2013, **126**(24):4603-4607.
105. Wan QQ, Ye QF, Zhou JD: **Mannose-binding lectin 2 and ficolin-2 gene polymorphisms influence the susceptibility to bloodstream infections in kidney transplant recipients**. *Transplantation Proceedings* 2013, **45**(9):3289-3292.
106. Wan QQ, Li JL, Ye QF, Zhou JD: **Genetic Association of Tumor Necrosis Factor-beta, Interleukin-10, and Interleukin-1 Gene Cluster Polymorphism With Susceptibility to Pneumonia in Kidney Transplant Recipients**. *Transplantation Proceedings* 2013, **45**(6):2211-2214.
107. Vazquez-Armenta G, Gonzalez-Leal N, la Torre MJV, Munoz-Valle JF, Ramos-Marquez ME, Hernandez-Canaveral I, Plascencia-Hernandez A, Siller-Lopez F: **Short (GT)n microsatellite repeats in the heme oxygenase-1 gene promoter are associated with antioxidant and anti-inflammatory status in Mexican pediatric patients with sepsis**. *Tohoku Journal of Experimental Medicine* 2013, **231**(3):201-209.
108. Vassiliou AG, Maniatis NA, Kotanidou A, Kallergi M, Karystinaki FS, Letsiou E, Glynos C, Kopterides P, Vassiliadi D, Nikitas N *et al*: **Endothelial protein C receptor polymorphisms and risk of severe sepsis in critically ill patients**. *Intensive Care Medicine* 2013, **39**(10):1752-1759.
109. Smeekens SP, Malireddi RK, Plantinga TS, Buffen K, Oosting M, Joosten LAB, Kullberg BJ, Perfect JR, Scott WK, van de Veerdonk FL *et al*: **Autophagy is redundant for the host defense against systemic Candida albicans infections**. *European Journal of Clinical Microbiology and Infectious Diseases* 2013:1-12.
110. Silva FP, Preuhs Filho G, Finger E, Barbeiro HV, Zampieri FG, Goulart AC, Torggler Filho F, Panajotopoulos N, Velasco IT, Kalil J *et al*: **HLA-A*31 as a marker of genetic susceptibility to sepsis**. *Rev Bras Ter Intensiva* 2013, **25**(4):284-289.
111. Sampath V, Mulrooney NP, Garland JS, He J, Patel AL, Cohen JD, Simpson PM, Hines RN: **Toll-like receptor genetic variants are associated with Gram-negative infections in VLBW infants**. *Journal of Perinatology* 2013, **33**(10):772-777.
112. Salnikova LE, Smelaya TV, Moroz VV, Golubev AM, Rubanovich AV: **Host genetic risk factors for community-acquired pneumonia**. *Gene* 2013, **518**(2):449-456.
113. Rodriguez-Osorio CA, Lima G, Herrera-Caceres JO, Villegas-Torres BE, Zuniga J, Ponce-De-Leon S, Llorente L, Sifuentes-Osornio J: **Genetic variations in toll-like receptor 4 in Mexican-Mestizo patients with intra-abdominal infection and/or pneumonia**. *Immunology Letters* 2013, **153**(1-2):41-46.
114. Paludo FJ, Picanco JB, Fallavena PR, Fraga Lda R, Graebin P, Nobrega Ode T, Dias FS, Alho CS: **Higher frequency of septic shock in septic patients with the 47C allele (rs4880) of the SOD2 gene**. *Gene* 2013, **517**(1):106-111.
115. Osthoff M, Yong HMA, Dean MM, Eisen DP: **Significance of Mannose-Binding Lectin Deficiency and Nucleotide-Binding Oligomerization Domain 2 Polymorphisms in Staphylococcus aureus Bloodstream Infections: A Case-Control Study**. *PLoS ONE* 2013, **8**(9).
116. Kothari N, Bogra J, Abbas H, Kohli M, Malik A, Kothari D, Srivastava S, Singh PK: **Tumor Necrosis Factor gene polymorphism results in high TNF level in sepsis and septic shock**. *Cytokine* 2013, **61**(2):676-681.
117. Garcia-Laorden M, Rodriguez de Castro F, Sole-Violan J, Payeras A, Luisa Briones M, Borderias L, Aspa J, Blanquer J, Rajas O, Alberto Marcos-Ramos J *et al*: **The role of mannose-binding lectin in pneumococcal infection**. *European Respiratory Journal* 2013, **41**(1):131-139.
118. Dong GH, Gong JP, Li JZ, Luo YH, Li ZD, Li PZ, He K: **Association Between Gene Polymorphisms of IRAK-M and the Susceptibility of Sepsis**. *Inflammation* 2013, **36**(5):1087-1093.
119. De Mare-Bredemeijer E, Bartakova R, Frankova S, Roelen D, Visseren T, Trunecka P, Metselaar H, Jirsa M, Kwekkeboom J, Sperl J: **The TNF-alpha -238 G-allele predisposes to severe bacterial infection in patients with end-stage liver disease enlisted for liver transplantation**. *Liver Transplantation* 2013, **19**(6):S222.
120. Bronkhorst MW, Boye ND, Lomax MA, Vossen RH, Bakker J, Patka P, Van Lieshout EM: **Single-nucleotide polymorphisms in the Toll-like receptor pathway increase susceptibility to infections in severely injured trauma patients**. *J Trauma Acute Care Surg* 2013, **74**(3):862-870.
121. Bronkhorst M, Lomax MAZ, Vossen R, Bakker J, Patka P, van Lieshout EMM: **Risk of infection and sepsis in severely injured patients related to single nucleotide polymorphisms in the lectin pathway**. *British Journal of Surgery* 2013, **100**(13):1818-1826.
122. Zeng L, Zhang A-q, Gu W, Chen K-h, Jiang D-p, Zhang L-y, Du D-y, Hu P, Huang S-n, Wang H-y *et al*: **Clinical relevance of single nucleotide polymorphisms of the high mobility group box 1 protein gene in patients with major trauma in Southwest China**. *Surgery* 2012, **151**(3):427-436.
123. Zeng L, Zhang AQ, Gu W, Zhou J, Zhang LY, Du DY, Zhang M, Wang HY, Yan J, Yang C *et al*: **Identification of haplotype tag single nucleotide polymorphisms within the receptor for advanced glycation end products gene and their clinical relevance in patients with major trauma**. *Crit Care* 2012, **16**(4):R131.
124. Zeng L, Zhang AQ, Gu W, Zhou J, Zhang LY, Du DY, Zhang M, Wang HY, Jiang JX: **Identification of haplotype tag SNPs within the whole myeloid differentiation 2 gene and their clinical relevance in patients with major trauma**. *Shock* 2012, **37**(4):366-372. doi: 310.1097/SHK.1090b1013e3182498c3182498f.
125. Zeng L, Gu W, Zhang AQ, Zhang M, Zhang LY, Du DY, Huang SN, Jiang JX: **A functional variant of lipopolysaccharide binding protein predisposes to sepsis and organ dysfunction in patients with major trauma**. *Ann Surg* 2012, **255**(1):147-157. doi: 110.1097/SLA.1090b1013e3182389515.
126. Yin J, Yao CL, Liu CL, Song ZJ, Tong CY, Huang PZ: **Association of genetic variants in the IRAK-4 gene with susceptibility to severe sepsis**. *World Journal of Emergency Medicine* 2012, **3**(2):123-127.
127. Wingeyer SP, Cunto E, Nogueras C, Juan JS, Norberto G, de Larranaga G: **Biomarkers in sepsis at time zero: Intensive care unit scores, plasma measurements and polymorphisms in Argentina**. *Journal of infection in developing countries* 2012, **6**(7):555-562.
128. West TE, Chierakul W, Chantratita N, Limmathurotsakul D, Wuthiekanun V, Emond MJ, Hawn TR, Peacock SJ, Skerrett SJ: **Toll-like receptor 4 region genetic variants are associated with susceptibility to melioidosis**. *Genes Immun* 2012, **13**(1):38-46.
129. Weiss SL, Yu M, Jennings L, Haymond S, Zhang G, Wainwright MS: **Pilot study of the association of the DDAH2 -449G polymorphism with asymmetric dimethylarginine and hemodynamic shock in pediatric sepsis**. *PloS one* 2012, **7**(3):e33355.
130. Wan QQ, Ye QF, Ma Y, Zhou JD: **Genetic association of interleukin-1(beta) (-511C/T) and its receptor antagonist (86-bpVNTR) gene polymorphism with susceptibility to bacteremia in kidney transplant recipients**. *Transplantation Proceedings* 2012, **44**(10):3026-3028.
131. Tekin D, Dalgic N, Kayaalti Z, Soylemezoglu T, Diler B, Kutlubay BI: **Importance of NOD2/CARD15 gene variants for susceptibility to and outcome of sepsis in Turkish children**. *Pediatr Crit Care Med* 2012, **13**(2):e73-77.
132. Su L, Liu C, Li C, Jiang Z, Xiao K, Zhang X, Li M, Yan P, Feng D, Xie L: **Dynamic Changes in Serum Soluble Triggering Receptor Expressed on Myeloid Cells-1 (sTREM-1) and its Gene Polymorphisms are Associated with Sepsis Prognosis**. *Inflammation* 2012.
133. Song Z, Song Y, Yin J, Shen Y, Yao C, Sun Z, Jiang J, Zhu D, Zhang Y, Shen Q *et al*: **Genetic Variation in the TNF Gene Is Associated with Susceptibility to Severe Sepsis, but Not with Mortality**. *Plos One* 2012, **7**(9).
134. Rosentul DC, Plantinga TS, Scott WK, Alexander BD, van de Geer NMD, Perfect JR, Kullberg BJ, Johnson MD, Netea MG: **The impact of caspase-12 on susceptibility to candidemia**. *European Journal of Clinical Microbiology & Infectious Diseases* 2012, **31**(3):277-280.
135. Plantinga TS, Johnson MD, Scott WK, van de Vosse E, Velez Edwards DR, Smith PB, Alexander BD, Yang JC, Kremer D, Laird GM *et al*: **Toll-like receptor 1 polymorphisms increase susceptibility to candidemia**. *J Infect Dis* 2012, **205**(6):934-943.
136. Phumeetham S, Chat-Uthai N, Manavathongchai M, Viprakasit V: **Genetic association study of tumor necrosis factor-alpha with sepsis and septic shock in Thai pediatric patients**. *J Pediatr (Rio J)* 2012, **88**(5):417-422.
137. Palumbo AA, Forte GI, Pileri D, Vaccarino L, Conte F, D'Amelio L, Palmeri M, Triolo A, D'Arpa N, Scola L *et al*: **Analysis of IL-6, IL-10 and IL-17 genetic polymorphisms as risk factors for sepsis development in burned patients**. *Burns* 2012, **38**(2):208-213.
138. Ozkan H, Koksal N, Cetinkaya M, Kilic S, Celebi S, Oral B, Budak F: **Serum mannose-binding lectin (MBL) gene polymorphism and low MBL levels are associated with neonatal sepsis and pneumonia**. *J Perinatol* 2012, **32**(3):210-217.
139. Nakazawa K, Kotani N, Goto K, Nomura M, Ozaki M: **Insertion/deletion polymorphism of the angiotensin-converting enzyme considerably changes postoperative outcome**. *Journal of Clinical Anesthesia* 2012, **24**(8):631-638.
140. Martin-Loeches I, Sole-Violan J, Rodriguez de Castro F, Isabel Garcia-Laorden M, Borderias L, Blanquer J, Rajas O, Luisa Briones M, Aspa J, Herrera-Ramos E *et al*: **Variants at the promoter of the interleukin-6 gene are associated with severity and outcome of pneumococcal community-acquired pneumonia**. *Intensive Care Medicine* 2012, **38**(2):256-262.
141. Liu Y, Shao Y, Yu B, Sun L, Lv F: **Association of PBEF gene polymorphisms with acute lung injury, sepsis, and pneumonia in a northeastern Chinese population**. *Clin Chem Lab Med* 2012, **50**(11):1917-1922.
142. Kotsaki A, Raftogiannis M, Routsi C, Baziaka F, Kotanidou A, Antonopoulou A, Orfanos SE, Katsenos C, Koutoukas P, Plachouras D *et al*: **Genetic polymorphisms within tumor necrosis factor gene promoter region: a role for susceptibility to ventilator-associated pneumonia**. *Cytokine* 2012, **59**(2):358-363.
143. Johnson MD, Plantinga TS, Van De Vosse E, Velez Edwards DR, Smith PB, Alexander BD, Yang JC, Kremer D, Laird GM, Oosting M *et al*: **Cytokine gene polymorphisms and the outcome of invasive candidiasis: A prospective cohort study**. *Clinical Infectious Diseases* 2012, **54**(4):502-510.
144. Graebin P, Veit TD, Alho CS, Bogo Chies JA: **Polymorphic variants in exon 8 at the 3 ' UTR of HLA-G gene are associated with septic shock in critically ill patients**. *Tissue Antigens* 2012, **80**(1):97-97.
145. Geistlinger J, Du W, Groll J, Liu F, Hoegel J, Foehr KJ, Pasquarelli A, Schneider EM: **P2RX7 genotype association in severe sepsis identified by a novel Multi-Individual Array for rapid screening and replication of risk SNPs**. *Clinica Chimica Acta* 2012, **413**(1-2):39-47.
146. Garnacho-Montero J, Garcia-Cabrera E, Jimenez-Alvarez R, Diaz-Martin A, Revuelto-Rey J, Aznar-Martin J, Garnacho-Montero C: **Genetic variants of the MBL2 gene are associated with mortality in pneumococcal sepsis**. *Diagnostic Microbiology and Infectious Disease* 2012, **73**(1):39-44.
147. Fode P, Larsen AR, Feenstra B, Jespersgaard C, Skov RL, Stegger M, Fowler VG, Andersen PS: **Genetic variability in beta-defensins is not associated with susceptibility to staphylococcus aureus bacteremia**. *PloS one* 2012, **7**(2).
148. Bhadri VA, Beckett SM, Duncan C, Marshall GM, Ashton LJ: **Variation in Toll-like receptor 9 gene modifies the risk of infection in children treated for acute lymphoblastic leukemia**. *Leukemia and Lymphoma* 2012, **53**(9):1828-1830.
149. Azevedo ZM, Moore DB, Lima FC, Cardoso CC, Bougleux R, Matos GI, Luz RA, Xavier-Elsas P, Sampaio EP, Gaspar-Elsas MI *et al*: **Tumor necrosis factor (TNF) and lymphotoxin-alpha (LTA) single nucleotide polymorphisms: importance in ARDS in septic pediatric critically ill patients**. *Hum Immunol* 2012, **73**(6):661-667.
150. Zhang AQ, Zeng L, Gu W, Zhang LY, Zhou J, Jiang DP, Du DY, Hu P, Yang C, Yan J *et al*: **Clinical relevance of single nucleotide polymorphisms within the entire NLRP3 gene in patients with major blunt trauma**. *Crit Care* 2011, **15**(6):R280.
151. Song Z, Yin J, Yao C, Sun Z, Shao M, Zhang Y, Tao Z, Huang P, Tong C: **Variants in the Toll-interacting protein gene are associated with susceptibility to sepsis in the Chinese Han population**. *Crit Care* 2011, **15**(1):R12.
152. Sole-Violan J, Garcia-Laorden MI, Marcos-Ramos JA, de Castro FR, Rajas O, Borderias L, Briones ML, Herrera-Ramos E, Blanquer J, Aspa J *et al*: **The Fcgamma receptor IIA-H/H131 genotype is associated with bacteremia in pneumococcal community-acquired pneumonia**. *Crit Care Med* 2011, **39**(6):1388-1393.
153. Shimada T, Oda S, Sadahiro T, Nakamura M, Hirayama Y, Watanabe E, Abe R, Nakada T-a, Tateishi Y, Otani S *et al*: **Outcome prediction in sepsis combined use of genetic polymorphisms - A study in Japanese population**. *Cytokine* 2011, **54**(1):79-84.
154. Rosentul DC, Plantinga TS, Oosting M, Scott WK, Velez Edwards DR, Smith PB, Alexander BD, Yang JC, Laird GM, Joosten LA *et al*: **Genetic variation in the dectin-1/CARD9 recognition pathway and susceptibility to candidemia**. *J Infect Dis* 2011, **204**(7):1138-1145.
155. Qian J, Pujiang, Ju S: **Correlations Between Allelic Polymorphism of TNF beta in 1069 Locus and Severe Post-Trauma Sepsis**. *Labmedicine* 2011, **42**(4):217-219.
156. Pino-Yanes M, Ma SF, Sun X, Tejera P, Corrales A, Blanco J, Perez-Mendez L, Espinosa E, Muriel A, Blanch L *et al*: **Interleukin-1 receptor-associated kinase 3 gene associates with susceptibility to acute lung injury**. *Am J Respir Cell Mol Biol* 2011, **45**(4):740-745.
157. Paskulin DD, Fallavena PR, Paludo FJ, Borges TJ, Picanco JB, Dias FS, Alho CS: **TNF -308G > a promoter polymorphism (rs1800629) and outcome from critical illness**. *Braz J Infect Dis* 2011, **15**(3):231-238.
158. Nakada TA, Russell JA, Boyd JH, Walley KR: **IL17A genetic variation is associated with altered susceptibility to Gram-positive infection and mortality of severe sepsis**. *Crit Care* 2011, **15**(5):R254.
159. Ma SF, Xie L, Pino-Yanes M, Sammani S, Wade MS, Letsiou E, Siegler J, Wang T, Infusino G, Kittles RA *et al*: **Type 2 deiodinase and host responses of sepsis and acute lung injury**. *Am J Respir Cell Mol Biol* 2011, **45**(6):1203-1211.
160. Ma P, Zhu Y, Qiu H, Liu J, Wang Y, Zeng L: **Endothelial nitric oxide synthase 894G-->T but not -786T-->C gene polymorphism is associated with organ dysfunction and increased mortality in patients with severe sepsis**. *J Trauma* 2011, **71**(4):872-877.
161. Lingappa JR, Dumitrescu L, Zimmer SM, Lynfield R, McNicholl JM, Messonnier NE, Whitney CG, Crawford DC: **Identifying Host Genetic Risk Factors in the Context of Public Health Surveillance for Invasive Pneumococcal Disease**. *PLoS ONE* 2011, **6**(8).
162. Lee S-O, Brown RA, Kang SH, Massih RCA, Razonable RR: **Toll-Like Receptor 4 Polymorphisms and the Risk of Gram-Negative Bacterial Infections After Liver Transplantation**. *Transplantation* 2011, **92**(6):690-696.
163. Lee SO, Brown RA, Kang SH, Abdel-Massih RC, Razonable RR: **Toll-like receptor 2 polymorphism and Gram-positive bacterial infections after liver transplantation**. *Liver Transpl* 2011, **17**(9):1081-1088.
164. Hartel C, Hemmelmann C, Faust K, Gebauer C, Hoehn T, Kribs A, Laux R, Nikischin W, Segerer H, Teig N *et al*: **Tumor necrosis factor-alpha promoter -308 G/A polymorphism and susceptibility to sepsis in very-low-birth-weight infants**. *Crit Care Med* 2011, **39**(5):1190-1195.
165. Gu W, Zeng L, Zhang LY, Jiang DP, Du DY, Hu P, Wang HY, Liu Q, Huang SN, Jiang JX: **Association of interleukin 4 -589T/C polymorphism with T(H)1 and T(H)2 bias and sepsis in Chinese major trauma patients**. *J Trauma* 2011, **71**(6):1583-1587.
166. Fang Y, Zhang L, Zhou GQ, Wang ZF, Zeng ZS, Luo ZY, Li L, Liu BC: **Functional polymorphism in exon 5 and variant haplotype of the interleukin-1 receptor-associated kinase 1 gene are associated with susceptibility to and severity of sepsis in the Chinese population**. *Chin Med J (Engl)* 2011, **124**(15):2248-2253.
167. Duan ZX, Gu W, Zhang LY, Jiang DP, Zhou J, Du DY, Zen L, Chen KH, Liu Q, Jiang JX: **Tumor necrosis factor alpha gene polymorphism is associated with the outcome of trauma patients in Chinese Han population**. *J Trauma* 2011, **70**(4):954-958. doi: 910.1097/TA.1090b1013e3181e1088adf.
168. Delongui F, Carvalho Grion CM, Ehara Watanabe MA, Morimoto HK, Bonametti AM, Maeda Oda JM, Kallaur AP, Matsuo T, Reiche EM: **Association of tumor necrosis factor beta genetic polymorphism and sepsis susceptibility**. *Exp Ther Med* 2011, **2**(2):349-356.
169. Chen KH, Zeng L, Gu W, Zhou J, Du DY, Jiang JX: **Polymorphisms in the toll-like receptor 9 gene associated with sepsis and multiple organ dysfunction after major blunt trauma**. *Br J Surg* 2011, **98**(9):1252-1259. doi: 1210.1002/bjs.7532. Epub 2011 Jun 1251.
170. Chen KH, Gu W, Zeng L, Jiang DP, Zhang LY, Zhou J, Du DY, Hu P, Liu Q, Huang SN *et al*: **Identification of haplotype tag SNPs within the entire TLR2 gene and their clinical relevance in patients with major trauma**. *Shock* 2011, **35**(1):35-41. doi: 10.1097/SHK.1090b1013e3181eb1045b1093.
171. Aydemir C, Onay H, Oguz SS, Ozdemir TR, Erdeve O, Ozkinay F, Dilmen U: **Mannose-binding lectin codon 54 gene polymorphism in relation to risk of nosocomial invasive fungal infection in preterm neonates in the neonatal intensive care unit**. *Journal of Maternal-Fetal and Neonatal Medicine* 2011, **24**(9):1124-1127.
172. Ahmad-Nejad P, Denz C, Zimmer W, Wacker J, Bugert P, Weiss C, Quintel M, Neumaier M: **The presence of functionally relevant toll-like receptor polymorphisms does not significantly correlate with development or outcome of sepsis**. *Genet Test Mol Biomarkers* 2011, **15**(9):645-651.
173. Wingeyer SP, de Larranaga G, Cunto E, Fontana L, Nogueras C, San Juan J: **Role of 4G/5G promoter polymorphism of Plasminogen Activator Inhibitor-1 (PAI-1) gene in outcome of sepsis**. *Thromb Res* 2010, **125**(4):367-369.
174. Watanabe E, Buchman TG, Hirasawa H, Zehnbauer BA: **Association between lymphotoxin-alpha (tumor necrosis factor-beta) intron polymorphism and predisposition to severe sepsis is modified by gender and age**. *Crit Care Med* 2010, **38**(1):181-193.
175. Toubiana J, Courtine E, Pene F, Viallon V, Asfar P, Daubin C, Rousseau C, Chenot C, Ouaaz F, Grimaldi D *et al*: **IRAK1 functional genetic variant affects severity of septic shock**. *Crit Care Med* 2010, **38**(12):2287-2294.
176. Thurow HS, Sarturi CR, Fallavena PR, Paludo FJ, Picanco JB, Fraga LR, Graebin P, de Souza VC, Dias FS, Nobrega Ode T *et al*: **Very low frequencies of Toll-like receptor 2 supposed-2029T and 2258A (RS5743708) mutant alleles in southern Brazilian critically ill patients: would it be a lack of worldwide-accepted clinical applications of Toll-like receptor 2 variants?** *Genet Test Mol Biomarkers* 2010, **14**(3):405-419.
177. Stanilova SA, Miteva LD, Stanilov NS, Stefanov CS, Karakolev ZT: **Interleukin-12b Polyrnorphisms in Association With Susceptibility to Severe Sepsis**. *Labmedicine* 2010, **41**(1):47-50.
178. Spiegler J, Gilhaus A, Konig IR, Kattner E, Vochem M, Kuster H, Moller J, Muller D, Kribs A, Segerer H *et al*: **Polymorphisms in the Renin-Angiotensin system and outcome of very-low-birthweight infants**. *Neonatology* 2010, **97**(1):10-14.
179. Song Z, Tong C, Sun Z, Shen Y, Yao C, Jiang J, Yin J, Gao L, Song Y, Bai C: **Genetic variants in the TIRAP gene are associated with increased risk of sepsis-associated acute lung injury**. *BMC Med Genet* 2010, **11**:168.
180. Sole-Violan J, de Castro F, Garcia-Laorden MI, Blanquer J, Aspa J, Borderias L, Briones ML, Rajas O, Carrondo IM, Marcos-Ramos JA *et al*: **Genetic variability in the severity and outcome of community-acquired pneumonia**. *Respir Med* 2010, **104**(3):440-447.
181. Smithson A, Perello R, Aibar J, Espinosa G, Tassies D, Freire C, Castro P, Suarez B, Lozano F, Nicolas JM: **Genotypes coding for low serum levels of mannose-binding lectin are underrepresented among individuals suffering from noninfectious systemic inflammatory response syndrome**. *Clinical and Vaccine Immunology* 2010, **17**(3):447-453.
182. Sipahi T, Kuybulu A, Ozturk A, Akar N: **Protein Z G79A polymorphism in patients with severe sepsis**. *Clin Appl Thromb Hemost* 2010, **16**(3):334-336.
183. Neth OW, Bacher U, Das P, Zabelina T, Kabisch H, Kroeger N, Ayuk F, Lioznov M, Waschke O, Fehse B *et al*: **Influence of mannose-binding lectin genotypes and serostatus in allo-SCT: analysis of 131 recipients and donors**. *Bone Marrow Transplantation* 2010, **45**(1):13-19.
184. Kumpf O, Giamarellos-Bourboulis EJ, Koch A, Hamann L, Mouktaroudi M, Oh DY, Latz E, Lorenz E, Schwartz DA, Ferwerda B *et al*: **Influence of genetic variations in TLR4 and TIRAP/Mal on the course of sepsis and pneumonia and cytokine release: an observational study in three cohorts**. *Crit Care* 2010, **14**(3):R103.
185. Koroglu OA, Onay H, Erdemir G, Yalaz M, Cakmak B, Akisu M, Ozkinay F, Kultursay N: **Mannose-binding lectin gene polymorphism and early neonatal outcome in preterm infants**. *Neonatology* 2010, **98**(4):305-312.
186. Klostergaard A, Steffensen R, Moller JK, Peterslund N, Juhl-Christensen C, Molle I: **Sepsis in acute myeloid leukaemia patients receiving high-dose chemotherapy: no impact of chitotriosidase and mannose-binding lectin polymorphisms**. *Eur J Haematol* 2010, **85**(1):58-64.
187. Khor CC, Vannberg FO, Chapman SJ, Guo H, Wong SH, Walley AJ, Vukcevic D, Rautanen A, Mills TC, Chang KC *et al*: **CISH and susceptibility to infectious diseases**. *N Engl J Med* 2010, **362**(22):2092-2101.
188. Joannes MO, Loko G, Deloumeaux J, Chout R, Marianne-Pepin T: **Association of the +874 T/A interferon gamma polymorphism with infections in sickle cell disease**. *Int J Immunogenet* 2010, **37**(4):219-223.
189. Jin Y, Zhang Y, Wang H, Wu S, Chen Q, Cheng B, Xie G, Hu Y, Fang X: **Genomic polymorphisms within alpha 7 nicotinic acetylcholine receptor and severe sepsis in Chinese Han population**. *Int J Immunogenet* 2010, **37**(5):361-365.
190. Huebinger RM, Rivera-Chavez F, Chang L-Y, Liu M-M, Minei JP, Purdue GF, Hunt JL, Arnoldo BD, Barber RC: **IL-10 Polymorphism Associated with Decreased Risk for Mortality After Burn Injury**. *Journal of Surgical Research* 2010, **164**(1):E141-E145.
191. Huebinger RM, Gomez R, McGee D, Chang LY, Bender JE, O'Keeffe T, Burris AM, Friese SM, Purdue GF, Hunt JL *et al*: **Association of mitochondrial allele 4216C with increased risk for sepsis-related organ dysfunction and shock after burn injury**. *Shock* 2010, **33**(1):19-23.
192. Guarner-Argente C, Sanchez E, Vidal S, RomAn E, ConcepciOn M, Poca M, Sanchez D, Juarez C, Soriano G, Guarner C: **Toll-like receptor 4 D299G polymorphism and the incidence of infections in cirrhotic patients**. *Alimentary Pharmacology and Therapeutics* 2010, **31**(11):1192-1199.
193. Gu W, Zeng L, Zhou J, Jiang D-p, Zhang L, Du D-y, Hu P, Chen K, Liu Q, Wang Z-g *et al*: **Clinical relevance of 13 cytokine gene polymorphisms in Chinese major trauma patients**. *Intensive Care Medicine* 2010, **36**(7):1261-1265.
194. Emonts M, Vermont CL, Houwing-Duistermaat JJ, Haralambous E, Gaast-de Jongh CE, Hazelzet JA, Faust SN, Betts H, Hermans PWM, Levin M *et al*: **POLYMORPHISMS IN PARP, IL1B, IL4, IL10, C1INH, DEFB1, AND DEFA4 IN MENINGOCOCCAL DISEASE IN THREE POPULATIONS**. *Shock* 2010, **34**(1):17-22.
195. de Rooij B-JF, van Hoek B, ten Hove WR, Roos A, Bouwman LH, Schaapherder AF, Porte RJ, Daha MR, van der Reijden JJ, Coenraad MJ *et al*: **Lectin Complement Pathway Gene Profile of Donor and Recipient Determine the Risk of Bacterial Infections After Orthotopic Liver Transplantation**. *Hepatology* 2010, **52**(3):1100-1110.
196. Davis SM, Clark EA, Nelson LT, Silver RM: **The association of innate immune response gene polymorphisms and puerperal group A streptococcal sepsis**. *Am J Obstet Gynecol* 2010, **202**(3):308 e301-308.
197. Chen Q, Hakimi M, Wu S, Jin Y, Cheng B, Wang H, Xie G, Ganz T, Linzmeier RM, Fang X: **Increased genomic copy number of DEFA1/DEFA3 is associated with susceptibility to severe sepsis in Chinese Han population**. *Anesthesiology* 2010, **112**(6):1428-1434.
198. Chen K, Wang YT, Gu W, Zeng L, Jiang DP, Du DY, Hu P, Duan ZX, Liu Q, Huang SN *et al*: **Functional significance of the Toll-like receptor 4 promoter gene polymorphisms in the Chinese Han population**. *Crit Care Med* 2010, **38**(5):1292-1299. doi: 1210.1097/CCM.1290b1013e3181d1298ad1212.
199. Chapman SJ, Khor CC, Vannberg FO, Rautanen A, Walley A, Segal S, Moore CE, Davies RJ, Day NP, Peshu N *et al*: **Common NFKBIL2 polymorphisms and susceptibility to pneumococcal disease: a genetic association study**. *Crit Care* 2010, **14**(6):R227.
200. Celik U, Yildizdas D, Celik T, Alhan E, Atila G, Tepe T, Sertdemir Y: **Relationship Between Angiotensin-Converting Enzyme Gene Polymorphism (Insertion/Deletion) and the Clinical Condition of Sepsis in Turkish Children**. *Turkiye Klinikleri Tip Bilimleri Dergisi* 2010, **30**(2):591-597.
201. Carregaro F, Carta A, Cordeiro JA, Lobo SM, Silva EH, Leopoldino AM: **Polymorphisms IL10-819 and TLR-2 are potentially associated with sepsis in Brazilian patients**. *Mem Inst Oswaldo Cruz* 2010, **105**(5):649-656.
202. Benfield T, Ejrnaes K, Juul K, Ostergaard C, Helweg-Larsen J, Weis N, Munthe-Fog L, Kronborg G, Andersen MR, Tybjaerg-Hansen A *et al*: **Influence of Factor V Leiden on susceptibility to and outcome from critical illness: a genetic association study**. *Crit Care* 2010, **14**(2):R28.
203. Auriti C, Prencipe G, Inglese R, Azzari C, Ronchetti MP, Tozzi A, Seganti G, Orzalesi M, De Benedetti F: **Role of mannose-binding lectin in nosocomial sepsis in critically ill neonates**. *Hum Immunol* 2010, **71**(11):1084-1088.
204. Worthley DL, Johnson DF, Eisen DP, Dean MM, Heatley SL, Tung JP, Scott J, Padbury RT, Harley HA, Bardy PG *et al*: **Donor mannose-binding lectin deficiency increases the likelihood of clinically significant infection after liver transplantation**. *Clin Infect Dis* 2009, **48**(4):410-417.
205. Read RC, Teare DM, Pridmore AC, Naylor SC, Timms JM, Kaczmarski EB, Borrow R, Wilson AG: **The tumor necrosis factor polymorphism TNF (-308) is associated with susceptibility to meningococcal sepsis, but not with lethality**. *Crit Care Med* 2009, **37**(4):1237-1243.
206. Plantinga TS, Van Der Velden WJFM, Ferwerda B, Van Spriel AB, Adema G, Feuth T, Peter Donnelly J, Brown GD, Kullberg BJ, Blijlevens NMA *et al*: **Early stop polymorphism in human DECTIN-1 is associated with increased candida colonization in hematopoietic stem cell transplant recipients**. *Clinical Infectious Diseases* 2009, **49**(5):724-732.
207. Ozgur TT, Yel L, Yigit S, Mesci L, Sanal O, Tezcan I, Tekinalp G, Ersoy F: **Lack of association between TLR4 polymorphism and severe gram-negative bacterial infection in neonates**. *Turkish Journal of Medical Sciences* 2009, **39**(3):423-427.
208. Motoyama S, Miura M, Hinai Y, Maruyama K, Usami S, Nakatsu T, Saito H, Minamiya Y, Murata K, Suzuki T *et al*: **Interferon-gamma 874A>T genetic polymorphism is associated with infectious complications following surgery in patients with thoracic esophageal cancer**. *Surgery* 2009, **146**(5):931-938.
209. Mensah NY, Peterlongo P, Steinherz P, Pamer EG, Satagopan J, Papanicolaou GA: **Toll-like receptor 4 polymorphisms and risk of gram-negative bacteremia after allogeneic stem cell transplantation. A prospective pilot study**. *Biol Blood Marrow Transplant* 2009, **15**(9):1130-1133.
210. Lin J, Yao Y-m, Dong N, Chai J-k, Yu Y, Hou X-x, Zhu J-m, Sheng Z-y: **Influence of CD14 polymorphism on CD14 expression in patients with extensive burns**. *Burns* 2009, **35**(3):365-371.
211. Lehmann LE, Book M, Hartmann W, Weber SU, Schewe JC, Klaschik S, Hoeft A, Stuber F: **A MIF haplotype is associated with the outcome of patients with severe sepsis: a case control study**. *J Transl Med* 2009, **7**:100.
212. Kleiman DA, Calvano JE, Coyle SM, Macor MA, Calvano SE, Lowry SF: **A single nucleotide polymorphism in the Mdm2 promoter and risk of sepsis**. *Am J Surg* 2009, **197**(1):43-48.
213. Huh JW, Song K, Yum JS, Hong SB, Lim CM, Koh Y: **Association of mannose-binding lectin-2 genotype and serum levels with prognosis of sepsis**. *Crit Care* 2009, **13**(6):R176.
214. Horcajada JP, Lozano F, Munoz A, Suarez B, Farinas-Alvarez C, Almela M, Smithson A, Martinez E, Mallolas J, Mensa J *et al*: **Polymorphic receptors of the innate immune system (MBL/MASP-2 and TLR2/4) and susceptibility to pneumococcal bacteremia in HIV-infected patients: a case-control study**. *Curr HIV Res* 2009, **7**(2):218-223.
215. Hildebrand F, Kalmbach M, Kaapke A, Krettek C, Stuhrmann M: **No association between CALCA polymorphisms and clinical outcome or serum procalcitonin levels in German polytrauma patients**. *Cytokine* 2009, **47**(1):30-36.
216. Henckaerts L, Nielsen KR, Steffensen R, Van Steen K, Mathieu C, Giulietti A, Wouters PJ, Milants I, Vanhorebeek I, Langouche L *et al*: **Polymorphisms in innate immunity genes predispose to bacteremia and death in the medical intensive care unit**. *Crit Care Med* 2009, **37**(1):192-201, e191-193.
217. Hamann L, Kumpf O, Schuring RP, Alpsoy E, Bedu-Addo G, Bienzle U, Oskam L, Mockenhaupt FP, Schumann RR: **Low frequency of the TIRAP S180L polymorphism in Africa, and its potential role in malaria, sepsis, and leprosy**. *BMC Med Genet* 2009, **10**:65.
218. Gomez R, O'Keeffe T, Chang LY, Huebinger RM, Minei JP, Barber RC: **Association of mitochondrial allele 4216C with increased risk for complicated sepsis and death after traumatic injury**. *J Trauma* 2009, **66**(3):850-857; discussion 857-858.
219. Flores C, Perez-Mendez L, Maca-Meyer N, Muriel A, Espinosa E, Blanco J, Sanguesa R, Muros M, Garcia JG, Villar J: **A common haplotype of the LBP gene predisposes to severe sepsis**. *Crit Care Med* 2009, **37**(10):2759-2766.
220. Ferwerda B, Alonso S, Banahan K, McCall MB, Giamarellos-Bourboulis EJ, Ramakers BP, Mouktaroudi M, Fain PR, Izagirre N, Syafruddin D *et al*: **Functional and genetic evidence that the Mal/TIRAP allele variant 180L has been selected by providing protection against septic shock**. *Proc Natl Acad Sci U S A* 2009, **106**(25):10272-10277.
221. Fallavena PR, Borges TJ, Paskulin DD, Paludo FJ, Goetze TB, de Oliveira JR, Nobrega OT, Dias FS, Alho CS: **The influences of CD14 -260C>T polymorphism on survival in ICU critically ill patients**. *Immunol Invest* 2009, **38**(8):797-811.
222. Endeman H, Cornips MC, Grutters JC, van den Bosch JM, Ruven HJ, van Velzen-Blad H, Rijkers GT, Biesma DH: **The Fcgamma receptor IIA-R/R131 genotype is associated with severe sepsis in community-acquired pneumonia**. *Clin Vaccine Immunol* 2009, **16**(7):1087-1090.
223. Duan ZX, Gu W, Zhang LY, Du DY, Hu P, Huang J, Liu Q, Wang ZG, Hao J, Jiang JX: **Clinical relevance of the TLR4 11367 polymorphism in patients with major trauma**. *Arch Surg* 2009, **144**(12):1144-1148. doi: 1110.1001/archsurg.2009.1211.
224. Duan ZX, Gu W, Du DY, Hu P, Jiang DP, Zhu PF, Wang ZG, Jiang JX: **Distributions of glucocorticoid receptor gene polymorphisms in a Chinese Han population and associations with outcome after major trauma**. *Injury* 2009, **40**(5):479-483.
225. Cervera C, Balderramo D, Suarez B, Prieto J, Fuster F, Linares L, Fuster J, Moreno A, Lozano F, Navasa M: **Donor Mannose-Binding Lectin Gene Polymorphisms Influence the Outcome of Liver Transplantation**. *Liver Transplantation* 2009, **15**(10):1217-1224.
226. Abdel-Hady H, El-Naggar M, El-Nady G, Badr R, El-Daker M: **Genetic polymorphisms of IL-6-174 and IL-10-1082 in full term neonates with late onset blood stream infections**. *Journal of Pediatric Infectious Diseases* 2009, **4**(4):357-365.
227. Yuan FF, Marks K, Wong M, Watson S, de Leon E, McIntyre PB, Sullivan JS: **Clinical relevance of TLR2, TLR4, CD14 and FcgammaRIIA gene polymorphisms in Streptococcus pneumoniae infection**. *Immunol Cell Biol* 2008, **86**(3):268-270.
228. Yang Y, Shou Z, Zhang P, He Q, Xiao H, Xu Y, Li C, Chen J: **Mitochondrial DNA haplogroup R predicts survival advantage in severe sepsis in the Han population**. *Genet Med* 2008, **10**(3):187-192.
229. Wurfel MM, Gordon AC, Holden TD, Radella F, Strout J, Kajikawa O, Ruzinski JT, Rona G, Black RA, Stratton S *et al*: **Toll-like receptor 1 polymorphisms affect innate immune responses and outcomes in sepsis**. *Am J Respir Crit Care Med* 2008, **178**(7):710-720.
230. Villar J, Flores C, Perez-Mendez L, Maca-Meyer N, Espinosa E, Blanco J, Sanguesa R, Muriel A, Tejera P, Muros M *et al*: **Angiotensin-converting enzyme insertion/deletion polymorphism is not associated with susceptibility and outcome in sepsis and acute respiratory distress syndrome**. *Intensive Care Med* 2008, **34**(3):488-495.
231. van Till JW, Modderman PW, de Boer M, Hart MH, Beld MG, Boermeester MA: **Mannose-binding lectin deficiency facilitates abdominal Candida infections in patients with secondary peritonitis**. *Clin Vaccine Immunol* 2008, **15**(1):65-70.
232. van der Zwet WC, Catsburg A, van Elburg RM, Savelkoul PH, Vandenbroucke-Grauls CM: **Mannose-binding lectin (MBL) genotype in relation to risk of nosocomial infection in pre-term neonates in the neonatal intensive care unit**. *Clin Microbiol Infect* 2008, **14**(2):130-135.
233. Tukiainen E, Kylanpaa ML, Puolakkainen P, Kemppainen E, Halonen K, Orpana A, Methuen T, Salaspuro M, Haapiainen R, Repo H: **Polymorphisms of the TNF, CD14, and HSPA1B genes in patients with acute alcohol-induced pancreatitis**. *Pancreas* 2008, **37**(1):56-61.
234. Reiman M, Kujari H, Ekholm E, Lapinleimu H, Lehtonen L, Haataja L: **Interleukin-6 polymorphism is associated with chorioamnionitis and neonatal infections in preterm infants**. *J Pediatr* 2008, **153**(1):19-24.
235. Menges T, Konig IR, Hossain H, Little S, Tchatalbachev S, Thierer F, Hackstein H, Franjkovic I, Colaris T, Martens F *et al*: **Sepsis syndrome and death in trauma patients are associated with variation in the gene encoding tumor necrosis factor**. *Crit Care Med* 2008, **36**(5):1456-1462, e1451-1456.
236. Huttunen R, Aittoniemi J, Laine J, Vuento R, Karjalainen J, Rovio AT, Eklund C, Hurme M, Huhtala H, Syrjanen J: **Gene-environment interaction between MBL2 genotype and smoking, and the risk of gram-positive bacteraemia**. *Scand J Immunol* 2008, **68**(4):438-444.
237. Gu W, Dong H, Jiang DP, Zhou J, Du DY, Gao JM, Yao YZ, Zhang LY, Wen AQ, Liu Q *et al*: **Functional significance of CD14 promoter polymorphisms and their clinical relevance in a Chinese Han population**. *Crit Care Med* 2008, **36**(8):2274-2280. doi: 2210.1097/CCM.2270b2013e318180b318181ed.
238. Garcia-Laorden MI, Sole-Violan J, Rodriguez de Castro F, Aspa J, Briones ML, Garcia-Saavedra A, Rajas O, Blanquer J, Caballero-Hidalgo A, Marcos-Ramos JA *et al*: **Mannose-binding lectin and mannose-binding lectin-associated serine protease 2 in susceptibility, severity, and outcome of pneumonia in adults**. *J Allergy Clin Immunol* 2008, **122**(2):368-374, 374 e361-362.
239. Dzwonek AB, Neth OW, Thiebaut R, Gulczynska E, Chilton M, Hellwig T, Bajaj-Elliott M, Hawdon J, Klein NJ: **The role of mannose-binding lectin in susceptibility to infection in preterm neonates**. *Pediatr Res* 2008, **63**(6):680-685.
240. du Cheyron D, Fradin S, Ramakers M, Terzi N, Guillotin D, Bouchet B, Daubin C, Charbonneau P: **Angiotensin converting enzyme insertion/deletion genetic polymorphism: Its impact on renal function in critically ill patients**. *Critical Care Medicine* 2008, **36**(12):3178-3183.
241. de Aguiar BB, Girardi I, Paskulin DD, de Franca E, Dornelles C, Dias FS, Bonorino C, Alho CS: **CD14 expression in the first 24h of sepsis: effect of -260C>T CD14 SNP**. *Immunol Invest* 2008, **37**(8):752-769.
242. Cogulu O, Onay H, Uzunkaya D, Gunduz C, Pehlivan S, Vardar F, Atlihan F, Ozkinay C, Ozkinay F: **Role of angiotensin-converting enzyme gene polymorphisms in children with sepsis and septic shock**. *Pediatr Int* 2008, **50**(4):477-480.
243. Chien JW, Boeckh MJ, Hansen JA, Clark JG: **Lipopolysaccharide binding protein promoter variants influence the risk for Gram-negative bacteremia and mortality after allogeneic hematopoietic cell transplantation**. *Blood* 2008, **111**(4):2462-2469.
244. Chen QX, Wu SJ, Wang HH, Lv C, Cheng BL, Xie GH, Fang XM: **Protein C -1641A/-1654C haplotype is associated with organ dysfunction and the fatal outcome of severe sepsis in Chinese Han population**. *Hum Genet* 2008, **123**(3):281-287.
245. Chen Q, Zhou H, Wu S, Wang H, Lv C, Cheng B, Xie G, Fang X: **Lack of association between TREM-1 gene polymorphisms and severe sepsis in a Chinese Han population**. *Hum Immunol* 2008, **69**(3):220-226.
246. Celik U, Yildizdas D, Alhan E, Celik T, Attila G, Sertdemir Y, Tepe T: **Genetic dilemma: eNOS gene intron 4a/b VNTR polymorphism in sepsis and its clinical features in Turkish children**. *Turk J Pediatr* 2008, **50**(2):114-119.
247. Bunker-Wiersma HE, Koopmans RP, Kuipers TW, Knoester H, Bos AP: **Single nucleotide polymorphisms in genes of circulatory homeostasis in surviving pediatric intensive care patients with meningococcal infection**. *Pediatr Crit Care Med* 2008, **9**(5):517-523.
248. Weiss G, Madsen HO, Garred P: **A novel mannose-binding lectin-associated serine protease 1/3 gene variant**. *Scandinavian Journal of Immunology* 2007, **65**(5):430-434.
249. Michalek J, Svetlikova P, Fedora M, Klimovic M, Klapacova L, Bartosova D, Hrstkova H, Hubacek JA: **Interleukin-6 gene variants and the risk of sepsis development in children**. *Hum Immunol* 2007, **68**(9):756-760.
250. Michalek J, Svetlikova P, Fedora M, Klimovic M, Klapacova L, Bartosova D, Elbl L, Hrstkova H, Hubacek JA: **Bactericidal permeability increasing protein gene variants in children with sepsis**. *Intensive Care Medicine* 2007, **33**(12):2158-2164.
251. McDaniel DO, Hamilton J, Brock M, May W, Calcote L, Tee LY, Vick L, Newman DB, Vick K, Harrison S *et al*: **Molecular analysis of inflammatory markers in trauma patients at risk of postinjury complications**. *J Trauma* 2007, **63**(1):147-157; discussion 157-148.
252. Malik MH, Jury F, Bayat A, Ollier WE, Kay PR: **Genetic susceptibility to total hip arthroplasty failure: a preliminary study on the influence of matrix metalloproteinase 1, interleukin 6 polymorphisms and vitamin D receptor**. *Ann Rheum Dis* 2007, **66**(8):1116-1120.
253. Khor CC, Chapman SJ, Vannberg FO, Dunne A, Murphy C, Ling EY, Frodsham AJ, Walley AJ, Kyrieleis O, Khan A *et al*: **A Mal functional variant is associated with protection against invasive pneumococcal disease, bacteremia, malaria and tuberculosis**. *Nat Genet* 2007, **39**(4):523-528.
254. Hellemann D, Larsson A, Madsen HO, Bonde J, Jarlov JO, Wiis J, Faber T, Wetterslev J, Garred P: **Heterozygosity of mannose-binding lectin (MBL2) genotypes predicts advantage (heterosis) in relation to fatal outcome in intensive care patients**. *Hum Mol Genet* 2007, **16**(24):3071-3080.
255. Garcia-Segarra G, Espinosa G, Tassies D, Oriola J, Aibar J, Bove A, Castro P, Reverter J-C, Nicolas J-M: **Increased mortality in septic shock with the 4G/4G genotype of plasminogen activator inhibitor 1 in patients of white descent**. *Intensive Care Medicine* 2007, **33**(8):1354-1362.
256. Gao L, Flores C, Fan-Ma S, Miller EJ, Moitra J, Moreno L, Wadgaonkar R, Simon B, Brower R, Sevransky J *et al*: **Macrophage migration inhibitory factor in acute lung injury: expression, biomarker, and associations**. *Transl Res* 2007, **150**(1):18-29.
257. Frakking FNJ, Brouwer N, van Eijkelenburg NKA, Merkus MP, Kuijpers TW, Offringa M, Dolman KM: **Low mannose-binding lectin (MBL) levels in neonates with pneumonia and sepsis**. *Clinical and Experimental Immunology* 2007, **150**(2):255-262.
258. Everett B, Cameron B, Li H, Vollmer-Conna U, Davenport T, Hickie I, Wakefield D, Vernon S, Reeves WC, Lloyd AR: **Polymorphisms in Toll-like receptors-2 and -4 are not associated with disease manifestations in acute Q fever**. *Genes Immun* 2007, **8**(8):699-702.
259. Elsakka NE, Webster NR, Galley HF: **Polymorphism in the manganese superoxide dismutase gene**. *Free Radical Research* 2007, **41**(7):770-778.
260. Chen QX, Lv C, Huang LX, Cheng BL, Xie GH, Wu SJ, Fang XM: **Genomic variations within DEFB1 are associated with the susceptibility to and the fatal outcome of severe sepsis in Chinese Han population**. *Genes Immun* 2007, **8**(5):439-443.
261. Azim K, McManus R, Brophy K, Ryan A, Kelleher D, Reynolds JV: **Genetic polymorphisms and the risk of infection following esophagectomy. positive association with TNF-alpha gene -308 genotype**. *Ann Surg* 2007, **246**(1):122-128.
262. Yoon HJ, Choi JY, Kim CO, Park YS, Kim MS, Kim YK, Shin SY, Kim JM, Song YG: **Lack of Toll-like receptor 4 and 2 polymorphisms in Korean patients with bacteremia**. *J Korean Med Sci* 2006, **21**(6):979-982.
263. Van der Graaf CA, Netea MG, Morre SA, Den Heijer M, Verweij PE, Van der Meer JW, Kullberg BJ: **Toll-like receptor 4 Asp299Gly/Thr399Ile polymorphisms are a risk factor for Candida bloodstream infection**. *Eur Cytokine Netw* 2006, **17**(1):29-34.
264. Stanilova SA, Miteva LD, Karakolev ZT, Stefanov CS: **Interleukin-10-1082 promoter polymorphism in association with cytokine production and sepsis susceptibility**. *Intensive Care Med* 2006, **32**(2):260-266.
265. Sipahi T, Pocan H, Akar N: **Effect of various genetic polymorphisms on the incidence and outcome of severe sepsis**. *Clin Appl Thromb Hemost* 2006, **12**(1):47-54.
266. Schueller AC, Heep A, Kattner E, Kroll M, Wisbauer M, Sander J, Bartmann P, Stuber F: **Prevalence of two tumor necrosis factor gene polymorphisms in premature infants with early onset sepsis**. *Biol Neonate* 2006, **90**(4):229-232.
267. Molle I, Peterslund NA, Thiel S, Steffensen R: **MBL2 polymorphism and risk of severe infections in multiple myeloma patients receiving high-dose melphalan and autologous stem cell transplantation**. *Bone Marrow Transplantation* 2006, **38**(8):555-560.
268. Hartel C, Konig I, Koster S, Kattner E, Kuhls E, Kuster H, Moller J, Muller D, Kribs A, Segerer H *et al*: **Genetic polymorphisms of hemostasis genes and primary outcome of very low birth weight infants**. *Pediatrics* 2006, **118**(2):683-689.
269. Gordon AC, Waheed U, Hansen TK, Hitman GA, Garrard CS, Turner MW, Klein NJ, Brett SJ, Hinds CJ: **Mannose-binding lectin polymorphisms in severe sepsis: relationship to levels, incidence, and outcome**. *Shock* 2006, **25**(1):88-93.
270. Gopel W, Hartel C, Ahrens P, Konig I, Kattner E, Kuhls E, Kuster H, Moller J, Muller D, Roth B *et al*: **Interleukin-6-174-genotype, sepsis and cerebral injury in very low birth weight infants**. *Genes Immun* 2006, **7**(1):65-68.
271. Garnacho-Montero J, Aldabo-Pallas T, Garnacho-Montero C, Cayuela A, Jimenez R, Barroso S, Ortiz-Leyba C: **Timing of adequate antibiotic therapy is a greater determinant of outcome than are TNF and IL-10 polymorphisms in patients with sepsis**. *Crit Care* 2006, **10**(4):R111.
272. Gao L, Grant A, Halder I, Brower R, Sevransky J, Maloney JP, Moss M, Shanholtz C, Yates CR, Meduri GU *et al*: **Novel polymorphisms in the myosin light chain kinase gene confer risk for acute lung injury**. *Am J Respir Cell Mol Biol* 2006, **34**(4):487-495.
273. Flores C, Maca-Meyer N, Perez-Mendez L, Sanguesa R, Espinosa E, Muriel A, Blanco J, Villar J: **A CXCL2 tandem repeat promoter polymorphism is associated with susceptibility to severe sepsis in the Spanish population**. *Genes Immun* 2006, **7**(2):141-149.
274. Eisen DP, Dean MM, Thomas P, Marshall P, Gerns N, Heatley S, Quinn J, Minchinton RM, Lipman J: **Low mannose-binding lectin function is associated with sepsis in adult patients**. *Fems Immunology and Medical Microbiology* 2006, **48**(2):274-282.
275. D'Avila LC, Albarus MH, Franco CR, Aguiar BB, Oliveira JR, Dias FS, Alho CS: **Effect of CD14 -260C>T polymorphism on the mortality of critically ill patients**. *Immunol Cell Biol* 2006, **84**(4):342-348.
276. Bowers DJ, Calvano JE, Alvarez SM, Coyle SM, Macor MA, Kumar A, Calvano SE, Lowry SF: **Polymorphisms of heat shock protein-70 (HSPA1B and HSPA1L loci) do not influence infection or outcome risk in critically ill surgical patients**. *Shock* 2006, **25**(2):117-122.
277. Barber RC, Chang LY, Arnoldo BD, Purdue GF, Hunt JL, Horton JW, Aragaki CC: **Innate immunity SNPs are associated with risk for severe sepsis after burn injury**. *Clin Med Res* 2006, **4**(4):250-255.
278. Baier RJ, Loggins J, Yanamandra K: **IL-10, IL-6 and CD14 polymorphisms and sepsis outcome in ventilated very low birth weight infants**. *BMC Med* 2006, **4**:10.
279. Zhang DL, Zheng HM, Yu BJ, Jiang ZW, Li JS: **Association of polymorphisms of IL and CD14 genes with acute severe pancreatitis and septic shock**. *World J Gastroenterol* 2005, **11**(28):4409-4413.
280. Ye SQ, Simon BA, Maloney JP, Zambelli-Weiner A, Gao L, Grant A, Easley RB, McVerry BJ, Tuder RM, Standiford T *et al*: **Pre-B-cell colony-enhancing factor as a potential novel biomarker in acute lung injury**. *Am J Respir Crit Care Med* 2005, **171**(4):361-370.
281. Watanabe E, Hirasawa H, Oda S, Shiga H, Matsuda K, Nakamura M, Abe R, Nakada T: **Cytokine-related genotypic differences in peak interleukin-6 blood levels of patients with SIRS and septic complications**. *J Trauma* 2005, **59**(5):1181-1189; discussion 1189-1190.
282. Schaaf B, Rupp J, Muller-Steinhardt M, Kruse J, Boehmke F, Maass M, Zabel P, Dalhoff K: **The interleukin-6-174 promoter polymorphism is associated with extrapulmonary bacterial dissemination in Streptococcus pneumoniae infection**. *Cytokine* 2005, **31**(4):324-328.
283. Nakada TA, Hirasawa H, Oda S, Shiga H, Matsuda K, Nakamura M, Watanabe E, Abe R, Hatano M, Tokuhisa T: **Influence of toll-like receptor 4, CD14, tumor necrosis factor, and interleukine-10 gene polymorphisms on clinical outcome in Japanese critically ill patients**. *J Surg Res* 2005, **129**(2):322-328.
284. Moretti EW, Morris RW, Podgoreanu M, Schwinn DA, Newman MF, Bennett E, Moulin VG, Mba UU, Laskowitz DT: **APOE polymorphism is associated with risk of severe sepsis in surgical patients**. *Crit Care Med* 2005, **33**(11):2521-2526.
285. Lehrnbecher T, Bernig T, Hanisch M, Koehl U, Behl M, Reinhardt D, Creutzig U, Klingebiel T, Chanock SJ, Schwabe D: **Common genetic variants in the interleukin-6 and chitotriosidase genes are associated with the risk for serious infection in children undergoing therapy for acute myeloid leukemia**. *Leukemia* 2005, **19**(10):1745-1750.
286. Horiuchi T, Gondo H, Miyagawa H, Otsuka J, Inaba S, Nagafuji K, Takase K, Tsukamoto H, Koyama T, Mitoma H *et al*: **Association of MBL gene polymorphisms with major bacterial infection in patients treated with high-dose chemotherapy and autologus PBSCT**. *Genes and Immunity* 2005, **6**(2):162-166.
287. Geishofer G, Binder A, Muller M, Zohrer B, Resch B, Muller W, Faber J, Finn A, Endler G, Mannhalter C *et al*: **4G/5G promoter polymorphism in the plasminogen-activator-inhibitor-1 gene in children with systemic meningococcaemia**. *Eur J Pediatr* 2005, **164**(8):486-490.
288. Bouwman LH, Roos A, Terpstra OT, de Knijff P, van Hoek B, Verspaget HW, Berger SP, Daha MR, Frolich M, van der Slik AR *et al*: **Mannose binding lectin gene polymorphisms confer a major risk for severe infections after liver transplantation**. *Gastroenterology* 2005, **129**(2):408-414.
289. Baier RJ, Loggins J, Yanamandra K: **Angiotensin converting enzyme insertion/deletion polymorphism does not alter sepsis outcome in ventilated very low birth weight infants**. *Journal of Perinatology* 2005, **25**(3):205-209.
290. Saleh M, Vaillancourt JP, Graham RK, Huyck M, Srinivasula SM, Alnemri ES, Steinberg MH, Nolan V, Baldwin CT, Hotchkiss RS *et al*: **Differential modulation of endotoxin responsiveness by human caspase-12 polymorphisms**. *Nature* 2004, **429**(6987):75-79.
291. Quasney MW, Waterer GW, Dahmer MK, Kron GK, Zhang Q, Kessler LA, Wunderink RG: **Association between surfactant protein B + 1580 polymorphism and the risk of respiratory failure in adults with community-acquired pneumonia**. *Crit Care Med* 2004, **32**(5):1115-1119.
292. Moore CE, Segal S, Berendt AR, Hill AVS, Day NPJ: **Lack of association between toll-like receptor 2 polymorphisms and susceptibility to severe disease caused by Staphylococcus aureus**. *Clinical and Diagnostic Laboratory Immunology* 2004, **11**(6):1194-1197.
293. Kahlke V, Schafmayer C, Schniewind B, Seegert D, Schreiber S, Schroder J: **Are postoperative complications genetically determined by TNF-beta NcoI gene polymorphism?** *Surgery* 2004, **135**(4):365-373; discussion 374-365.
294. Hedberg CL, Adcock K, Martin J, Loggins J, Kruger TE, Baier RJ: **Tumor necrosis factor alpha -- 308 polymorphism associated with increased sepsis mortality in ventilated very low birth weight infants**. *Pediatr Infect Dis J* 2004, **23**(5):424-428.
295. Gordon AC, Lagan AL, Aganna E, Cheung L, Peters CJ, McDermott MF, Millo JL, Welsh KI, Holloway P, Hitman GA *et al*: **TNF and TNFR polymorphisms in severe sepsis and septic shock: a prospective multicentre study**. *Genes Immun* 2004, **5**(8):631-640.
296. Fidler KJ, Wilson P, Davies JC, Turner MW, Peters MJ, Klein NJ: **Increased incidence and severity of the systemic inflammatory syndrome in patients deficient in mannose-binding lectin**. *Intensive Care Medicine* 2004, **30**(7):1438-1445.
297. Bessler H, Osovsky M, Sirota L: **Association between IL-1ra gene polymorphism and premature delivery**. *Biol Neonate* 2004, **85**(3):179-183.
298. Barber RC, Aragaki CC, Rivera-Chavez FA, Purdue GF, Hunt JL, Horton JW: **TLR4 and TNF-alpha polymorphisms are associated with an increased risk for severe sepsis following burn injury**. *J Med Genet* 2004, **41**(11):808-813.
299. Ahrens P, Kattner E, Kohler B, Hartel C, Seidenberg J, Segerer H, Moller J, Gopel W: **Mutations of genes involved in the innate immune system as predictors of sepsis in very low birth weight infants**. *Pediatr Res* 2004, **55**(4):652-656.
300. Zhang D, Li J, Jiang ZW, Yu B, Tang X: **Association of two polymorphisms of tumor necrosis factor gene with acute severe pancreatitis**. *J Surg Res* 2003, **112**(2):138-143.
301. Zhang D, Li J, Jiang Z, Yu B, Tang X, Li W: **The relationship between tumor necrosis factor-alpha gene polymorphisms and acute severe pancreatitis**. *Chin Med J (Engl)* 2003, **116**(11):1779-1781.
302. Waterer GW, ElBahlawan L, Quasney MW, Zhang Q, Kessler LA, Wunderink RG: **Heat shock protein 70-2+1267 AA homozygotes have an increased risk of septic shock in adults with community-acquired pneumonia**. *Crit Care Med* 2003, **31**(5):1367-1372.
303. Treszl A, Kocsis I, Szathmari M, Schuler A, Heninger E, Tulassay T, Vasarhelyi B: **Genetic variants of TNF-[FC12]a, IL-1beta, IL-4 receptor [FC12]a-chain, IL-6 and IL-10 genes are not risk factors for sepsis in low-birth-weight infants**. *Biol Neonate* 2003, **83**(4):241-245.
304. Stassen NA, Breit CM, Norfleet LA, Polk HC, Jr.: **IL-18 promoter polymorphisms correlate with the development of post-injury sepsis**. *Surgery* 2003, **134**(2):351-356.
305. Shu Q, Fang X, Chen Q, Stuber F: **IL-10 polymorphism is associated with increased incidence of severe sepsis**. *Chin Med J (Engl)* 2003, **116**(11):1756-1759.
306. Schaaf BM, Boehmke F, Esnaashari H, Seitzer U, Kothe H, Maass M, Zabel P, Dalhoff K: **Pneumococcal septic shock is associated with the interleukin-10-1082 gene promoter polymorphism**. *Am J Respir Crit Care Med* 2003, **168**(4):476-480.
307. Riese J, Woerner K, Zimmermann P, Denzel C, Hohenberger W, Haupt W: **Association of a TNFbeta gene polymorphism with complications after major abdominal operations**. *Shock* 2003, **19**(1):1-4.
308. Lowe PR, Galley HF, Abdel-Fattah A, Webster NR: **Influence of interleukin-10 polymorphisms on interleukin-10 expression and survival in critically ill patients**. *Crit Care Med* 2003, **31**(1):34-38.
309. Harding D, Dhamrait S, Millar A, Humphries S, Marlow N, Whitelaw A, Montgomery H: **Is interleukin-6 - 174 Genotype associated with the development of septicemia in preterm infants?** *Pediatrics* 2003, **112**(4):800-803.
310. Haralambous E, Hibberd ML, Hermans PW, Ninis N, Nadel S, Levin M: **Role of functional plasminogen-activator-inhibitor-1 4G/5G promoter polymorphism in susceptibility, severity, and outcome of meningococcal disease in Caucasian children**. *Crit Care Med* 2003, **31**(12):2788-2793.
311. Garred P, J JS, Quist L, Taaning E, Madsen HO: **Association of mannose-binding lectin polymorphisms with sepsis and fatal outcome, in patients with systemic inflammatory response syndrome**. *J Infect Dis* 2003, **188**(9):1394-1403.
312. Feterowski C, Emmanuilidis K, Miethke T, Gerauer K, Rump M, Ulm K, Holzmann B, Weighardt H: **Effects of functional Toll-like receptor-4 mutations on the immune response to human and experimental sepsis**. *Immunology* 2003, **109**(3):426-431.
313. Calvano JE, Um JY, Agnese DM, Hahm SJ, Kumar A, Coyle SM, Calvano SE, Lowry SF: **Influence of the TNF-alpha and TNF-beta polymorphisms upon infectious risk and outcome in surgical intensive care patients**. *Surg Infect (Larchmt)* 2003, **4**(2):163-169.
314. Barber RC, O'Keefe GE: **Characterization of a single nucleotide polymorphism in the lipopolysaccharide binding protein and its association with sepsis**. *Am J Respir Crit Care Med* 2003, **167**(10):1316-1320.
315. Balding J, Healy CM, Livingstone WJ, White B, Mynett-Johnson L, Cafferkey M, Smith OP: **Genomic polymorphic profiles in an Irish population with meningococcaemia: is it possible to predict severity and outcome of disease?** *Genes Immun* 2003, **4**(8):533-540.
316. Stassen NA, Leslie-Norfleet LA, Robertson AM, Eichenberger MR, Polk HC, Jr.: **Interferon-gamma gene polymorphisms and the development of sepsis in patients with trauma**. *Surgery* 2002, **132**(2):289-292.
317. Schluter B, Raufhake C, Erren M, Schotte H, Kipp F, Rust S, Van Aken H, Assmann G, Berendes E: **Effect of the interleukin-6 promoter polymorphism (-174 G/C) on the incidence and outcome of sepsis**. *Crit Care Med* 2002, **30**(1):32-37.
318. O'Keefe GE, Hybki DL, Munford RS: **The G-->A single nucleotide polymorphism at the -308 position in the tumor necrosis factor-alpha promoter increases the risk for severe sepsis after trauma**. *J Trauma* 2002, **52**(5):817-825; discussion 825-816.
319. Morre SA, Murillo LS, Spaargaren J, Fennema HSA, Pena AS: **Role of the toll-like receptor 4 Asp299Gly polymorphism in susceptibility to Candida albicans infection**. *Journal of Infectious Diseases* 2002, **186**(9):1377-1379.
320. McArthur JA, Zhang Q, Quasney MW: **Association between the A/A genotype at the lymphotoxin-alpha+250 site and increased mortality in children with positive blood cultures**. *Pediatr Crit Care Med* 2002, **3**(4):341-344.
321. Majetschak M, Obertacke U, Schade FU, Bardenheuer M, Voggenreiter G, Bloemeke B, Heesen M: **Tumor necrosis factor gene polymorphisms, leukocyte function, and sepsis susceptibility in blunt trauma patients**. *Clin Diagn Lab Immunol* 2002, **9**(6):1205-1211.
322. Ma P, Chen D, Pan J, Du B: **Genomic polymorphism within interleukin-1 family cytokines influences the outcome of septic patients**. *Crit Care Med* 2002, **30**(5):1046-1050.
323. Lorenz E, Mira JP, Frees KL, Schwartz DA: **Relevance of mutations in the TLR4 receptor in patients with gram-negative septic shock**. *Arch Intern Med* 2002, **162**(9):1028-1032.
324. Kronborg G, Weis N, Madsen HO, Pedersen SS, Wejse C, Nielsen H, Skinhoj P, Garred P: **Variant mannose-binding lectin alleles are not associated with susceptibility to or outcome of invasive pneumococcal infection in randomly included patients**. *J Infect Dis* 2002, **185**(10):1517-1520.
325. Heesen M, Bloemeke B, Schade U, Obertacke U, Majetschak M: **The -260 C-->T promoter polymorphism of the lipopolysaccharide receptor CD14 and severe sepsis in trauma patients**. *Intensive Care Med* 2002, **28**(8):1161-1163.
326. Harding D, Baines PB, Brull D, Vassiliou V, Ellis I, Hart A, Thomson AP, Humphries SE, Montgomery HE: **Severity of meningococcal disease in children and the angiotensin-converting enzyme insertion/deletion polymorphism**. *Am J Respir Crit Care Med* 2002, **165**(8):1103-1106.
327. Gibot S, Cariou A, Drouet L, Rossignol M, Ripoll L: **Association between a genomic polymorphism within the CD14 locus and septic shock susceptibility and mortality rate**. *Crit Care Med* 2002, **30**(5):969-973.
328. Domingo P, Muniz-Diaz E, Baraldes MA, Arilla M, Barquet N, Pericas R, Juarez C, Madoz P, Vazquez G: **Associations between Fc gamma receptor IIA polymorphisms and the risk and prognosis of meningococcal disease**. *American Journal of Medicine* 2002, **112**(1):19-25.
329. Carrol ED, Mobbs KJ, Thomson APJ, Hart CA: **Variable number tandem repeat polymorphism of the interleukin-1 receptor antagonist gene in meningococcal disease [2]**. *Clinical Infectious Diseases* 2002, **35**(4):495-497.
330. Arnalich F, Lopez-Maderuelo D, Codoceo R, Lopez J, Solis-Garrido LM, Capiscol C, Fernandez-Capitan C, Madero R, Montiel C: **Interleukin-1 receptor antagonist gene polymorphism and mortality in patients with severe sepsis**. *Clin Exp Immunol* 2002, **127**(2):331-336.
331. Agnese DM, Calvano JE, Hahm SJ, Coyle SM, Corbett SA, Calvano SE, Lowry SF: **Human toll-like receptor 4 mutations but not CD14 polymorphisms are associated with an increased risk of gram-negative infections**. *J Infect Dis* 2002, **186**(10):1522-1525.
332. Waterer GW, Quasney MW, Cantor RM, Wunderink RG: **Septic shock and respiratory failure in community-acquired pneumonia have different TNF polymorphism associations**. *Am J Respir Crit Care Med* 2001, **163**(7):1599-1604.
333. van der Pol WL, Huizinga TW, Vidarsson G, van der Linden MW, Jansen MD, Keijsers V, de Straat FG, Westerdaal NA, de Winkel JG, Westendorp RG: **Relevance of Fcgamma receptor and interleukin-10 polymorphisms for meningococcal disease**. *J Infect Dis* 2001, **184**(12):1548-1555.
334. Menges T, Hermans PW, Little SG, Langefeld T, Boning O, Engel J, Sluijter M, de Groot R, Hempelmann G: **Plasminogen-activator-inhibitor-1 4G/5G promoter polymorphism and prognosis of severely injured patients**. *Lancet* 2001, **357**(9262):1096-1097.
335. Hubacek JA, Stuber F, Frohlich D, Book M, Wetegrove S, Ritter M, Rothe G, Schmitz G: **Gene variants of the bactericidal/permeability increasing protein and lipopolysaccharide binding protein in sepsis patients: gender-specific genetic predisposition to sepsis**. *Crit Care Med* 2001, **29**(3):557-561.
336. Appoloni O, Dupont E, Vandercruys M, Andriens M, Duchateau J, Vincent JL: **Association of tumor necrosis factor-2 allele with plasma tumor necrosis factor-alpha levels and mortality from septic shock**. *Am J Med* 2001, **110**(6):486-488.
337. Yee AMF, Phan HM, Zuniga R, Salmon JE, Musher DM: **Association between Fc(gamma)RIIa-R131 allotype and bacteremic pneumococcal pneumonia**. *Clinical Infectious Diseases* 2000, **30**(1):25-28.
338. Tang GJ, Huang SL, Yien HW, Chen WS, Chi CW, Wu CW, Lui WY, Chiu JH, Lee TY: **Tumor necrosis factor gene polymorphism and septic shock in surgical infection**. *Crit Care Med* 2000, **28**(8):2733-2736.
339. Lorenz E, Mira JP, Cornish KL, Arbour NC, Schwartz DA: **A novel polymorphism in the toll-like receptor 2 gene and its potential association with staphylococcal infection**. *Infect Immun* 2000, **68**(11):6398-6401.
340. Hubacek JA, Stuber F, Frohlich D, Book M, Wetegrove S, Rothe G, Schmitz G: **The common functional C(-159)T polymorphism within the promoter region of the lipopolysaccharide receptor CD14 is not associated with sepsis development or mortality**. *Genes Immun* 2000, **1**(6):405-407.
341. Westendorp RGJ, Hottenga JJ, Slagboom PE: **Variation in plasminogen-activator-inhibitor-1 gene and risk of meningococcal septic shock**. *Lancet* 1999, **354**(9178):561-563.
342. Schroeder S, Reck M, Hoeft A, Stuber F: **Analysis of two human leukocyte antigen-linked polymorphic heat shock protein 70 genes in patients with severe sepsis**. *Crit Care Med* 1999, **27**(7):1265-1270.
343. Mira JP, Cariou A, Grall F, Delclaux C, Losser MR, Heshmati F, Cheval C, Monchi M, Teboul JL, Riche F *et al*: **Association of TNF2, a TNF-alpha promoter polymorphism, with septic shock susceptibility and mortality: a multicenter study**. *JAMA* 1999, **282**(6):561-568.
344. Majetschak M, Flohe S, Obertacke U, Schroder J, Staubach K, Nast-Kolb D, Schade FU, Stuber F: **Relation of a TNF gene polymorphism to severe sepsis in trauma patients**. *Ann Surg* 1999, **230**(2):207-214.
345. Hermans PW, Hibberd ML, Booy R, Daramola O, Hazelzet JA, de Groot R, Levin M: **4G/5G promoter polymorphism in the plasminogen-activator-inhibitor-1 gene and outcome of meningococcal disease. Meningococcal Research Group**. *Lancet* 1999, **354**(9178):556-560.
346. Flach R, Majetschak M, Heukamp T, Jennissen V, Flohe S, Borgermann J, Obertacke U, Schade FU: **Relation of ex vivo stimulated blood cytokine synthesis to post-traumatic sepsis**. *Cytokine* 1999, **11**(2):173-178.
347. Fang XM, Schroder S, Hoeft A, Stuber F: **Comparison of two polymorphisms of the interleukin-1 gene family: interleukin-1 receptor antagonist polymorphism contributes to susceptibility to severe sepsis**. *Crit Care Med* 1999, **27**(7):1330-1334.
348. Summerfield JA, Sumiya M, Levin M, Turner MW: **Association of mutations in mannose binding protein gene with childhood infection in consecutive hospital series**. *BMJ* 1997, **314**(7089):1229-1232.
349. Bredius RG, Derkx BH, Fijen CA, de Wit TP, de Haas M, Weening RS, van de Winkel JG, Out TA: **Fc gamma receptor IIa (CD32) polymorphism in fulminant meningococcal septic shock in children**. *J Infect Dis* 1994, **170**(4):848-853.
