# Supplementary material for: Host genetic variants in sepsis risk: a field synopsis and meta-analysis
Source: Crit Care. 2019 Jan 25;23:26. doi: 10.1186/s13054-019-2313-0 (PMC6347778; doi:10.1186/s13054-019-2313-0)
Supplement: Supplementary file 3 — Table S2. Characteristics of the genetic variants investigated for associations with the risk of sepsis. (DOCX 70 kb) [file 13054_2019_2313_MOESM3_ESM.docx]

**Table S2. Genetic variant characteristics studied with sepsis risk.**

| **dbSNP ID** | **Alleles** | **Gene Symbol** | **Gene Name** | **Chr** | **SNP Location*** | **Studies** | **Total** |
| --- | --- | --- | --- | --- | --- | --- | --- |
| **PRRs** |  |  |  |  |  |  |  |
| rs5743551 | A/G | TLR1 | Toll like receptor 1 | 4 | 5‘-FR | 4 | 2167 |
| rs5743611 | G/C | TLR1 | Toll like receptor 1 | 4 | Arg80Thr | 2 | 689 |
| rs4833095 | A/G | TLR1 | Toll like receptor 1 | 4 | Asn248Ser | 3 | 1393 |
| rs5743618 | G/T | TLR1 | Toll like receptor 1 | 4 | Ser602Ile | 4 | 2052 |
| rs4696480 | T/A | TLR2 | Toll like receptor 2 | 4 | intron | 3 | 601 |
| rs5743704 | C/A | TLR2 | Toll like receptor 2 | 4 | Pro631His | 1 | 748 |
| rs121917864 | C/T | TLR2 | Toll like receptor 2 | 4 | Arg677Trp | 1 | 422 |
| rs5743708 | G/A | TLR2 | Toll like receptor 2 | 4 | Arg753Gln | 14 | 3916 |
| rs1898830 | A/G | TLR2 | Toll like receptor 2 | 4 | intron | 2 | 1166 |
| rs3804099 | T/C | TLR2 | Toll like receptor 2 | 4 | Asn199Asn | 5 | 1815 |
| rs3804100 | C/T | TLR2 | Toll like receptor 2 | 4 | Ser450Ser | 1 | 433 |
| rs7656411 | T/G | TLR2 | Toll like receptor 2 | 4 | 3'UTR | 1 | 378 |
| rs10116253 | T/C | TLR4 | Toll like receptor 4 | 9 | 5‘-FR | 1 | 303 |
| rs2149356 | G/T | TLR4 | Toll like receptor 4 | 9 | intron | 2 | 898 |
| rs11536879 | A/G | TLR4 | Toll like receptor 4 | 9 | intron | 1 | 754 |
| rs1927907 | C/T | TLR4 | Toll like receptor 4 | 9 | intron | 2 | 1094 |
| rs4986790 | A/G | TLR4 | Toll like receptor 4 | 9 | Asp299Gly | 28 | 7301 |
| rs4986791 | C/T | TLR4 | Toll like receptor 4 | 9 | Thr399Ile | 17 | 4055 |
| rs41426344 | G/C | TLR4 | Toll like receptor 4 | 9 | 3'UTR | 1 | 132 |
| rs10759932 | T/C | TLR4 | Toll like receptor 4 | 9 | 5‘-FR | 1 | 351 |
| rs12377632 | C/T | TLR4 | Toll like receptor 4 | 9 | intron | 1 | 351 |
| rs1153879 | A/G | TLR4 | Toll like receptor 4 | 3 | intron | 1 | 348 |
| rs11536889 | G/C | TLR4 | Toll like receptor 4 | 9 | 3'UTR | 1 | 351 |
| rs7873784 | G/C | TLR4 | Toll like receptor 4 | 9 | 3'UTR | 1 | 350 |
| rs5744168 | C/T | TLR5 | Toll like receptor 5 | 1 | Arg392Stop | 4 | 1155 |
| rs5743810 | C/T | TLR6 | Toll like receptor 6 | 4 | Ser249Pro | 1 | 274 |
| rs187084 | A/G | TLR9 | Toll like receptor 9 | 3 | 5‘-FR | 4 | 1131 |
| rs5743836 | T/C | TLR9 | Toll like receptor 9 | 3 | 5‘-FR | 2 | 317 |
| rs352139 | A/G | TLR9 | Toll like receptor 9 | 3 | intron | 1 | 551 |
| rs352140 | C/T | TLR9 | Toll like receptor 9 | 3 | Pro545Pro | 4 | 1807 |
| rs352162 | T/C | TLR9 | Toll like receptor 9 | 3 | 3'-FR | 1 | 547 |
| 63bp ins/del | ins/del | RAGE | Advanced glycosylation end-product specific receptor | 6 | 5‘-FR | 2 | 728 |
| rs2070600 | G/A | RAGE | Advanced glycosylation end-product specific receptor | 6 | Gly68Ser | 3 | 1500 |
| rs1800624 | T/A | RAGE | Advanced glycosylation end-product specific receptor | 6 | 5‘-FR | 3 | 1500 |
| rs1800625 | T/C | RAGE | Advanced glycosylation end-product specific receptor | 6 | 5‘-FR | 4 | 2316 |
| rs7768162 | C/T | TREM-1 | Triggering receptor expressed on myeloid cells 1 | 6 | 5‘-FR | 1 | 314 |
| rs9471535 | A/G | TREM-1 | Triggering receptor expressed on myeloid cells 1 | 6 | 5‘-FR | 1 | 314 |
| rs2234237 | T/A | TREM-1 | Triggering receptor expressed on myeloid cells 1 | 6 | Ser25Thr | 3 | 747 |
| rs2234246 | G/A | TREM-1 | Triggering receptor expressed on myeloid cells 1 | 6 | intron | 2 | 434 |
| rs6958571 | T/GG ins/del | NOD1 | Nucleotide binding oligomerization domain containing 1 | 7 | intron | 1 | 750 |
| rs2066844 | C/T | NOD2 | Nucleotide binding oligomerization domain containing 2 | 16 | Arg702Trp | 3 | 1172 |
| rs2066845 | G/C | NOD2 | Nucleotide binding oligomerization domain containing 2 | 16 | Gly908Arg | 3 | 1173 |
| rs2066847 | -/C ins/del | NOD2 | Nucleotide binding oligomerization domain containing 2 | 16 | Leu1007Pro | 5 | 1955 |
| rs2027432 | G/A | NLRP3 | NLR family pyrin domain containing 3 | 1 | 5‘-FR | 1 | 718 |
| rs12048215 | A/G | NLRP3 | NLR family pyrin domain containing 3 | 1 | intron | 1 | 718 |
| rs35829419 | C/A | NLRP3 | NLR family pyrin domain containing 3 | 1 | Gln705Lys | 1 | 1063 |
| rs41423247 | C/G | GR | Glutathione reductase | 3 | intron | 1 | 95 |
| rs2043211 | T/A | CARD8 | Caspase recruitment domain family member 8 | 19 | Cys10Ter | 1 | 1063 |
| rs4077515 | G/A | CARD9 | Caspase recruitment domain family member 9 | 9 | Ser12Asn | 2 | 672 |
| rs2569191 | G/A | CD14 | CD14 molecule | 5 | intron | 1 | 105 |
| rs2569190 | G/A | CD14 | CD14 molecule | 5 | intron | 19 | 4270 |
| rs2563298 | C/A | CD14 | CD14 molecule | 5 | intron | 1 | 350 |
| rs11465996 | C/G | LY96/MD2 | Lymphocyte antigen 96 | 8 | 5‘-FR | 2 | 726 |
| rs7843858 | G/A | LY96/MD2 | Lymphocyte antigen 96 | 8 | 5‘-FR | 2 | 726 |
| rs2114169 | A/T | LY96/MD2 | Lymphocyte antigen 96 | 8 | intron | 2 | 726 |
| rs1780616 | C/T | LBP | Lipopolysaccharide binding protein | 20 | 5‘-FR | 3 | 1286 |
| rs5741812 | A/T | LBP | Lipopolysaccharide binding protein | 20 | 5‘-FR | 1 | 532 |
| rs2232582 | T/C | LBP | Lipopolysaccharide binding protein | 20 | Pro97Pro | 2 | 452 |
| 292T/G | T/G | LBP | Lipopolysaccharide binding protein | 20 | Cys98Gly | 1 | 454 |
| rs2232618 | T/C | LBP | Lipopolysaccharide binding protein | 20 | Phe436Leu | 4 | 1882 |
| rs7096206 | G/C | MBL2 | Mannose binding lectin 2 | 10 | 5‘-FR | 15 | 7237 |
| rs11003125 | G/C | MBL2 | Mannose binding lectin 2 | 10 | 5‘-FR | 4 | 1230 |
| rs5030737 | C/T | MBL2 | Mannose binding lectin 2 | 10 | Arg52Cys | 1 | 433 |
| rs1800450 | G/A | MBL2 | Mannose binding lectin 2 | 10 | Gly54Asp | 5 | 1182 |
| rs1800451 | G/A | MBL2 | Mannose binding lectin 2 | 10 | Gly57Glu | 2 | 514 |
| rs7095891 | T/C | MBL2 | Mannose binding lectin 2 | 10 | intron | 1 | 95 |
| rs5030737,  rs1800450,  rs1800451 | A/O haplotype# | MBL2 | Mannose binding lectin 2 | 10 | Asp52Cys,  Gly54Asp,  Gly57Glu | 23 | 9066 |
| rs72550870 | A/G | MASP2 | Mannan binding lectin serine peptidase 2 | 1 | Asp105Gly | 7 | 3489 |
| rs56392418 | A/G | MASP2 | Mannan binding lectin serine peptidase 2 | 1 | Pro111Leu | 1 | 240 |
| rs12711521 | G/T | MASP2 | Mannan binding lectin serine peptidase 2 | 1 | Asp371Tyr | 1 | 219 |
| rs2273346 | C/T | MASP2 | Mannan binding lectin serine peptidase 2 | 1 | Val377Ala | 1 | 751 |
| rs5743507 | G/C | BPI | Bactericidal permeability increasing protein | 20 | Val182Val | 3 | 1798 |
| rs4358188 | A/G | BPI | Bactericidal permeability increasing protein | 20 | Lys216Glu | 4 | 1886 |
| intron5 T/C PstI | T/C | BPI | Bactericidal permeability increasing protein | 20 | intron | 1 | 454 |
| **Signaling molecules** | |  |  |  |  |  |  |
| rs1059703 | T/C | IRAK1 | Interleukin 1 receptor associated kinase 1 | X | Leu532Ser | 3 | 2131 |
| rs1059702 | T/C | IRAK1 | Interleukin 1 receptor associated kinase 1 | X | Phe196Ser | 1 | 515 |
| rs4251545 | G/A | IRAK-4 | Interleukin 1 receptor associated kinase 4 | 12 | Ala304Thr | 1 | 384 |
| rs4251533 | A/G | IRAK-4 | Interleukin 1 receptor associated kinase 4 | 12 | intron | 1 | 382 |
| rs4251481 | A/G | IRAK-4 | Interleukin 1 receptor associated kinase 4 | 12 | intron | 1 | 384 |
| rs4238087 | A/G | IRAK-4 | Interleukin 1 receptor associated kinase 4 | 12 | intron | 1 | 380 |
| rs4251431 | G/T | IRAK-4 | Interleukin 1 receptor associated kinase 4 | 12 | intron | 1 | 382 |
| rs1461567 | C/T | IRAK-4 | Interleukin 1 receptor associated kinase 4 | 12 | intron | 1 | 378 |
| rs4251513 | C/G | IRAK-4 | Interleukin 1 receptor associated kinase 4 | 12 | intron | 1 | 378 |
| rs5743867 | T/C | TOLLIP | Toll interacting protein | 11 | intron | 2 | 780 |
| rs3750920 | G/A | TOLLIP | Toll interacting protein | 11 | intron | 1 | 756 |
| rs3793964 | A/G | TOLLIP | Toll interacting protein | 11 | intron | 1 | 755 |
| rs3793963 | G/A | TOLLIP | Toll interacting protein | 11 | intron | 1 | 754 |
| rs5744002 | G/A | TOLLIP | Toll interacting protein | 11 | intron | 1 | 753 |
| rs5743942 | T/C | TOLLIP | Toll interacting protein | 11 | intron | 1 | 759 |
| rs5743944 | G/A | TOLLIP | Toll interacting protein | 11 | intron | 1 | 755 |
| rs5743947 | G/A | TOLLIP | Toll interacting protein | 11 | intron | 1 | 753 |
| rs595209 | C/A | TIRAP | TIR domain containing adaptor protein | 11 | intron | 1 | 577 |
| rs3802813 | G/A | TIRAP | TIR domain containing adaptor protein | 11 | Ser55Asn | 1 | 576 |
| rs8177374 | C/T | TIRAP | TIR domain containing adaptor protein | 11 | Ser180Leu | 6 | 3515 |
| rs7932766 | C/T | TIRAP | TIR domain containing adaptor protein | 11 | Ala186Ala | 1 | 576 |
| rs8177375 | A/G | TIRAP | TIR domain containing adaptor protein | 11 | intron | 1 | 579 |
| rs4988453 | C/G | MyD 88 | Myeloid differentiation primary response gene 88 | 3 | 5’UTR | 1 | 130 |
| rs4988457 | C/G | MyD 88 | Myeloid differentiation primary response gene 88 | 3 | Intron | 1 | 130 |
| rs7744 | A/G | MyD88 | Myeloid differentiation primary response gene 88 | 3 | 3'UTR | 1 | 754 |
| rs6853 | A/G | MyD88 | Myeloid differentiation primary response gene 88 | 3 | 3'UTR | 1 | 755 |
| **Transcription factors** | |  |  |  |  |  |  |
| rs28362491 | ATTG ins/del | NFKB1 | Nuclear factor kappa B subunit 1 | 4 | intron | 3 | 1337 |
| rs4648068 | A/G | NFKB1 | Nuclear factor kappa B subunit 1 | 4 | intron | 1 | 752 |
| rs3138053 | A/G | NFKBIA | NFKB inhibitor alpha | 14 | 5‘-FR | 1 | 407 |
| rs2071592 | A/T | NFKBIL1 | NFKB inhibitor like 1 | 6 | intron | 1 | 1464 |
| rs10448143 | C/T | NFKBIL2 | NFKB inhibitor like 2 | 8 | 5‘-FR | 1 | 1137 |
| rs2170096 | C/G | NFKBIL2 | NFKB inhibitor like 2 | 8 | 5‘-FR | 1 | 565 |
| rs4925858 | G/A | NFKBIL2 | NFKB inhibitor like 2 | 8 | 5‘-FR | 1 | 1237 |
| rs760477 | C/T | NFKBIL2 | NFKB inhibitor like 2 | 8 | intron | 1 | 1159 |
| rs2306384 | A/G | NFKBIL2 | NFKB inhibitor like 2 | 8 | Ser493Gly | 1 | 976 |
| rs4082353 | G/T | NFKBIL2 | NFKB inhibitor like 2 | 8 | intron | 1 | 576 |
| rs7119750 | C/T | RELA | RELA proto-oncogene, NF-kB subunit | 11 | intron | 1 | 648 |
| rs842647 | G/A | REL | REL proto-oncogene, NF-kB subunit | 2 | intron | 2 | 2628 |
| rs13031237 | T/A | REL | REL proto-oncogene, NF-kB subunit | 2 | intron | 1 | 1895 |
| rs135551 | G/A | PPARA | Peroxisome proliferator activated receptor alpha | 22 | intron | 1 | 734 |
| rs5769178 | A/C | PPARA | Peroxisome proliferator activated receptor alpha | 22 | intron | 1 | 734 |
| rs4253711 | G/A | PPARA | Peroxisome proliferator activated receptor alpha | 22 | intron | 1 | 734 |
| rs4823613 | A/G | PPARA | Peroxisome proliferator activated receptor alpha | 22 | intron | 1 | 734 |
| rs6902123 | T/C | PPARD | Peroxisome proliferator activated receptor delta | 6 | intron | 1 | 734 |
| rs2016520 | T/C | PPARD | Peroxisome proliferator activated receptor delta | 6 | intron | 1 | 734 |
| rs4684846 | G/A | PPARG | Peroxisome proliferator activated receptor gamma | 3 | intron | 1 | 727 |
| rs10865710 | C/G | PPARG | Peroxisome proliferator activated receptor gamma | 3 | intron | 1 | 734 |
| rs1822825 | G/A | PPARG | Peroxisome proliferator activated receptor gamma | 3 | intron | 1 | 732 |
| rs1801282 | C/G | PPARG | Peroxisome proliferator activated receptor gamma | 3 | Pro12Ala | 2 | 1259 |
| rs2972164 | C/T | PPARG | Peroxisome proliferator activated receptor gamma | 3 | intron | 1 | 606 |
| **Cytokines** |  |  |  |  |  |  |  |
| rs1799964 | T/C | TNFA | Tumor necrosis factor alpha | 6 | 3‘-FR | 2 | 1771 |
| rs1800630 | C/A | TNFA | Tumor necrosis factor alpha | 6 | 3‘-FR | 4 | 3064 |
| rs1799724 | C/T | TNFA | Tumor necrosis factor alpha | 6 | 3‘-FR | 3 | 1875 |
| rs1800750 | G/A | TNFA | Tumor necrosis factor alpha | 6 | 5‘-FR | 3 | 605 |
| rs1800629 | G/A | TNFA | Tumor necrosis factor alpha | 6 | 5‘-FR | 41 | 15057 |
| rs361525 | G/A | TNFA | Tumor necrosis factor alpha | 6 | 5‘-FR | 9 | 4384 |
| rs1800610 | G/A | TNFA | Tumor necrosis factor alpha | 6 | intron | 1 | 284 |
| rs909253 | T/C | LTA/TNFB | Lymphotoxin alpha | 6 | intron | 32 | 12185 |
| 365G/C | G/C | LTA/TNFB | Lymphotoxin alpha | 6 | intron | 1 | 554 |
| rs2229094 | T/C | LTA/TNFB | Lymphotoxin alpha | 6 | Cys13Arg | 1 | 147 |
| rs4149570 | G/T | TNFRSF1A | TNF receptor superfamily member 1A | 12 | 5‘-FR | 2 | 1355 |
| rs767455 | A/G | TNFRSF1A | TNF receptor superfamily member 1A | 12 | Pro12Pro | 2 | 1355 |
| 1135C/T | C/T | TNFRSF1A | TNF receptor superfamily member 1A | 1 | intron | 1 | 340 |
| rs1061622 | T/G | TNFRSF1B | TNF receptor superfamily member 1B | 1 | Met196Arg | 4 | 3284 |
| rs3397 | G/A | TNFRSF1B | TNF receptor superfamily member 1B | 1 | 3'UTR | 1 | 1428 |
| rs34557412 | A/G | TNFRSF13B | TNF receptor superfamily member 13B | 17 | Cys104Arg | 1 | 215 |
| rs77874543 | C/G | TNFRSF13C | TNF receptor superfamily member 13C | 22 | Pro21Arg | 1 | 215 |
| rs61756766 | C/T | TNFRSF13C | TNF receptor superfamily member 13C | 22 | His159Tyr | 1 | 215 |
| rs1800587 | C/T | IL1A | Interleukin 1 alpha | 2 | 5'UTR | 3 | 500 |
| rs1143623 | G/C | IL1B | Interleukin 1 beta | 2 | 5‘-FR | 1 | 308 |
| rs16944 | G/A | IL1B | Interleukin 1 beta | 2 | 5‘-FR | 9 | 3195 |
| rs1143627 | C/T | IL1B | Interleukin 1 beta | 2 | 5'UTR | 6 | 2158 |
| rs1143643 | C/T | IL1B | Interleukin 1 beta | 2 | intron | 1 | 288 |
| rs143634 | C/T | IL1B | Interleukin 1 beta | 2 | intron | 8 | 2332 |
| VNTR | A2-/+ repeat | IL1RN | Interleukin 1 receptor antagonist | 2 | intron | 12 | 3297 |
| -330T/G | T/G | IL2 | Interleukin 2 | 2 | 5‘-FR | 1 | 110 |
| rs2243250 | T/C | IL4 | Interleukin 4 | 5 | 5‘-FR | 4 | 1561 |
| rs2243248 | T/G | IL4 | Interleukin 4 | 5 | 5‘-FR | 2 | 861 |
| rs1801275 | A/G | IL4RA | Interleukin 4 receptor | 5 | Gln576Arg | 1 | 68 |
| rs2069812 | C/T | IL5 | Interleukin 5 | 5 | intron | 1 | 748 |
| rs1548216 | G/C | IL6 | Interleukin 6 | 7 | intron | 1 | 302 |
| rs2069840 | C/G | IL6 | Interleukin 6 | 7 | intron | 1 | 302 |
| rs1800796 | G/C | IL6 | Interleukin 6 | 7 | intron | 6 | 1771 |
| rs1800797 | G/A | IL6 | Interleukin 6 | 7 | intron | 2 | 826 |
| rs1800795 | G/C | IL6 | Interleukin 6 | 7 | intron | 25 | 8630 |
| 48892A/C | A/C | IL6R | Interleukin 6 receptor | 7 | intron | 1 | 110 |
| rs4073 | T/A | IL8/CXCL8 | C-X-C motif chemokine ligand 8 | 4 | 5‘-FR | 12 | 3502 |
| rs2227306 | C/T | IL8/CXCL8 | C-X-C motif chemokine ligand 8 | 4 | intron | 1 | 351 |
| rs1126647 | A/T | IL8/CXCL8 | C-X-C motif chemokine ligand 8 | 4 | 3'UTR | 1 | 347 |
| rs1800872 | C/A | IL10 | Interleukin 10 | 1 | 5‘-FR | 12 | 3852 |
| rs1800871 | C/T | IL10 | Interleukin 10 | 1 | 5‘-FR | 13 | 4013 |
| rs1800896 | A/G | IL10 | Interleukin 10 | 1 | 5‘-FR | 24 | 6067 |
| rs1800890 | T/A | IL10 | Interleukin 10 | 1 | 5‘-FR | 1 | 750 |
| rs41292470 | ins/del | IL12B | Interleukin 12B | 15 | intron | 2 | 689 |
| rs3212227 | A/C | IL12B | Interleukin 12B | 5 | 3'UTR | 3 | 857 |
| rs17860508 | CTCTAA/GC | IL12B | Interleukin 12B | 5 | 5‘-FR | 1 | 168 |
| rs17882232 | C/T | IL12RB1 | Interleukin 12 receptor subunit beta 1 | 19 | 5'UTR | 2 | 689 |
| rs11575934 | A/G | IL12RB1 | Interleukin 12 receptor subunit beta 1 | 19 | Gln214Arg | 2 | 689 |
| rs763780 | T/C | IL17 | Interleukin 17 | 6 | His161Arg | 1 | 42 |
| rs187238 | G/C | IL18 | Interleukin 18 | 11 | 5‘-FR | 5 | 893 |
| rs549908 | T/G | IL18 | Interleukin 18 | 11 | Ser35Ser | 2 | 689 |
| rs1946518 | C/A | IL18 | Interleukin 18 | 11 | 5‘-FR | 2 | 135 |
| rs907715 | C/T | IL21 | Interleukin 21 | 4 | intron | 1 | 414 |
| rs2055979 | G/T | IL21 | Interleukin 21 | 4 | intron | 1 | 414 |
| rs12508721 | C/T | IL21 | Interleukin 21 | 4 | intron | 1 | 414 |
| rs153109 | A/G | IL-27 | Interleukin 27 | 16 | intron | 2 | 1986 |
| rs17855750 | T/G | IL-27 | Interleukin 27 | 16 | Ser61Thr | 1 | 803 |
| rs2430561 | A/T | IFNG | Interferon gamma | 12 | intron | 4 | 1474 |
| rs2069705 | C/T | IFNG | Interferon gamma | 12 | 5‘-FR | 1 | 409 |
| rs2069718 | T/C | IFNG | Interferon gamma | 12 | intron | 1 | 409 |
| (CA) repeat | 12 repeat | IFNG | Interferon gamma | 12 | intron | 1 | 61 |
| rs755622 | G/C | MIF | Macrophage migration inhibitory factor | 22 | 5‘-FR | 4 | 807 |
| rs1412125 | T/C | HMGB1 | High mobility group box 1 | 13 | intron | 1 | 555 |
| rs2249825 | C/G | HMGB1 | High mobility group box 1 | 13 | intron | 2 | 1246 |
| rs1045411 | G/A | HMGB1 | High mobility group box 1 | 13 | 3'UTR | 2 | 1246 |
| rs1060348 | C/T | HMGB1 | High mobility group box 1 | 13 | Phe103Phe | 1 | 81 |
| rs1360485 | A/G | HMGB1 | High mobility group box 1 | 13 | 3‘-FR | 1 | 690 |
| rs1024611 | C/T | MCP-1 | Macrophage cationic peptide 1 | 17 | 5‘-FR | 1 | 803 |
| rs2857656 | A/C | MCP-1 | Macrophage cationic peptide 1 | 17 | 5‘-FR | 1 | 803 |
| rs3117604 | C/T | CXCL1 | C-X-C motif chemokine ligand 1 | 4 | 5‘-FR | 3 | 1698 |
| rs4074 | A/G | CXCL1 | C-X-C motif chemokine ligand 1 | 4 | intron | 3 | 1697 |
| rs1429638 | A/C | CXCL1 | C-X-C motif chemokine ligand 1 | 4 | 3‘-FR | 3 | 1697 |
| rs3806792 | C/T | CXCL2 | C-X-C motif chemokine ligand 2 | 4 | 5‘-FR | 1 | 720 |
| rs4256246 | A/G | CXCL10 | C-X-C motif chemokine ligand 10 | 4 | 5‘-FR | 1 | 732 |
| rs4508917 | A/G | CXCL10 | C-X-C motif chemokine ligand 10 | 4 | 5‘-FR | 1 | 732 |
| rs1065297 | C/T | CXCL12 | C-X-C motif chemokine ligand 12 | 10 | 3’UTR | 1 | 726 |
| rs1029153 | C/T | CXCL12 | C-X-C motif chemokine ligand 12 | 10 | 3’UTR | 1 | 731 |
| rs1801157 | A/G | CXCL12 | C-X-C motif chemokine ligand 12 | 10 | 3’UTR | 1 | 732 |
| rs266087 | A/G | CXCL12 | C-X-C motif chemokine ligand 12 | 10 | intron | 3 | 1698 |
| rs2297630 | A/G | CXCL12 | C-X-C motif chemokine ligand 12 | 10 | intron | 3 | 1698 |
| rs2839693 | A/G | CXCL12 | C-X-C motif chemokine ligand 12 | 10 | intron | 3 | 1692 |
| rs1413519 | C/G | CXCL12 | C-X-C motif chemokine ligand 12 | 10 | 5‘-FR | 1 | 732 |
| rs3806792 | G/A | CXCR2 | C-X-C motif chemokine receptor 2 | 4 | 5‘-FR | 1 | 535 |
| rs10682383 | (AC) 24±1 repeat | CXCR2 | C-X-C motif chemokine receptor 2 | 4 | 5‘-FR | 1 | 535 |
| rs414171 | A/T | CISH | Cytokine inducible SH2 containing protein | 3 | 5'UTR | 2 | 1086 |
| rs2239751 | T/G | CISH | Cytokine inducible SH2 containing protein | 3 | intron | 1 | 806 |
| rs10777530 | C/T | SOCS2 | Suppressor of cytokine signaling 2 | 12 | 5‘-FR | 1 | 806 |
| rs8064821 | A/C | SOCS3 | Suppressor of cytokine signaling 3 | 17 | 5‘-FR | 1 | 806 |
| rs1209087 | T/C | SOCS4 | Suppressor of cytokine signaling 4 | 14 | 5‘-FR | 1 | 806 |
| rs3829835 | A/G | SOCS5 | Suppressor of cytokine signaling 5 | 2 | 5‘-FR | 1 | 804 |
| rs17771255 | C/T | SOCS5 | Suppressor of cytokine signaling 6 | 2 | intron | 1 | 806 |
| rs3768720 | C/A | SOCS5 | Suppressor of cytokine signaling 5 | 2 | 3'UTR | 1 | 806 |
| rs1351887 | A/T | SOCS6 | Suppressor of cytokine signaling 6 | 18 | 5‘-FR | 1 | 806 |
| rs9646604 | A/G | SOCS6 | Suppressor of cytokine signaling 6 | 18 | intron | 1 | 806 |
| rs3809954 | A/G | SOCS6 | Suppressor of cytokine signaling 6 | 18 | 3'UTR | 1 | 806 |
| rs3748726 | A/G | SOCS7 | Suppressor of cytokine signaling 7 | 17 | 3'UTR | 1 | 806 |
| rs3091244 | C/A | CRP | C-reactive protein | 1 | 5‘-FR | 1 | 69 |
| rs2808530 | C/T | CRP | C-reactive protein | 9 | intron | 1 | 69 |
| rs1205 | C/T | CRP | C-reactive protein | 1 | 3'UTR | 1 | 69 |
| **Coagulation factors** | |  |  |  |  |  |  |
| 20210G/A | G/A | F2 | Coagulation factor II | 11 | intron | 1 | 1006 |
| rs6025 | G/A | F5 | Coagulation factor V | 1 | Arg506Gln | 5 | 18358 |
| 323del/ins | 323del/ins | F7 | Coagulation factor VII | 13 | intron | 1 | 1004 |
| rs5985 | C/A | F13 | Coagulation factor XIII | 6 | Val34Leu | 1 | 1008 |
| (GT)n repeats | ≥25/<25 | HMOX1 | Heme oxygenase 1 | 22 | 5‘-FR | 1 | 118 |
| rs1799810 | A/T | PROC | Protein C | 2 | 5‘-FR | 1 | 223 |
| rs1799809 | A/G | PROC | Protein C | 2 | 5‘-FR | 2 | 786 |
| rs1799808 | C/T | PROC | Protein C | 2 | 5‘-FR | 2 | 786 |
| rs2069940 | C/G | EPCR | Protein C receptor | 20 | intron | 1 | 389 |
| rs2069952 | T/C | EPCR | Protein C receptor | 20 | intron | 1 | 389 |
| rs867186 | A/G | EPCR | Protein C receptor | 20 | Ser219Gly | 1 | 388 |
| 23bp del/ins | ins/del | EPCR | Protein C receptor | 20 | 5'UTR | 1 | 205 |
| rs2069948 | C/T | EPCR | Protein C receptor | 20 | intron | 1 | 177 |
| rs867186 | C/T | EPCR | Protein C receptor | 20 | intron | 1 | 177 |
| 79G/A | G/A | Protein Z | Protein Z | 20 | intron | 1 | 123 |
| rs1801274 | A/G | FCGR2A | Fc fragment of IgG receptor IIa | 1 | His131Arg | 9 | 2579 |
| 559G/A | G/A | FCGR3A | Fc fragment of IgG receptor IIIa | 1 | Phe158Val | 1 | 377 |
| NA1/2 | 4aa substitutions | FCGR3B | Fc fragment of IgG receptor IIIb | 1 | - | 2 | 3773 |
| rs1799768 | 5G/4G | SPERPINE1 | Serpin family E member 1 | 7 | 5‘-FR | 11 | 3302 |
| rs7242 | T/G | SPERPINE1 | Serpin family E member 1 | 7 | 3'UTR | 1 | 79 |
| rs4646994 | ins/del | ACE | Angiotensin I converting enzyme | 17 | intron | 9 | 4246 |
| rs4291 | A/C | ACE | Angiotensin I converting enzyme | 17 | 5‘-FR | 1 | 480 |
| rs11684747 | A/G | ADAM17 | ADAM metallopeptidase domain 17 | 2 | 5‘-FR | 1 | 770 |
| rs1524668 | A/C | ADAM17 | ADAM metallopeptidase domain 17 | 2 | 5‘-FR | 1 | 770 |
| rs11689958 | A/G | ADAM17 | ADAM metallopeptidase domain 17 | 2 | 5‘-FR | 1 | 770 |
| rs55790676 | A/C | ADAM17 | ADAM metallopeptidase domain 17 | 2 | 5'UTR | 1 | 770 |
| rs12692386 | A/G | ADAM17 | ADAM metallopeptidase domain 17 | 2 | 5'UTR | 1 | 770 |
| APOE3 haplotype | -/+ | APOE | Apolipoprotein E | 19 | Cys112Arg, Cys158Arg | 1 | 343 |
| rs1704/ rs371194629 | 14bp ins/del | HLA-G | Major histocompatibility complex, class I, G | 6 | 3'UTR | 2 | 892 |
| rs1707 | T/C | HLA-G | Major histocompatibility complex, class I, G | 6 | 3'UTR | 2 | 892 |
| rs1710 | G/C | HLA-G | Major histocompatibility complex, class I, G | 6 | 3'UTR | 2 | 892 |
| rs17179101 | C/A | HLA-G | Major histocompatibility complex, class I, G | 6 | 3'UTR | 2 | 892 |
| rs17179108 | C/T | HLA-G | Major histocompatibility complex, class I, G | 6 | 3'UTR | 2 | 892 |
| rs1063320 | C/G | HLA-G | Major histocompatibility complex, class I, G | 6 | 3'UTR | 2 | 892 |
| rs9380142 | G/A | HLA-G | Major histocompatibility complex, class I, G | 6 | 3'UTR | 2 | 892 |
| rs1610696 | C/G | HLA-G | Major histocompatibility complex, class I, G | 6 | 3'UTR | 1 | 254 |
| rs1324214 | C/T | TF | Tissue factor | 1 | intron | 1 | 1032 |
| rs762484 | T/C | TF | Tissue factor | 1 | intron | 1 | 1032 |
| rs696619 | A/G | TF | Tissue factor | 1 | intron | 1 | 1023 |
| rs3917615 | C/T | TF | Tissue factor | 1 | intron | 1 | 1032 |
| rs1361600 | A/G | TF | Tissue factor | 1 | 5‘-FR | 1 | 1040 |
| rs958587 | C/T | TF | Tissue factor | 1 | 5‘-FR | 1 | 1035 |
| rs3917643 | A/G | TF | Tissue factor | 1 | intron | 1 | 1044 |
| rs145977586 | G/A | TF | Tissue factor | 1 | Ile264Met | 1 | 1044 |
| rs3755248 | T/C | TFPI | Tissue factor pathway inhibitor | 2 | intron | 1 | 1030 |
| rs3213739 | G/T | TFPI | Tissue factor pathway inhibitor | 2 | intron | 1 | 1036 |
| rs7594359 | C/T | TFPI | Tissue factor pathway inhibitor | 2 | intron | 1 | 1025 |
| rs10931292 | T/C | TFPI | Tissue factor pathway inhibitor | 2 | 5‘-FR | 1 | 1031 |
| rs8176441 | T/C | TFPI | Tissue factor pathway inhibitor | 2 | intron | 1 | 1032 |
| rs12613071 | T/C | TFPI | Tissue factor pathway inhibitor | 2 | intron | 1 | 1030 |
| rs10153820 | G/A | TFPI | Tissue factor pathway inhibitor | 2 | 5‘-FR | 1 | 1027 |
| rs8176592 | T/C | TFPI | Tissue factor pathway inhibitor | 2 | intron | 1 | 1041 |
| rs2192824 | C/T | TFPI | Tissue factor pathway inhibitor | 2 | intron | 1 | 1031 |
| rs668 | C/G | PECAM1 | Platelet/endothelial cell adhesion molecule 1 | 17 | Leu125Val | 1 | 307 |
| 1166A/C | A/C | ATR-1 | angiotensin II type 1 receptor | 3 | 5‘UTR | 2 | 3269 |
| rs2070744 | T/C | NOS3 | Nitric oxide synthase 3 | 7 | intron | 2 | 396 |
| rs1799983 | G/T | NOS3 | Nitric oxide synthase 3 | 7 | Glu298Asp | 2 | 398 |
| intron4 VNTR | 5/4 repeats | NOS3 | Nitric oxide synthase 3 | 7 | intron | 2 | 415 |
| exon22 G/A | G/A | NOS2 | Nitric oxide synthase 2 | 11 | Thr/Thr | 1 | 180 |
| 6G/7G ins/del | 6G/7G | DDAH2 | Dimethylarginine dimethylaminohydrolase 2 | 6 | intron | 1 | 56 |
| rs805305 | G/C | DDAH2 | Dimethylarginine dimethylaminohydrolase 2 | 6 | intron | 1 | 82 |
| **Complement molecules** | |  |  |  |  |  |  |
| rs17549193 | C/T | FCN2 | Ficolin 2 | 9 | Thr236Met | 1 | 219 |
| rs7851696 | G/T | FCN2 | Ficolin 2 | 9 | Ala258Ser | 1 | 219 |
| **Others** |  |  |  |  |  |  |  |
| rs2227956 | C/T | HSPA1L | Heat shock protein family A member 1 like | 6 | Thr493Arg | 2 | 840 |
| rs740598 | G/A | HSPA12A | Heat shock protein family A | 10 | intron | 4 | 769 |
| rs10948128 | G/C | HSP90AB1 | Heat shock protein 90 alpha family class B member 1 | 6 | intron | 1 | 142 |
| rs9472238 | C/A | HSP90AB1 | Heat shock protein 90 alpha family class B member 1 | 6 | intron | 1 | 142 |
| rs324131 | T/C | HSP90AB1 | Heat shock protein 90 alpha family class B member 1 | 6 | intron | 1 | 142 |
| rs497116 | C/T | CASP12 | Caspase 12 | 11 | Arg125Stop | 2 | 366 |
| 24bp duplication | ins/del | CHIT1 | Chitinase 1 | 15 | intron | 1 | 190 |
| rs3087454 | G/T | CHRNA7 | Cholinergic receptor, nicotinic, alpha polypeptide 7 | 15 | intron | 1 | 496 |
| rs6494165 | T/G | CHRNA7 | Cholinergic receptor, nicotinic, alpha polypeptide 7 | 15 | intron | 1 | 466 |
| rs3826029 | A/G | CHRNA7 | Cholinergic receptor, nicotinic, alpha polypeptide 7 | 15 | intron | 1 | 496 |
| rs28531779 | G/C | CHRNA7 | Cholinergic receptor, nicotinic, alpha polypeptide 7 | 15 | intron | 1 | 496 |
| rs11292517 | 1G/2G | MMP1 | Matrix metallopeptidase 1 | 11 | intron | 1 | 180 |
| rs5854 | T/C | MMP1 | Matrix metallopeptidase 1 | 11 | intron | 1 | 210 |
| rs2397776 | C/T | MMP1 | Matrix metallopeptidase 1 | 11 | intron | 1 | 210 |
| rs470747 | C/T | MMP1 | Matrix metallopeptidase 1 | 11 | intron | 1 | 209 |
| rs35068180 | 5A/6A | MMP3 | Matrix metallopeptidase 3 | 11 | 5‘-FR | 1 | 181 |
| rs11225395 | C/T | MMP8 | Matrix metallopeptidase 8 | 11 | 5‘-FR | 1 | 175 |
| rs34016235 | C/T | MMP9 | Matrix metallopeptidase 9 | 20 | 5‘-FR | 1 | 180 |
| rs2252070 | A/G | MMP13 | Matrix metallopeptidase 13 | 11 | 5‘-FR | 1 | 181 |
| rs2664349 | A/G | MMP16 | Matrix metallopeptidase 16 | 8 | intron | 2 | 369 |
| rs820336 | A/G | MYLK | Myosin light chain kinase | 3 | intron | 3 | 385 |
| rs33264 | G/C | MYLK | Myosin light chain kinase | 3 | intron | 1 | 81 |
| rs820325 | A/G | MYLK | Myosin light chain kinase | 3 | intron | 1 | 82 |
| rs443198 | C/T | NOTCH4 | Notch 4 | 6 | Gly111Gly | 1 | 1435 |
| rs520803 | A/C | NOTCH4 | Notch 4 | 6 | Gln284His | 1 | 1509 |
| rs760293 | C/T | BAG6 | BCL2 associated athanogene 6 | 6 | intron | 1 | 1417 |
| rs2256965 | C/T | LST1 | Leukocyte specific transcript 1 | 6 | intron | 1 | 1424 |
| rs130066 | C/G | CCHCR1 | Coiled-coil alpha-helical rod protein 1 | 6 | Arg164Ser | 1 | 1541 |
| rs1576 | C/G | CCHCR1 | Coiled-coil alpha-helical rod protein 1 | 6 | Cys776Ser | 1 | 1514 |
| SNP309 | T/G | MDM2 | MDM2 proto-oncogene | 12 | 5‘-FR | 1 | 85 |
| rs3781719 | T/C | CALCA | Calcitonin related polypeptide alpha | 11 | 5‘-FR | 1 | 137 |
| rs35815751 | 16bp delerion | CALCA | Calcitonin related polypeptide alpha | 11 | intron | 1 | 137 |
| rs45567233 | C/T | CTSG | Cathepsin G | 14 | Asn125Ser | 1 | 101 |
| rs1136410 | T/C | PARP1 | Poly(ADP-ribose) polymerase 1 | 1 | Val762Ala | 2 | 1100 |
| rs4926 | G/A | SERPING1 | Serpin family G member 1 | 11 | Val480Met | 2 | 1150 |
| CNV | >8/≤8 | DEFA1 | Defensin alpha 1 | 8 | - | 2 | 642 |
| rs736227 | A/G | DEFA4 | Ddefensin alpha 4 | 8 | 3'UTR | 2 | 903 |
| rs2741136 | A/G | DEFB1 | Defensin beta 1 | 8 | 5‘-FR | 1 | 368 |
| rs2738182 | A/T | DEFB1 | Defensin beta 1 | 8 | 5‘-FR | 1 | 368 |
| rs1799946 | G/A | DEFB1 | Defensin beta 1 | 8 | 5'UTR | 3 | 1234 |
| rs1800972 | C/G | DEFB1 | Defensin beta 1 | 8 | 5'UTR | 4 | 1853 |
| rs11362 | G/A | DEFB1 | Defensin beta 1 | 8 | 5'UTR | 2 | 942 |
| rs1130866 | C/T | SP-B | Surfactant protein B | 2 | Thr131Ile | 1 | 402 |
| rs1801252 | A/G | ADRB1 | Adrenoceptor beta 1 | 10 | Ser49Gly | 1 | 187 |
| rs1801253 | C/G | ADRB1 | Adrenoceptor beta 1 | 10 | Arg389Gly | 1 | 190 |
| rs1042713 | A/G | ADRB2 | Adrenoceptor beta 2 | 5 | Arg16Gly | 1 | 192 |
| rs1042714 | C/G | ADRB2 | Adrenoceptor beta 2 | 5 | Gln27Glu | 1 | 190 |
| rs4961 | G/T | ADD1 | Adducin 1 | 4 | Gly460Trp | 1 | 188 |
| rs4880 | C/T | SOD2 | Superoxide dismutase 2 | 6 | 5'UTR | 2 | 678 |
| rs61330082 | C/T | PBEF | Nicotinamide phosphoribosyltransferase | 7 | 5‘-FR | 2 | 438 |
| rs9770242 | T/G | PBEF | Nicotinamide phosphoribosyltransferase | 7 | 5‘-FR | 1 | 160 |
| rs1864183 | G/A | ATG10 | Autophagy related 10 | 5 | Thr/Met | 1 | 511 |
| rs3734114 | T/C | ATG10 | Autophagy related 10 | 5 | Pro/Ser | 1 | 510 |
| rs2241880 | T/C | ATG16L1 | Autophagy related 16 like 1 | 2 | Thr/Ala | 2 | 667 |
| rs77833427 | C/T | ATG2A | Autophagy related 2A | 11 | Arg/His | 1 | 511 |
| rs3759601 | C/G | ATG2B | Autophagy related 2B | 14 | Gln/Glu | 1 | 512 |
| rs74719094 | T/G | ATG2B | Autophagy related 2B | 14 | Arg/Ser | 1 | 512 |
| rs9323945 | C/T | ATG2B | Autophagy related 2B | 14 | Asn/Asp | 1 | 506 |
| rs2245214 | G/C | ATG5 | Autophagy related 5 | 6 | intron | 1 | 421 |
| rs548234 | C/T | ATG5 | Autophagy related 5 | 6 | intron | 1 | 803 |
| rs61733329 | C/T | ATG9B | Autophagy related 9B | 7 | Gly/Ser | 1 | 512 |
| rs4958847 | A/G | IRGM | Immunity related GTPase M | 5 | intron | 1 | 510 |
| rs72553867 | C/A | IRGM | Immunity related GTPase M | 5 | Thr/Lys | 1 | 512 |
| rs10065172 | C/T | IRGM | Immunity related GTPase M | 5 | Leu105Leu | 1 | 946 |
| rs9577229 | C/T | LAMP1 | Lysosomal-associated membrane protein 1 | 13 | Ala/Val | 1 | 491 |
| rs482912 | A/G | LAMP3 | Lysosomal-associated membrane protein 3 | 3 | Ile/Val | 1 | 511 |
| rs883541 | A/G | WIPI1 | WD repeat domain, phosphoinositide interacting | 17 | Thr31Ile | 1 | 510 |
| rs2393799 | T/C | P2RX7 | Purinergic receptor P2X 7 | 12 | intron | 1 | 512 |
| rs208294 | C/T | P2RX7 | Purinergic receptor P2X 7 | 12 | His155Tyr | 1 | 603 |
| rs28360457 | G/A | P2RX7 | Purinergic receptor P2X 7 | 12 | intron | 1 | 603 |
| rs2230912 | A/G | P2RX7 | Purinergic receptor P2X 7 | 12 | Gln460Arg | 1 | 603 |
| rs3751143 | A/C | P2RX7 | Purinergic receptor P2X 7 | 12 | Glu496Ala | 1 | 603 |
| rs1653624 | T/A | P2RX7 | Purinergic receptor P2X 7 | 12 | intron | 1 | 603 |
| rs16910526 | T/G | CLEC7A | C-type lectin domain family 7, member a | 12 | Tyr238Stop | 3 | 806 |
| rs2272658 | C/T | VPS28 | Vacuolar protein sorting 28 | 8 | intron | 1 | 596 |
| rs13258200 | A/C | CPSF1 | Cleavage and polyadenylation specific factor 1 | 8 | intron | 1 | 1252 |
| rs4380978 | G/C | CPSF1 | Cleavage and polyadenylation specific factor 1 | 8 | intron | 1 | 1230 |
| rs1585110 | C/T | RNF175 | Ring finger protein 175 | 4 | intron | 1 | 148 |
| rs4845320 | A/C | LCE4A | Late cornified envelope 4A | 1 | 5‘-FR | 3 | 560 |
| rs3127214 | C/T | TAGAP | T cell activation RhoGTPase activating protein | 6 | 5‘-FR | 3 | 556 |
| rs1883832 | C/T | CD40 | CD40 molecule | 20 | 5'UTR | 1 | 583 |
| rs17035850 | T/A | CD58 | CD58 molecule | 1 | 3'UTR | 3 | 553 |
| rs12025416 | T/A | CD58 | CD58 molecule | 1 | 3'UTR | 3 | 555 |
| rs1915087 | C/T | CD86 | CD86 antigen | 3 | 3'UTR | 2 | 667 |
| rs2332096 | T/G | CD86 | CD86 antigen | 3 | intron | 1 | 382 |
| rs1129055 | G/A | CD86 | CD86 antigen | 3 | Ala350Thr | 2 | 678 |
| rs17281995 | G/C | CD86 | CD86 antigen | 3 | 3'UTR | 1 | 393 |
| rs17602729 | C/T | AMPD1 | Adenosine monophosphate deaminase 1 | 1 | intron | 2 | 751 |
| rs653765 | C/T | ADAM10 | ADAM metallopeptidase domain 10 | 15 | 5'UTR | 1 | 890 |
| rs514049 | C/T | ADAM10 | ADAM metallopeptidase domain 10 | 15 | 5‘-FR | 1 | 890 |
| rs10273639 | T/C | PRSS1 | Serine protease 1 | 15 | intron | 1 | 106 |
| rs11216153 | G/T | APOA1 | Apolipoprotein A1 | 11 | 5‘-FR | 1 | 615 |
| rs2070665 | C/T | APOA1 | Apolipoprotein A1 | 11 | intron | 1 | 615 |
| rs632153 | G/T | APOA1 | Apolipoprotein A1 | 11 | intron | 1 | 615 |
| rs1050450 | C/T | GPX1 | Glutathione peroxidase 1 | 3 | Pro198Leu | 2 | 1272 |
| rs601338 | G/A | FUT2 | Fucosyltransferase 2 | 19 | Trp149Stop | 1 | 2406 |
| rs2910164 | C/G | MIR146A | MIR146A | 5 | - | 1 | 432 |
| rs57095329 | A/G | MIR146A | MIR146A | 5 | - | 1 | 427 |
| rs4919510 | G/C | MIR608 | MIR608 | 5 | - | 3 | 1268 |
| 4216T/C | T/C | ND1 | Mitochondrial NADH-ubiquinone oxidoreductase chain 1 | mtDNA | Tyr304His | 2 | 493 |
| Haplogroup R | R/non-R | mtDNA | Mitochondrial DNA | mtDNA | Haplogroup R | 1 | 751 |
| rs2228570 | C/T | VDR | Vitamin D receptor | 12 | Met1Thr | 4 | 484 |
| rs1544410 | C/T | VDR | Vitamin D receptor | 12 | intron | 1 | 120 |
| rs7975232 | T/G | VDR | Vitamin D receptor | 12 | intron | 2 | 324 |
| rs731236 | T/C | VDR | Vitamin D receptor | 12 | Ile352Ile | 4 | 484 |
| rs4516035 | T/C | VDR | Vitamin D receptor | 12 | 5‘-FR | 1 | 204 |
| rs11666254 | G/A | FPR2 | Formyl peptide receptor 2 | 19 | intron | 2 | 646 |
| rs17695052 | A/G | FPR2 | Formyl peptide receptor 2 | 19 | 3'UTR | 1 | 275 |
| rs17695064 | C/T | FPR2 | Formyl peptide receptor 2 | 19 | 3'UTR | 1 | 275 |
| rs1764390 | A/G | Cx37 | Gap junction protein alpha 4 | 1 | intron | 1 | 247 |
| rs1805005 | G/T | MC1R | Melanocortin 1 receptor | 16 | Val60Leu | 1 | 1246 |
| rs2228479 | A/G | MC1R | Melanocortin 1 receptor | 16 | Val92Leu | 1 | 1246 |
| rs1805007 | A/C | MC1R | Melanocortin 1 receptor | 16 | Arg151Cys | 1 | 1246 |
| rs885479 | A/G | MC1R | Melanocortin 1 receptor | 16 | Arg163Gln | 1 | 1246 |
| rs1805009 | A/C | MC1R | Melanocortin 1 receptor | 16 | Asp294His | 1 | 1246 |
| rs2228478 | A/G | MC1R | Melanocortin 1 receptor | 16 | Thr314Thr | 1 | 1246 |

*FR: flanking region, UTR: untranslated region; ^#^A/O haplotype: Combination of Arg52Cys, Gly54Asp, and Gly57GLu.
