# Supplementary material for: Host genetic variants in sepsis risk: a field synopsis and meta-analysis
Source: Crit Care. 2019 Jan 25;23:26. doi: 10.1186/s13054-019-2313-0 (PMC6347778; doi:10.1186/s13054-019-2313-0)
Supplement: Supplementary file 7 — Table S4. Genetic associations with sepsis risk investigated in fewer than three studies. (DOCX 39 kb) [file 13054_2019_2313_MOESM7_ESM.docx]

**Table S4. Genetic association studies with sepsis risk in less than three studies.**

| **Gene name** | **Polymorphisms** | **Author and publication year** | **Control type** | **Case** | **Control** | **Risk Allele/Genotype** | **OR95%CI** | **P** |
| --- | --- | --- | --- | --- | --- | --- | --- | --- |
| TLR1 | rs5743611 Arg80Thr | Plantinga 2012-1 | hospital patients | 245 | 263 | Arg/Thr+Thr/Thr | 1.82(1.12-2.98) | 0.02 |
| TLR1 | rs5743611 Arg80Thr | Plantinga 2012-2 | hospital patients | 93 | 88 | Arg/Thr+Thr/Thr | 2.92(0.23-155.70) | 0.62 |
|  |  | **Meta-analysis** |  | 338 | 351 | Arg/Thr+Thr/Thr | 1.87(1.16-3.01) | 0.01 |
| TLR4 | rs10116253-2242T/C | Chen 2010 | trauma patients | 160 | 143 | C | 2.06(1.03-4.10) | 0.04 |
| TLR4 | rs41426344+11367G/C | Duan 2009 | trauma patients | 79 | 53 | G | 0.43(0.22-0.84) | 0.01 |
| TLR4 | rs11536889G/C | Wang 2014 | healthy | 152 | 199 | C | 1.43(1.03-1.98) | 0.031 |
| TLR9 | rs352139+1174A/G | Chen 2011 | trauma patients | 207 | 344 | G | 1.38(1.07-1.76) | 0.012 |
| TLR9 | rs352162+6577T/C | Chen 2011 | trauma patients | 207 | 340 | C | 1.40(1.08-1.80) | 0.011 |
| TREM-1 | rs2234246G/A | Peng 2015 | healthy | 124 | 152 | AA | 3.10(1.15-8.32) | 0.02 |
| TREM-1 | rs2234246G/A | Su 2012 | ICU patients | 78 | 80 |  |  |  |
| NLRP3 | rs12048215+5134A/G | Zhang 2011 | trauma patients | 286 | 432 | A | 0.69(0.54-0.90) | 0.005 |
| NLRP3 | rs35829419 Gln705Lys | Asfaw Idosa 2014 | healthy | 60 | 1003 | Lys/Lys | 11.49(1.88-70.14) | 0.008 |
| CARD8 | rs2043211 Cys10Ter | Asfaw Idosa 2014 | healthy | 60 | 1003 | Ter | 2.00(1.40-2.90) | 0.0001 |
| CARD9 | rs4077515 Ser12Asn | Rosentul 2011-1 | hospital patients | 93 | 88 | Ser/Asn+Asn/Asn | 0.81(0.45-1.46) | 0.49 |
| CARD9 | rs4077515 Ser12Asn | Rosentul 2011-2 | hospital patients | 238 | 253 | Ser/Asn+Asn/Asn | 0.68(0.47-0.99) | 0.04 |
|  |  | **Meta-analysis** |  | 331 | 341 | Ser/Asn+Asn/Asn | 0.72(0.53-0.98) | 0.04 |
| CD14 | rs2569191-1145G/A | Gu 2008 | trauma patients | 42 | 63 | G | 0.18(0.04-0.82) | 0.026 |
| CD14 | rs2563298C/A | Wang 2014 | healthy | 152 | 198 | A | 2.45(1.46-4.13) | 0.001 |
| MD-2 | rs11465996-1625C/G | Zeng 2012-1 | trauma patients | 201 | 294 | G | 3.30(1.86-5.28) | 0.007 |
| MD-2 | rs11465996-1625C/G | Zeng 2012-2 | trauma patients | 90 | 141 | G | 1.77(1.12-2.79) | 0.01 |
|  |  | Meta-analysis |  | 291 | 435 | G | 1.91(1.49-2.46) | <0.0001 |
| LBP | rs5741812-921A/T | Flores 2009 | healthy | 175 | 357 | A | 0.58(0.39-0.87) | 0.009 |
| LBP | rs2232582+291T/C Pro97Pro | Chien 2008 | HCT patients | 97 | 204 | C | 2.15(1.31-3.52) | 0.002 |
| LBP | rs2232582+291T/C Pro97Pro | Barber 2003 | trauma patients | 37 | 114 |  |  |  |
| IRAK1 | rs1059702 Phe196Ser | Fang 2011 | healthy | 255 | 260 | Ser/Ser | 5.46(1.12-26.67) | 0.018 |
| IRAK3 | rs1152888 Val147Ile | Dong 2013 | healthy | 82 | 118 | Val | 0.10(0.06-0.16) | <0.0001 |
| IRAK3 | rs1152888 Val147Ile | Pino-Yanes 2011 | healthy | 214 | 336 | Val | 0.78(0.51-1.20) | 0.26 |
|  |  | **Meta-analysis** |  | 296 | 454 | Val | 0.31(0.22-0.42) | <0.0001 |
| IRAK4 | rs4251545 Ala304Thr | Yin 2012 | healthy | 192 | 192 | Thr | 1.69(1.10-2.58) | 0.015 |
| TOLLIP | rs5743867T/C | Fakhri 2016 | hospital patients | 23 | 85 | T | 0.62(0.28-1.38) | 0.24 |
| TOLLIP | rs5743867T/C | Song 2011 | healthy | 372 | 385 | T | 0.71(0.59-0.86) | 0.0006 |
|  |  | **Meta-analysis** |  | 395 | 470 | T | 0.66(0.54-0.81) | <0.0001 |
| TOLLIP | rs5743942T/C | Song 2011 | healthy | 373 | 386 | C | 1.40(1.03-1.88) | 0.034 |
| MYD88 | rs4988453C/G | Gökay 2016 | healthy | 65 | 65 | C/C | 0.26(0.11-0.59) | 0.001 |
| NFKBIL2 | rs760477-263C/T | Chapman 2010 | healthy children | 665 | 494 | C/C | 0.73(0.58-0.93) | 0.01 |
| REL | rs842647G/A | Pan 2015 | trauma patients | 300 | 433 | G | 0.67(0.53-0.87) | 0.012 |
| REL | rs842647G/A | Toubiana 2016 | ICU patients | 1040 | 855 |  |  |  |
| PPARG | rs10865710C/G | Gao 2016 | trauma patients | 300 | 434 | G | 1.46(1.16-1.84) | 0.001 |
| TNFA | rs1800610+489G/A | Kothari 2013 | healthy | 169 | 115 | A | 2.16(1.39-3.33) | 0.0006 |
| IL1B | rs1143623-1470G/C | Gu 2010 | trauma patients | 167 | 141 | C/C | 0.35(0.20-0.62) | 0.0003 |
| IL1B | rs1143643C/T | Esposito 2014 | pre-term neonates | 193 | 95 | T | 1.64(1.13-2.40) | 0.01 |
| IL4 | rs2243248-1098T/G | Gupta 2015 | trauma patients | 25 | 89 | T/G+G/G | 3.11(1.24-7.78) | 0.02 |
| IL4 | rs2243248-1098T/G | Henckaerts 2009 | ICU patients | 61 | 686 | T/G+G/G | 1.62(0.83-3.17) | 0.16 |
|  |  | **Meta-analysis** |  | 86 | 775 | T/G+G/G | 2.05(1.21-3.48) | 0.008 |
| IL12B | rs17860508-6415CTCTAA/GC | Stanilova 2010 | healthy | 43 | 125 | GC | 1.90(1.16-3.10) | 0.011 |
| IL21 | rs2055979G/T | Miao 2016 | healthy | 202 | 212 | G/T+T/T | 1.69(1.12-2.54) | 0.012 |
| IL21 | rs12508721C/T | Miao 2016 | healthy | 202 | 212 | C/T+T/T | 1.35(1.03-1.79) | 0.033 |
| IFNG | rs2069705-1616C/T | Wang 2014 | healthy | 196 | 213 | C/T+T/T | 1.84(1.24-2.73) | 0.002 |
| HMGB1 | rs2249825C/G | Qiu 2018 | healthy | 345 | 345 | G | 1.69(1.28-2.23) | 0.0002 |
| HMGB1 | rs2249825C/G | Zeng 2012 | trauma patients | 211 | 345 | G | 1.76(1.25-2.46) | 0.001 |
|  |  | **Meta-analysis** |  | 556 | 690 | G | 1.72(1.38-2.12) | <0.0001 |
| HMGB1 | rs1045411G/A | Qiu 2018 | healthy | 345 | 345 | A | 1.58(1.23-2.03) | 0.0004 |
| HMGB1 | rs1045411G/A | Zeng 2012 | trauma patients | 211 | 345 | A | 1.08(0.79-1.48) | 0.61 |
|  |  | **Meta-analysis** |  | 556 | 690 | A | 1.36(1.12-1.65) | 0.002 |
| HMGB1 | rs1360485A/G | Qiu 2018 | healthy | 345 | 345 | A/A+A/G | 0.44(0.20-0.94) | 0.03 |
| MCP-1 | rs1024611C/T | He 2017 | healthy | 403 | 400 | C/C+C/T | 0.52(0.38-0.72) | <0.0001 |
| MCP-1 | rs2857656A/C | He 2017 | healthy | 403 | 400 | A/A+A/C | 0.54(0.39-0.74) | 0.0002 |
| CXCR2 | rs10682383-665(AC)n | Flores 2006 | healthy | 178 | 357 | 24±1 repeat | 1.41(1.06-1.89) | 0.02 |
| CISH | rs414171A/T | Zhang 2018-1 | trauma patients | 338 | 468 | A | 0.77(0.63-0.94) | 0.01 |
| CISH | rs414171A/T | Zhang 2018-2 | trauma patients | 98 | 182 | A | 0.65(0.45-0.93) | 0.02 |
|  |  | **Meta-analysis** |  | 436 | 650 | A | 0.74(0.62-0.88) | 0.0007 |
| SOCS7 | rs3748726T/C | Zhang 2018 | trauma patients | 338 | 468 | T | 0.74(0.58-0.94) | 0.01 |
| NOS3 | rs2070744-786T/C | Martin 2014 | hospital patients | 88 | 91 | T/C+C/C | 1.96(1.06-3.62) | 0.03 |
| NOS3 | rs2070744-786T/C | Ma 2011 | healthy | 117 | 100 | T/C+C/C | 1.65(0.91-2.99) | 0.10 |
|  |  | **Meta-analysis** |  | 205 | 191 | T/C+C/C | 1.79(1.16-2.75) | 0.008 |
| NOS3 | rs1799983 Glu298Asp | Martin 2014 | hospital patients | 90 | 91 | Glu | 0.5(0.33-0.79) | 0.002 |
| NOS3 | rs1799983 Glu298Asp | Ma 2011 | healthy | 117 | 100 | Glu | 0.98(0.52-1.84) | 0.95 |
|  |  | **Meta-analysis** |  | 207 | 191 | Glu | 0.63(0.44-0.89) | 0.01 |
| ACE | rs4291A/C | Dou 2017 | healthy | 238 | 242 | C | 1.75(1.35-2.27) | <0.0001 |
| HLA-G | rs1710+3010G/C | Graebin 2012 | ICU patients | 469 | 169 | A/C+C/C | 1.92(0.84-4.41) | 0.12 |
| HLA-G | rs1710+3010G/C | Hahn 2017 | healthy | 64 | 190 | A/C+C/C | 2.77(1.25-6.14) | 0.01 |
|  |  | **Meta-analysis** |  | 533 | 359 | A/C+C/C | 2.27(1.26-4.09) | 0.007 |
| MMP-13 | rs2252070-77A/G | Martin 2014 | ICU patients | 90 | 91 | A/A | 0.50(0.28-0.90) | 0.02 |
| MMP16 | rs2664349+39811A/G | Esposito 2014 | pre-term neonates | 191 | 98 | GG | 3.54(1.17-10.72) | 0.03 |
| MMP16 | rs2664349+39811A/G | Davis 2010 | healthy | 28 | 52 |  |  |  |
| DEFA1 | CNV >8/≤8 | Chen 2010-1 | healthy | 179 | 233 | >8CNV | 0.36(0.24-0.54) | <0.0001 |
| DEFA1 | CNV >8/≤8 | Chen 2010-2 | healthy | 112 | 118 | >8CNV | 0.53(0.31-0.90) | 0.02 |
|  |  | **Meta-analysis** |  | 291 | 351 | >8CNV | 0.41(0.30-0.57) | <0.0001 |
| ADD1 | rs4961 Gly460Trp | Bunker-Wiersma 2008 | healthy | 53 | 135 | Trp | 2.10(1.11-4.04) | <0.02 |
| PBEF | rs61330082-1543C/T | Liu 2012 | healthy | 107 | 150 | C | 0.69(0.49-0.99) | 0.04 |
| PBEF | rs61330082-1543C/T | Ye 2005 | healthy | 98 | 83 | C | 0.71(0.45-1.13) | 0.15 |
|  |  | **Meta-analysis** |  | 205 | 233 | C | 0.70(0.53-0.93) | 0.01 |
| ATG16L1 | rs2241880 Thr/Ala | Savva 2014 | VAP patients | 58 | 97 | Ala | 2.40(1.06-5.60) | 0.036 |
| ATG16L1 | rs2241880 Thr/Ala | Smeekens 2013 | healthy | 275 | 237 |  |  |  |
| RFP175 | rs1585110C/T | Esposito 2016 | ICU patients | 49 | 99 | C/T+T/T | 2.92(1.07-7.95) | 0.04 |
| CD40 | rs1883832C/T | Liu 2018 | healthy | 261 | 322 | C/T+T/T | 1.84(1.29-2.62) | 0.0008 |
| CD86 | rs1915087C/T | Wang 2015 | healthy | 186 | 196 | C | 0.65(0.48-0.87) | 0.003 |
| CD86 | rs1915087C/T | Fu 2018 | healthy | 134 | 151 | C | 0.63(0.45-0.88) | 0.006 |
|  |  | **Meta-analysis** |  | 320 | 347 | C | 0.64(0.51-0.79) | <0.0001 |
| CD86 | rs2332096T/G | Wang 2015 | healthy | 186 | 196 | G | 1.65(1.21-2.24) | 0.001 |
| CD86 | rs1129055+1057G/A | Song 2015 | healthy | 192 | 201 | G | 0.64(0.48-0.84) | 0.002 |
| CD86 | rs1129055+1057G/A | Fu 2018 | healthy | 134 | 151 | G | 0.63(0.45-0.88) | 0.006 |
|  |  | **Meta-analysis** |  | 326 | 352 | G | 0.63(0.51-0.78) | <0.0001 |
| CD86 | rs17281995+2379G/C | Song 2015 | healthy | 192 | 201 | C | 1.75(1.04-2.95) | 0.033 |
| VDR | rs1544410C/T | Das 2016 | healthy | 60 | 60 | T | 2.62(1.55-4.42) | 0.0003 |
| FPR2 | rs11666254G/A | Zhang 2017-1 | trauma patients | 84 | 191 | A | 1.64(1.14-2.36) | 0.008 |
| FPR2 | rs11666254G/A | Zhang 2017-2 | trauma patients | 115 | 256 | A | 1.60(1.17-2.19) | 0.003 |
|  |  | **Meta-analysis** |  | 199 | 447 | A | 1.62(1.27-2.05) | <0.0001 |
| Cx37 | rs1764390A/G | Yu 2017 | healthy | 112 | 135 | G | 1.83(1.28-2.61) | 0.001 |
| MC1R | rs885479A/G | Seaton 2017 | ICU patients | 290 | 956 | A | 0.40(0.24-0.66) | 0.0004 |
| AMPD1 | rs17602729+34C/T | Ramakers 2015-1 | hospital patients | 304 | 101 | C/T+T/T | 2.35(1.32-4.17) | 0.004 |
| MIR146A | rs2910164C/G | Shao 2014 | healthy | 226 | 206 | C/G+G/G | 1.51(1.03-2.21) | 0.035 |
| ND1 | 4216T/C | Huebinger 2010 | burn patients | 32 | 143 | C | 2.81(1.24-6.36) | 0.01 |
| ND1 | 4216T/C | Gomez 2009 | trauma patients | 34 | 102 | C | 3.18(1.12-9.06) | 0.03 |
|  |  | **Meta-analysis** |  | 66 | 245 | C | 2.94(1.55-5.61) | 0.001 |

HCT: allogeneic hematopoietic cell transplantation; VAP: ventilator-associated pneumonia.
